# EZH2 deletion does not affect acinar regeneration but restricts progression to pancreatic cancer in mice

**DOI:** 10.1172/jci.insight.173746

**Published:** 2024-12-31

**Authors:** Emilie Jaune-Pons, Xiaoyi Wang, Fatemeh Mousavi, Zachary Klassen, Abdessamad El Kaoutari, Kurt Berger, Charis Johnson, Mickenzie B. Martin, Saloni Aggarwal, Sukhman Brar, Muhammad Khalid, Joanna F. Ryan, Parisa Shooshtari, Angela J. Mathison, Nelson Dusetti, Raul Urrutia, Gwen Lomberk, Christopher L. Pin

**Affiliations:** 1 Department of Physiology and Pharmacology and; 2 Department of Oncology, Schulich School of Medicine and Dentistry, Western University, London, Ontario, Canada.; 3 Verspeeten Family Cancer Centre, London, Ontario, Canada.; 4 Centre de Recherche en Cancérologie de Marseille (CRCM), Unité 1068, Institut National de la Santé et de la Recherche Médicale, Marseille, France.; 5 Department of Pediatrics and; 6 Department of Pathology and Laboratory Medicine, Schulich School of Medicine and Dentistry, Western University, London, Ontario, Canada.; 7 Linda T. and John A. Mellowes Center for Genomic Sciences and Precision Medicine, Medical College of Wisconsin, Milwaukee, Wisconsin, USA.; 8 Department of Surgery, Division of Research, Medical College of Wisconsin, Milwaukee, Wisconsin, USA.; 9 Department of Pharmacology and Toxicology, Medical College of Wisconsin, Milwaukee, Wisconsin, USA.

**Keywords:** Oncology, Cancer, Epigenetics, Mouse models

## Abstract

Enhancer of zeste homologue 2 (EZH2) is part of the Polycomb Repressor Complex 2, which promotes trimethylation of lysine 27 on histone 3 (H3K27me3) and gene repression. EZH2 is overexpressed in many cancers, and studies in mice attributed both prooncogenic and tumor suppressive functions to EZH2 in pancreatic ductal adenocarcinoma (PDAC). EZH2 deletion enhances de novo KRAS-driven neoplasia following pancreatic injury, while increased EZH2 expression in patients with PDAC is correlated to poor prognosis, suggesting a context-dependant effect for EZH2 in PDAC progression. In this study, we examined EZH2 in pre- and early neoplastic stages of PDAC. Using an inducible model to delete the SET domain of EZH2 in adult acinar cells (EZH2^ΔSET^), we showed that loss of EZH2 activity did not prevent acinar cell regeneration in the absence of oncogenic KRAS (KRAS^G12D^) nor did it increase PanIN formation following KRAS^G12D^ activation in adult mice. Loss of EZH2 did reduce recruitment of inflammatory cells and, when combined with a more aggressive PDAC model, promoted widespread PDAC progression and remodeling of the tumor microenvironment. This study suggests that expression of EZH2 in adult acinar cells restricts PDAC initiation and progression by affecting both the tumor microenvironment and acinar cell differentiation.

## Abstract 2

Oncogenic KRAS promotes epigenetic reprogramming prior to transformation. This process is mediated in part by EZH2 and targets genes related to the tumour microenvironment.

## Graphical abstract

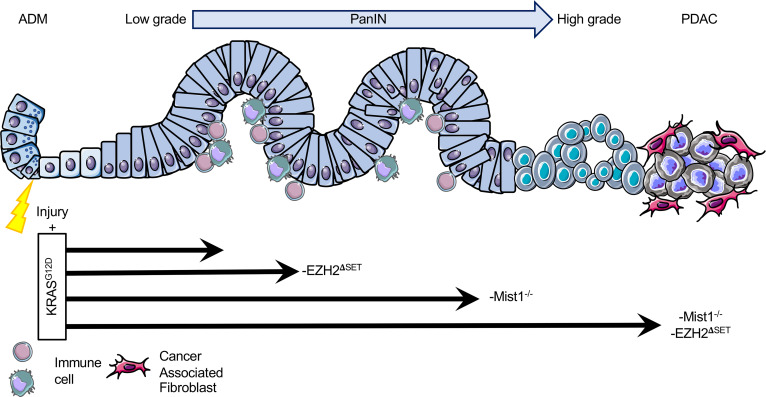

## Introduction

Pancreatic ductal adenocarcinoma (PDAC) is the most common form of pancreatic cancer with the worst 5-year survival, ~12%, of any of the major cancers (Pancreatic Cancer Facts, PANCAN). The principal driver mutation in PDAC is activating *KRAS* mutations, which occurs in > 90 % of patients with PDAC (1). Oncogenic KRAS mutations, such as KRAS^G12D^, appear at early stages of the disease but are not enough to induce PDAC on their own (2, 3). Several studies indicate that environmental stressors, in addition to somatic mutations in *KRAS,* are required for PDAC progression. Chronic inflammation is associated with increased sensitivity to KRAS^G12D^, indicating that environmental factors contribute to progression (4). Based on these findings, there is increasing interest in the role epigenetic mediators have in initiation and progression of PDAC. Mutations in several genes encoding epigenetic modifiers, including *ARID1A* and *KMT2D* (5), are found in patients with PDAC, and activation of *KRAS^G12D^* is associated with extensive changes in the epigenetic profile of cells (6). In addition, Enhancer of zeste homolog 2 (EZH2) is highly expressed in a subset of PDAC tumors and correlated to poor prognosis (7).

EZH2 is a histone-lysine N-methyltransferase enzyme and part of the Polycomb repressive complex 2 (PRC2), which plays a critical role in cell fate specification during embryonic development (8, 9). EZH2 induces trimethylation of H3K27me3 (K27me3), a histone modification linked to chromatin remodeling and gene repression (10). EZH2 is overexpressed in many cancers (7, 11), but both prooncogenic and tumor suppressive roles have be reported in the context of PDAC (12, 13). In the developing pancreas, EZH2 establishes long-term gene expression profiles, and deletion of the SET domain — which is responsible for methyltransferase activity — reduces acinar cell regeneration after injury and increases pancreatic intraepithelial neoplasia (PanIN) initiation and tumor progression (12). Using a similar mouse model, loss of EZH2 methyltransferase activity during development along with expressing KRAS^G12D^ initially favored PanIN progression but reduced PanIN maintenance in aged mice compared with KRAS^G12D^ alone (14). This study proposed a role for EZH2 in NFATc1 regulation and PDAC progression, suggesting that EZH2’s role may extend to the tumor microenvironment (14). More recent studies show that *EZH2* deletion in pancreatic cancer cells increased GATA6 expression, a marker of classical PDAC subtype, indicating the presence of EZH2 promotes a more aggressive, basal-like PDAC subtype (13). Coupled with the findings that increased EZH2 expression correlates to more advanced disease and increased therapeutic resistance (15, 16), it appears that EZH2’s role differs between early stages of PDAC initiation and later progression and resistance.

In this study, we examined the function of EZH2 in preneoplastic stages of PDAC, focusing on EZH2’s effect on acinar cell regeneration and PanIN initiation in adult mice. Since patients with PDAC present later in life (>60 years of age), we used a preclinical model that allows KRAS^G12D^ induction in adult acinar cells of the pancreas, instead of embryonic induction of KRAS^G12D^ (12, 14). We employed a similar approach to alter EZH2 function, in which the SET domain of EZH2 (EZH2^ΔSET^) is deleted, but we used an inducible Cre recombinase that promoted deletion in only acinar cells of the adult pancreas. Unlike previous studies, our results indicate that loss of EZH2 activity has a minimal effect on acinar cell regeneration and does not enhance PanIN initiation, but it initially favors more advanced PanIN lesion development in the context of KRAS^G12D^. Loss of EZH2 SET activity in combination with KRAS^G12D^ induces reprogramming of the genome based on K27me3 enrichment and reduces immune cell recruitment in response to injury. Conversely, deleting EZH2^ΔSET^ in a susceptible mouse model for PDAC (*Mist1^creERT/–^ KRAS^G12D^*) greatly enhanced PanIN progression and PDAC formation. This study highlights several context-dependent roles for EZH2 in PDAC initiation and progression. EZH2 helps mediate *KRAS^G12D^*-induced reprogramming of the acinar cell genome; primes immune and inflammatory genes in these cells, which allows for a differential immune response; and is required for long-term expansion of preneoplastic lesions.

## Results

### KRAS^G12D^ promotes widespread epigenetic remodeling in acinar cells.

**Figure 1:**
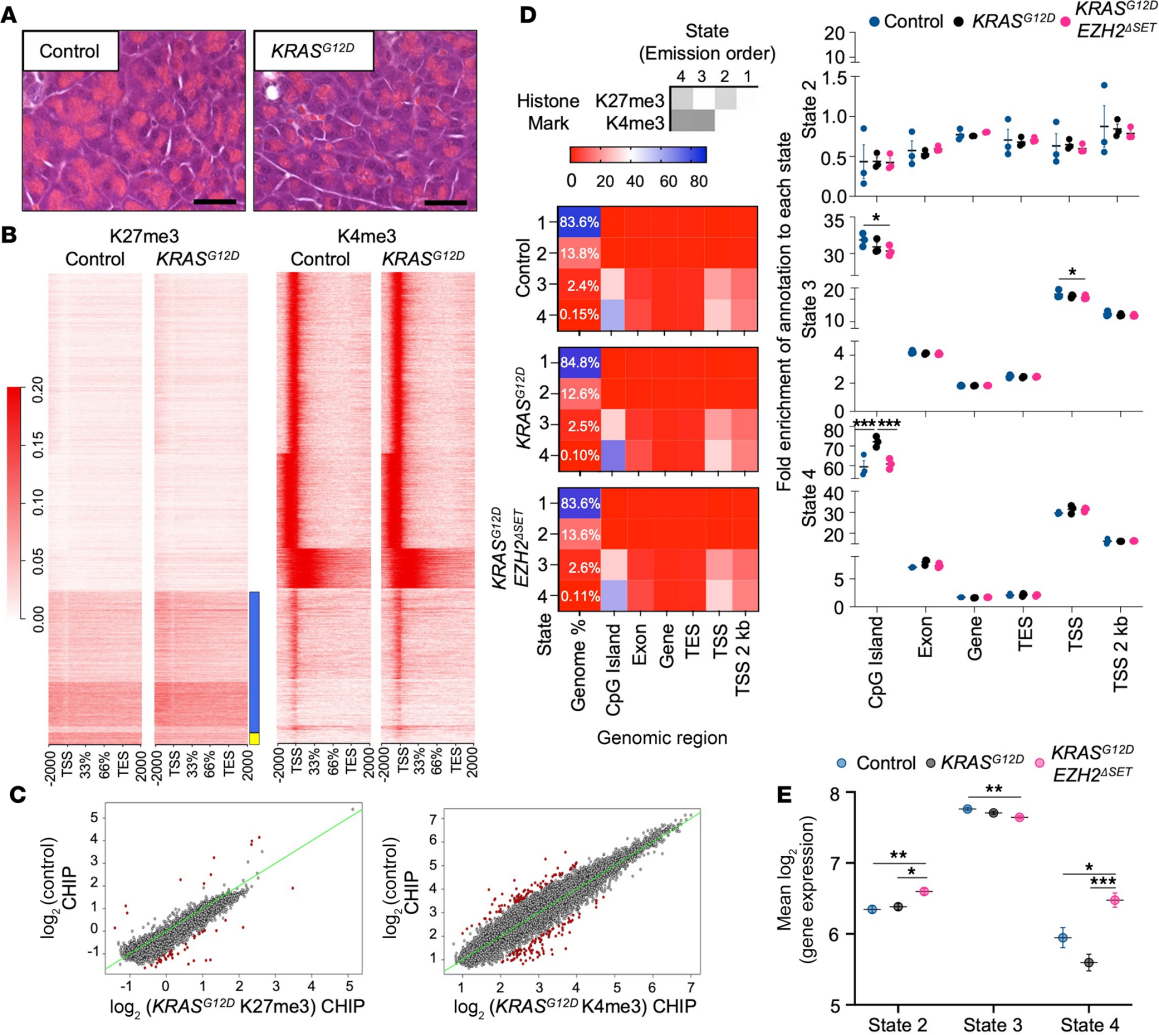
*KRAS^G12D^* promotes increased K27me3 enrichment in pancreatic acini. (**A**) Representative images of H&E-stained pancreatic tissue from control and *KRAS^G12D^* mice 22 days after TX gavage. Scale bar: 50 μm. (**B**) Heatmaps show K27me3 and K4me3 enrichment from 2 kb before the transcriptional start sites (TSS) to 2 kb after the transcriptional end site (TES) of all genes. Blue and yellow boxes indicate areas showing increased or decreased K27me3 enrichment in *KRAS^G12D^* mice. There is reduced K27me3 at TSSs, which appears restricted in *KRAS^G12D^* mice. (**C**) Comparison of called K27me3 and K4me3 enrichment at TSSs in control and *KRAS^G12D^* acinar cells. Red dots represent genes with significantly dysregulated enrichment. Green line indicates expectation for equal enrichment between genotypes. (**D**) Comparison of chromatin states in control, *KRAS^G12D^,* and *KRAS^G12D^EZH2*^ΔSET^ mice 22 days after KRAS^G12D^ induction based on K4me3 and K27me3 enrichment. Numbers in first column indicate the percentage of each state across of the genome. Graphs show quantification of these numbers at the different gene regions. (**E**) Correlation between gene expression and chromatin states in control, *KRAS^G12D^,* and *KRAS^G12D^EZH2*^ΔSET^ pancreata 22 days after KRAS^G12D^ induction. Data represent mean ± SEM (*n* = 3 mice /group). Two-way ANOVA followed by Tukey’s post hoc test was performed. *P < 0.05; **P < 0.01; ***P < 0.001.

To examine the epigenetic response to oncogenic KRAS, KRAS^G12D^ expression was induced in acinar cells of 2- to 4-month-old *Mist1^creERT/+^KRAS^G12D^* mice (referred as *KRAS^G12D^*) by tamoxifen (TX) gavage (Supplemental Figure 1A; supplemental material available online with this article; https://doi.org/10.1172/jci.insight.173746DS1). Twenty-two days after KRAS^G12D^ activation, H&E histology showed no differences in acinar cell morphology (Figure 1A). ChIP-Seq for K27me3 and H3K4me3 (K4me3) was performed on whole pancreatic tissue, since these marks are linked to gene repression and activation and can maintain genes in a primed state (17). Such priming has been identified in pancreatic development and adult tissue (18–20) and involves enrichment of K4me3 and K27me3 at the same genomic regions (21, 22).

The total number of K4me3-enriched regions decreased slightly (~1.1%) in *KRAS^G12D^* tissue, while the number of sites enriched for K27me3 was substantially higher (~38.5%) in *KRAS^G12D^* pancreata (*n* = 3 mice/genotype; Table 1). The distribution of K4me3 and K27me3 enrichment within the genome was similar between genotypes (Supplemental Figure 1B), and heatmaps confirmed little change in K4me3 enrichment around genes (Figure 1B). Heatmaps for K27me3 suggested some uniquely enriched transcription start sites (TSSs) in *KRAS^G12D^* and control pancreata (Figure 1B). Comparing TSSs between genotypes supported a general increase in K27me3 enrichment in *KRAS^G12D^* pancreata (Figure 1C), while changes in K4me3-enriched TSSs, which were more numerous, were uniformly distributed between the 2 genotypes (Figure 1C).

To determine regions of gene priming in the acinar genome, we classified chromatin states based on K27me3 and K4me3 marks. We defined 4 distinct states: state 1 is absent for both marks, state 2 (K27me3) and state 3 (K4me3) contain single marks, and state 4 — the primed state — contains both (Figure 1D). Distribution of states 2–4 did not change (Supplemental Figure 1C) between genotypes, with most state 2 regions located distally from genes and states 3 and 4 closely associated with gene bodies. Correlation to transcriptomic data obtained from the same pancreatic samples confirmed that state 4 enrichment at CpG islands is associated with reduced expression compared with genes in state 3 and resembled expression of genes associated with state 2 (Figure 1E). *KRAS^G12D^* tissue showed a marked increase in state 4–enriched CpG islands relative to control tissue, which are associated with both enhancer regions and gene regulation (Figure 1D). These findings suggest that KRAS^G12D^ expression promotes increased K27me3 enrichment in acinar cells and can affect epigenetically primed regions within the genome.

### EZH2 methyltransferase restricts KRAS^G12D^-mediated PanIN progression following injury.

Since K27me3 involves EZH2 (23, 24), we examined the effects of deleting EZH2 methyltransferase activity in the context of KRAS^G12D^. A similar model to previous studies was used, with loxP sites flanking *Ezh2* exons 16–19, which encompasses the SET domain (12, 25). We employed a *Mist1* Cre driver that allows inducible and acinar-specific *Ezh2* deletion and KRAS^G12D^ activation in adult acinar cells (Supplemental Figure 2A). To induce PanIN formation, activation of KRAS^G12D^ expression was combined with a 2-day cerulein regimen, 15 and 17 days after initial KRAS^G12D^ activation (Supplemental Figure 2B) (26). PanIN progression was compared 35 days after initial cerulein treatment in *KRAS^G12D^* and *Mist1^creERT/+^ KRAS^LSL-G12D^ Ezh2*^Δ*SET/*ΔSET^ (referred to as *KRAS^G12D^Ezh2*^ΔSET^) mice. C57BL/6 mice, or mice carrying only the *Mist1^creERT^* allele, were used as controls since loss of a single *Mist1* allele had no effect on gene expression (both indicated as control). We also included mice carrying the *Mist1^creERT^* allele and those homozygous for the *Ezh2*^ΔSET^ allele (*Mist1^creERT/+^ Ezh2*^ΔSET^, referred to as *EZH2*^ΔSET^). No group showed overt differences based on final weights, regardless of whether mice were treated with cerulein or saline (Supplemental Figure 2C). Similarly, pancreatic weight as a percentage of body weight showed no differences at the time of dissection (Supplemental Figure 2D).

Histological analysis of control and *EZH2*^ΔSET^ pancreatic tissue showed no differences in pancreatic morphology (Supplemental Figure 2E), as opposed to previous studies, which suggested EZH2 was required for acinar cell regeneration (12). Since this study used a longer, recurrent model of cerulein-induced pancreatitis (CIP), the response of *Ezh2*^ΔSET^ mice to twice daily injections of 250 μg/kg cerulein over 2 weeks was examined (Supplemental Figure 3A) (27). As previously reported, increased EZH2 accumulation was observed in response to injury in tissue from control animals, with EZH2 completely absent in *Ezh2*^ΔSET^ tissue (Supplemental Figure 3B). However, recurrent injury still showed no differences in body weight (Supplemental Figure 3C) or pancreas/body weight ratios (Supplemental Figure 3D), pancreatic morphology (Supplemental Figure 3E), or amylase accumulation (Supplemental Figure 3, B and F) between genotypes. Both control and *EZH2*^ΔSET^ pancreatic tissue had increased CK19 accumulation following CIP (Supplemental Figure 3G) with no difference in accumulation between genotypes. This suggests that acinar cell regeneration is not restricted upon EZH2^ΔSET^ deletion in mature acinar cells. Thus, we returned to the acute-CIP model to assess the effect of *Ezh2*^ΔSET^ deletion on KRAS^G12D^-mediated PanIN progression.

**Figure 2:**
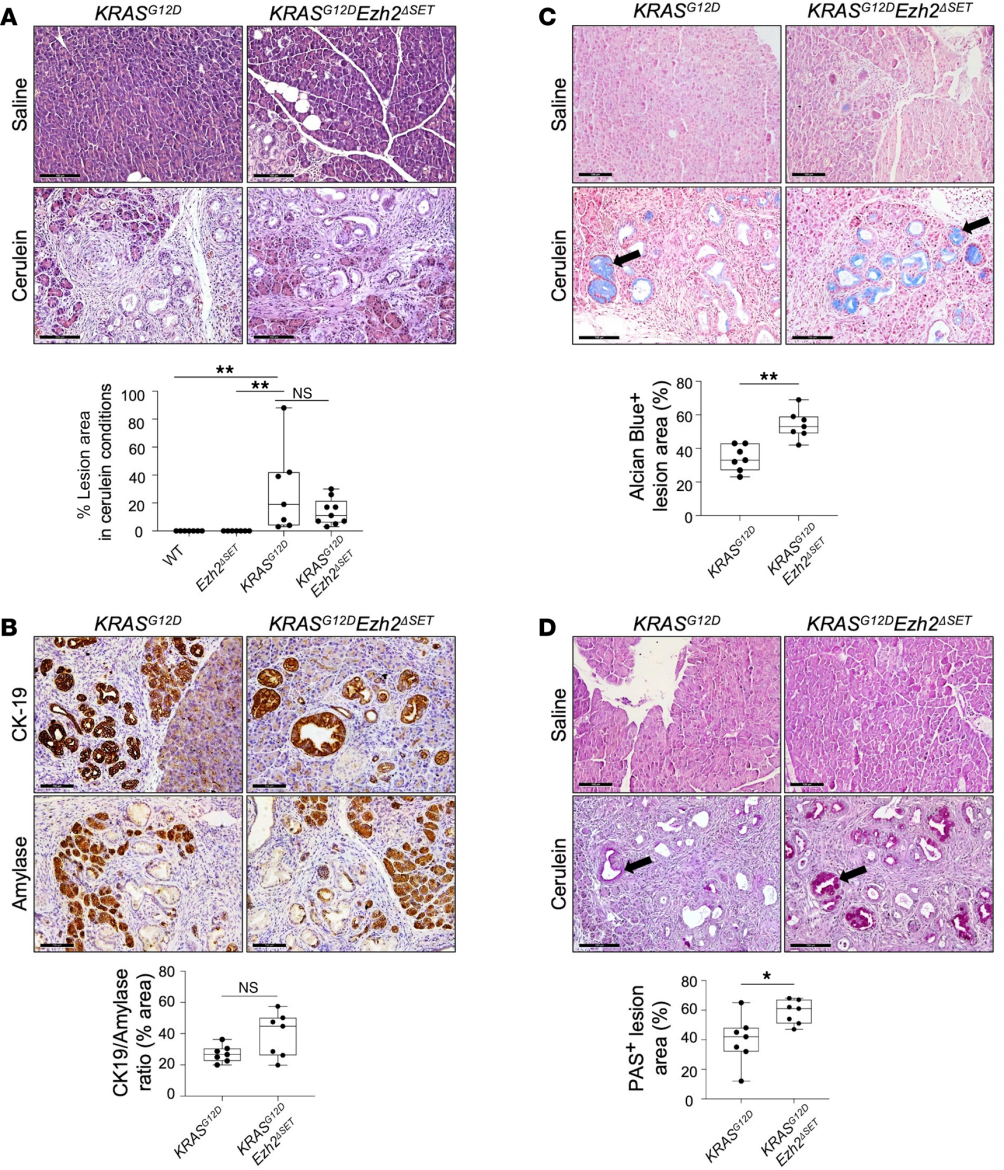
Loss of EZH2 methyltransferase activity increases *KRAS^G12D^*-mediated PanIN progression. Histological and quantitative analysis comparing *KRAS^G12D^* and *KRAS^G12D^Ezh2*^ΔSET^ mice 51 days after initiating KRAS^G12D^ and 35 days after treatment with saline or cerulein. (**A**) Representative H&E images of pancreatic tissue. Box plots indicate the amount of lesion area as a percentage of the entire pancreatic tissue. Significance was measured by 1-way ANOVA followed by Tukey’s post hoc tests. (**B**) Representative IHC for CK19 or amylase followed by counterstaining with hematoxylin in cerulein-treated mice. Box plots compare the ratio of CK19^+^/amylase^+^ tissue. Significance was measured by 2-tailed unpaired Mann-Whitney *U* test. (**C** and **D**) Representative images of alcian blue histology (**C**) or periodic acid–Schiff (PSA) (**D**) histology showing advanced lesions (arrows) in saline- or cerulein-treated *KRAS^G12D^* and *KRAS^G12D^Ezh2*^ΔSET^ mice. Box plots compare the stained area as a percentage of ADM/PanIN lesions. Significance was measured by 2-tailed unpaired Mann-Whitney *U* test. Scale bar: 100 μm. For graphs, individual mice (*n* = 7 mice per group) are shown and data represent mean ± minimum to maximum. *P ≤ 0.05, **P ≤ 0.01.

Five weeks after KRAS^G12D^ activation, saline-treated *KRAS^G12D^* and *KRAS^G12D^Ezh2*^ΔSET^ pancreatic tissue showed sporadic lesions (<1% of the entire tissue area). Cerulein treatment resulted in intralobular lesions containing acinar to duct cell metaplasia (ADM) and PanINs in *KRAS^G12D^* expressing tissue. While *KRAS^G12D^* mice had more lesions (29% ± 11.5%) than *KRAS^G12D^Ezh2*^ΔSET^ (13.7% ± 3.2%) mice, the difference was not significant (Figure 2A and Supplemental Figure 4A). To quantify ADM and PanINs, we compared the ratio of CK19 (marker of ADM and PanINs) to amylase accumulation (Figure 2B). While a trend toward increased CK19 accumulation in *KRAS^G12D^Ezh2*^ΔSET^ mice was observed, it was not significant compared with *KRAS^G12D^* mice (*P* = 0.219). However, measures of PanIN progression, including Alcian blue (Figure 2C and Supplemental Figure 4B) and periodic acid–Schiff (PAS) histology (Figure 2D and Supplemental Figure 4C), showed that *KRAS^G12D^Ezh2*^ΔSET^ mice had significantly more staining of PanINs compared with *KRAS^G12D^* mice, suggesting EZH2 limited progression to more advanced PanIN lesions.

Next, K27me3 enrichment was assessed in *KRAS^G12D^Ezh2*^ΔSET^ tissue 22 days after KRAS^G12D^ activation and prior to injury induction, and it was integrated with earlier analysis of *KRAS^G12D^* and control pancreatic tissue (Table 1). At this time point, pancreatic tissue retained normal histology in all genotypes (Figure 1A and Supplemental Figure 5A). The number of K27me3 enrichment sites was higher (8.3% increase) in *KRAS^G12D^Ezh2*^ΔSET^ versus control tissues, but it was markedly lower (27.8% decrease) compared with *KRAS^G12D^* tissue, confirming that the absence of EZH2 methyltransferase activity reduced the ability of KRAS^G12D^ to reprogram the genome (Table 1). Analysis of K4me3 identified modest increases in the number of enriched sites in *KRAS^G12D^Ezh2*^ΔSET^ tissue compared with both control (+3.2%) and *KRAS^G12D^* tissue (+4.3%; Table 1).

To call genes targeted for reprogramming, enrichment peaks associated with gene bodies were identified. K27me3 enrichment typically occurs as broad local enrichments (BLOCs) that extend over 100 kb (28). Therefore, we called genes based on K27me3 sites between –100 kb and +3 kb from TSSs. This criterion identified substantially more K27me3-annotated genes in *KRAS^G12D^* over control tissue (+31.6%; Table 1). Conversely, *KRAS^G12D^Ezh2*^ΔSET^ tissue showed only a 9.8% increase in K27me3-enriched genes compared with control tissue, with 16.6% fewer K27me3-annotated genes than *KRAS^G12D^* tissue (Table 1 and Supplemental Figure 5B). Of the 1,515 genes enriched for K27me3 in *KRAS^G12D^* but not control tissue, less than half (692; 45.7%) were also enriched in *KRAS^G12D^Ezh2*^ΔSET^ tissue (Supplemental Figure 5C). Alternatively, K4me3 peaks are located close to TSSs, typically 1–4 kb in breadth (28), and we used a range of ± 3 kb from TSSs to call genes. The number of K4me3-enriched genes in control tissue was very similar to both *KRAS^G12D^* (+0.7%) and *KRAS^G12D^Ezh2*^ΔSET^ tissues (–0.4%), suggesting that loss of EZH2 methyltransferase activity does not affect enrichment of this mark (Table 1).

**Figure 3:**
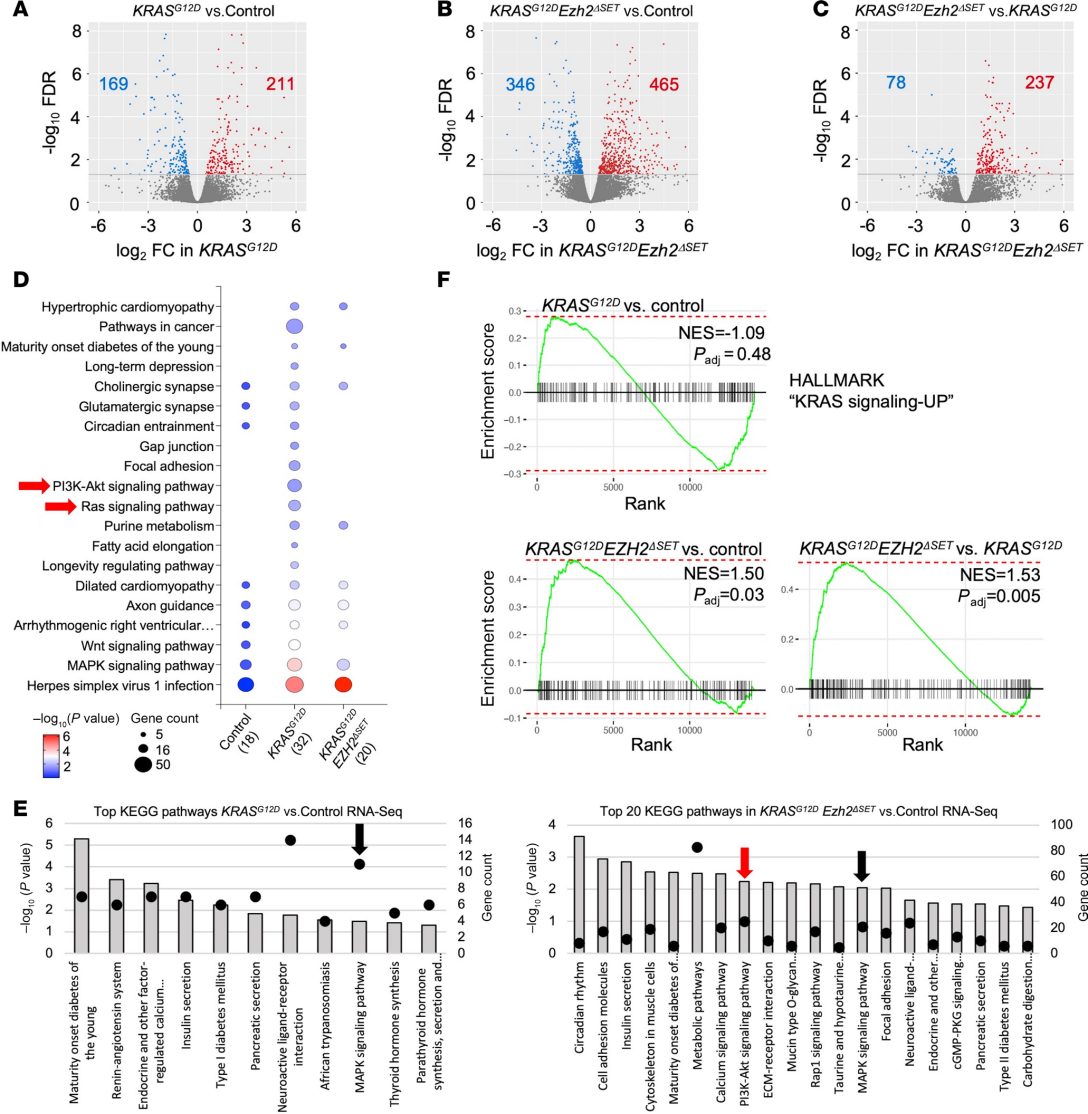
Loss of EZH2 methyltransferase activity alters the effects of *KRAS^G12^* on expression of genes linked to the tissue microenvironment. (**A**) Volcano plot of RNA-Seq analysis between control and *KRAS^G12D^* pancreata 22 days after TX gavage. Significantly downregulated genes are shown in blue and significantly upregulated genes in red. Significance was evaluated with FDR ≤ 0.05. (**B** and **C**) Similar Volcano plots comparing gene expression between *KRAS^G12D^Ezh2*^ΔSET^ and control (**B**) or *KRAS^G12D^* (**C**) pancreatic tissue 22 days after activating KRAS^G12D^ (*n* = 3 mice). (**D**) KEGG pathway analysis performed on genes enriched for K27me3 and K4me3 identifies an increase in the state 4 pathways in KRAS^G12D^ tissue (number of pathways) including unique enrichment of downstream mediators of KRAS signaling (red arrows). (**E**) KEGG pathway analysis based on DEGs from RNA-Seq identified enriched pathways between *KRAS^G12D^* (all pathways shown) or *KRAS^G12D^EZH2*^ΔSET^ (top 20 pathways shown) and control tissue. Bars indicate –log_10_ (*P* value), and dots indicate gene counts. Arrows indicate KRAS-related pathways unique (red) or common (black) to each data set. (**F**) Gene set enrichment analysis comparing enrichment of HALLMARK_KRAS_UP signaling between control, *KRAS^G12D^*, and *KRAS^G12D^Ezh2*^ΔSET^ tissue 22 days following tamoxifen treatment. Normalized enrichment scores (NES) are significantly different between *KRAS^G12D^Ezh2*^ΔSET^ and both control and *KRAS^G12D^* expression (*n* = 3).

We next assessed the effects of KRAS^G12D^ on acinar cell gene expression at the same 22-day time point. RNA-Seq analysis identified 380 differentially expressed genes (DEGs; Figure 3A and Supplemental Table 8) between control and KRAS^G12D^ tissue, markedly fewer changes when compared with changes in K27me3 enrichment. Interesting, combined loss of EZH2 methyltransferase activity with *KRAS^G12D^* had a more profound effect on gene expression than *KRAS^G12D^* alone. *KRAS^G12D^Ezh2*^ΔSET^ pancreatic tissue had 811 DEGs compared with control (Figure 3B and Supplemental Table 8) and 315 DEGs compared with *KRAS^G12D^* tissue (Figure 3C and Supplemental Table 8). To determine if changes in gene expression were related to epigenetic reprogramming, we integrated RNA-Seq data with the ChIP-Seq data. State 2 and 4 genes were examined as they represent targets of EZH2. For the great majority of state 2 genes uniquely enriched for K27me3 in each genotype, no corresponding changes in gene expression were observed (>97%; Supplemental Figure 6A), consistent with previous observations (29). Changes in gene expression rarely corresponded to uniquely called state 4 peaks within a genotype (Table 1), indicating that most epigenetic changes correlating to *KRAS^G12D^* activity were silent. In total, 2.8% of state 4 genes unique to control tissue were differentially expressed between control and either *KRAS^G12D^* or *KRAS^G12D^Ezh2*^ΔSET^ tissue (Table 1). Similarly, 1.7% of *KRAS^G12D^* and 4.3% of *KRAS^G12D^Ezh2*^ΔSET^ State 4 genes were also DEGs. Alignment with RNA-Seq data showed that state 4 genes in *KRAS^G12D^Ezh2*^ΔSET^ had lower expression patterns similar to state 2 genes (Figure 1E), but consistent with a role for EZH2 in KRAS^G12D^-mediated silencing, higher expression of state 2 and 4 genes was observed in *KRAS^G12D^Ezh2*^ΔSET^ tissue compared with control and *KRAS^G12D^* tissue (Figure 1E).

Alterations in state 4 genes suggests priming may lead to different responses to environmental cues. KEGG analysis of state 4 enriched genes showed that *KRAS^G12D^* tissue had many more uniquely enriched pathways compared with control or *KRAS^G12D^Ezh2*^ΔSET^ tissue including RAS and PI3K/Akt signaling (Figure 3D and Supplemental Table 1). While more pathways were enriched in *KRAS^G12D^* tissue, KEGG analysis using state 2 genes showed no difference in KEGG pathways between genotypes (Supplemental Figure 6B and Supplemental Table 2). Similar KEGG pathway analysis of DEGs between *KRAS^G12D^* or *KRAS^G12D^Ezh2*^ΔSET^ tissue and control tissue showed enrichment for MAPK signaling in both genotypes, but only *KRAS^G12D^Ezh2*^ΔSET^ tissue was enriched for P13K/Akt signaling (Figure 3E and Supplemental Table 3) and gene set enrichment analysis (GSEA) shows enhanced activation of KRAS-UP signaling (Figure 3F) and P13K/AKT signaling (Supplemental Figure 6C) in *KRAS^G12D^Ezh2*^ΔSET^ tissue compared with both control and *KRAS^G12D^* tissues. This supports a mechanism in which EZH2 restricts activation of KRAS-mediated pathways and may account for the more progressive PanINs observed in *KRAS^G12D^Ezh2*^ΔSET^ tissue.

**Figure 4:**
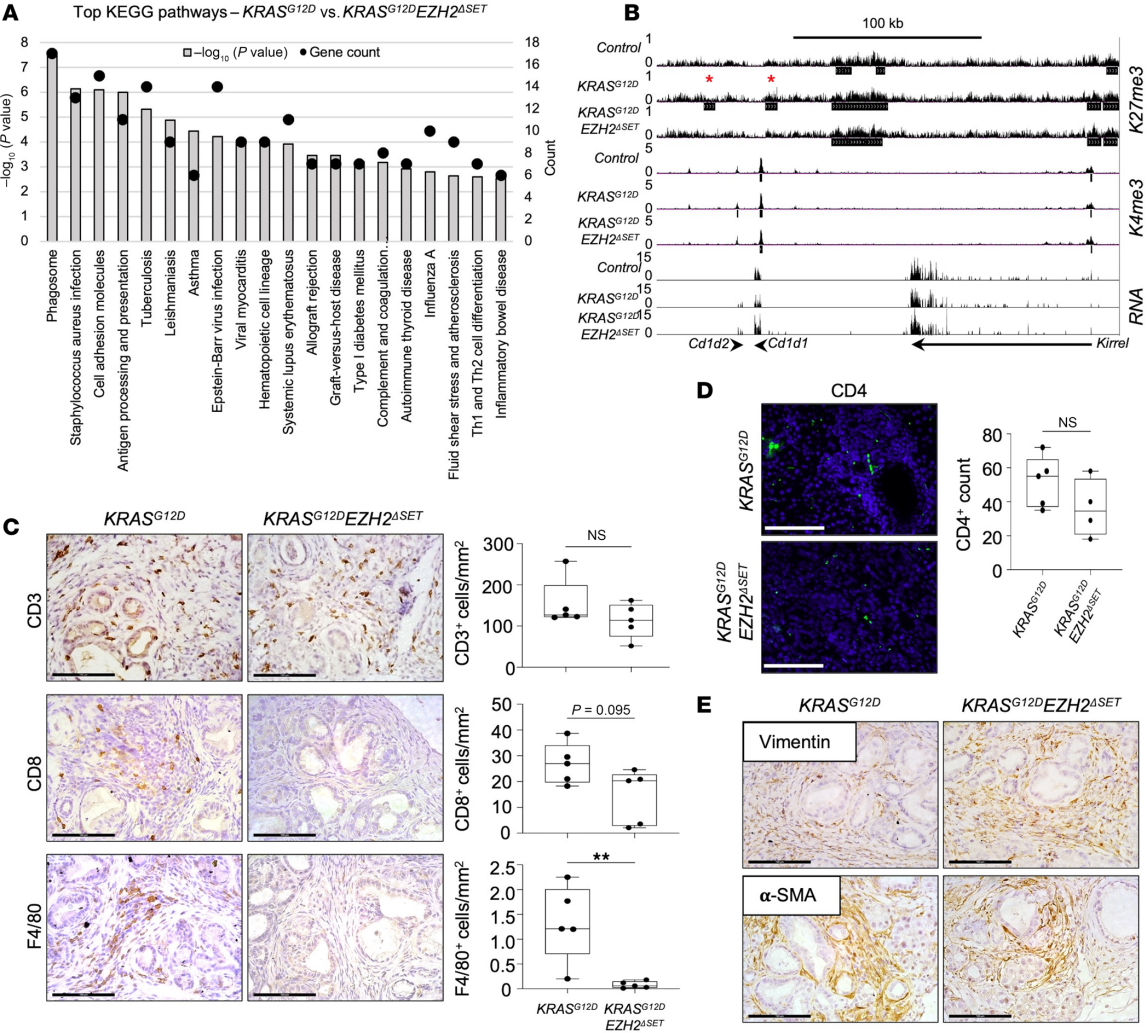
EZH2 deletion alters immune cell infiltration promoted by KRAS^G12D^ after acute cerulein treatment. (**A**) KEGG pathway analysis of DEGs between *KRAS^G12D^* and *KRAS^G12D^Ezh2*^ΔSET^ pancreatic tissue 22 days after tamoxifen treatment. Bars indicate the FDR values, while black dots indicate the number of genes associated with each pathway. (**B**) K27me3, K4me3, and RNA tracks showing bivalency and differential K27me3 enrichment between *KRAS^G12D^* and control or *KRAS^G12D^Ezh2*^ΔSET^ at *Cd1d2*. Red asterisks indicate K27me3 enrichment specific to *KRAS^G12D^* mice. Tracks are an overlay of *n* = 3 mice. (**C** and **D**) IHC for CD3, CD8, and F4/80 (**C**) or IF for CD4^+^ cells (**D**) in pancreatic tissue from *KRAS^G12D^* and *KRAS^G12D^Ezh2*^ΔSET^ mice 51 days after expressing KRAS^G12D^ and 35 days following cerulein treatment. Scale bar: 100 μm. Box plots compare the mean number of positive cells, and individual values (*n* = 5 mice per condition) are included. Data are shown as mean ± minimum to maximum. Significance was measured using a 2-tailed unpaired Mann-Whitney *U* test. **P ≤ 0.01. (**E**) Representative images of IHC for vimentin or α-SMA staining on pancreatic tissue. Scale bar: 100 μm.

Direct comparison of *KRAS^G12D^* and *KRAS^G12D^Ezh2*^ΔSET^ transcriptomics also identified immune-related pathways as differentially enriched (Figure 4A and Supplemental Table 4). While previous studies suggest that KRAS works through EZH2 and NFATc1 to affect an inflammatory response (30), RNA-Seq analysis showed no differences in *Nfatc1* expression between genotypes (Supplemental Figure 7A) and immune cell infiltration based on CD3^+^ (T lymphocytes), CD4^+^ (Th cells), CD8^+^ (cytotoxic T cells), and F4/80^+^ (macrophages) expression was not observed in either *KRAS^G12D^* and *KRAS^G12D^Ezh2*^ΔSET^ tissue without cerulein treatment (Supplemental Figure 7B). However, several DEGs in the immune-related pathways, including *Cd1d1* (Figure 4B), *Colec12*, *Maf*, *H2-Q6*, and *H2-Q7* (Supplemental Figure 7C), showed K27me3 enrichment peaks in *KRAS^G12D^* but not *KRAS^G12D^Ezh2*^ΔSET^ tissue.

Examination 5 weeks after acute cerulein injury showed accumulation of CD3^+^, CD4^+^, CD8^+^, and F4/80^+^ cells surrounding PanIN lesions in *KRAS^G12D^* tissue (Figures 4, C and D). While *KRAS^G12D^Ezh2*^ΔSET^ tissue had similar accumulation of CD3^+^ and CD4^+^ cells, a significant reduction in F4/80^+^ cells (*P* < 0.001) and a trend toward decreased CD8^+^ cells (*P* = 0.095) was observed (Figure 4C). Similar analysis for vimentin and α-SMA, markers of cancer-associated fibroblasts, showed no difference between *KRAS^G12D^* and *KRAS^G12D^Ezh2*^ΔSET^ mice (Figure 4E). Combined, these data suggest an EZH2-dependent mechanism in which KRAS^G12D^ reprograms the acinar cell epigenome and affects infiltration of immune cells upon injury. 

### Loss of EZH2 activity promotes rapid progression of PDAC in Mist1^creERT/–^ KRAS^G12D^ model.

Our findings on EZH2’s role in early PanIN progression differed from previous studies. One possibility is EZH2’s role differs depending on the susceptibility of the model to KRAS^G12D^. Therefore we assessed whether EZH2 showed a similar ability to restrict PanIN progression in a more severe model of PDAC. We generated *Mist1^creERT/creERT^KRAS^G12D^* mice with (indicated as *Mist1^creERT/–^ KRAS^G12D^*) and without the EZH2 ΔSET domain (*Mist1^creERT/–^ KRAS^G12D^Ezh2*^ΔSET^; indicated as *MKE*; Supplemental Figure 8A) as loss of MIST1 markedly increases sensitivity to KRAS^G12D^ (31). Gross morphological analysis 2 months after KRAS^G12D^ activation (Supplemental Figure 8B) revealed no differences in weight between control, *KRAS^G12D^*, *KRAS^G12D^Ezh2*^ΔSET^, *Mist1^creERT/–^ KRAS^G12D^*, and *MKE* cohorts (Supplemental Figure 8C). However, 3 *MKE* mice needed to be sacrificed prior to the experimental end point. In addition, mice expressing KRAS^G12D^ often developed oral mucosa tumors (data not shown), likely due to *Mist1^creERT^* activity in this tissue, forcing us to cease the experiment at 60 days after initial TX treatment. While most genotypes showed relatively normal pancreatic tissue, *Mist1^creERT/–^ KRAS^G12D^* pancreata contained some fibrotic masses (Supplemental Figure 8D; blue arrows) not observed in *KRAS^G12D^* and *KRAS^G12D^Ezh2*^ΔSET^, consistent with the development of preneoplastic nodules. Pancreatic nodule formation dramatically increased in the absence of EZH2 in *Mist1^creERT/–^ KRAS^G12D^* mice (*MKE*).

**Figure 5:**
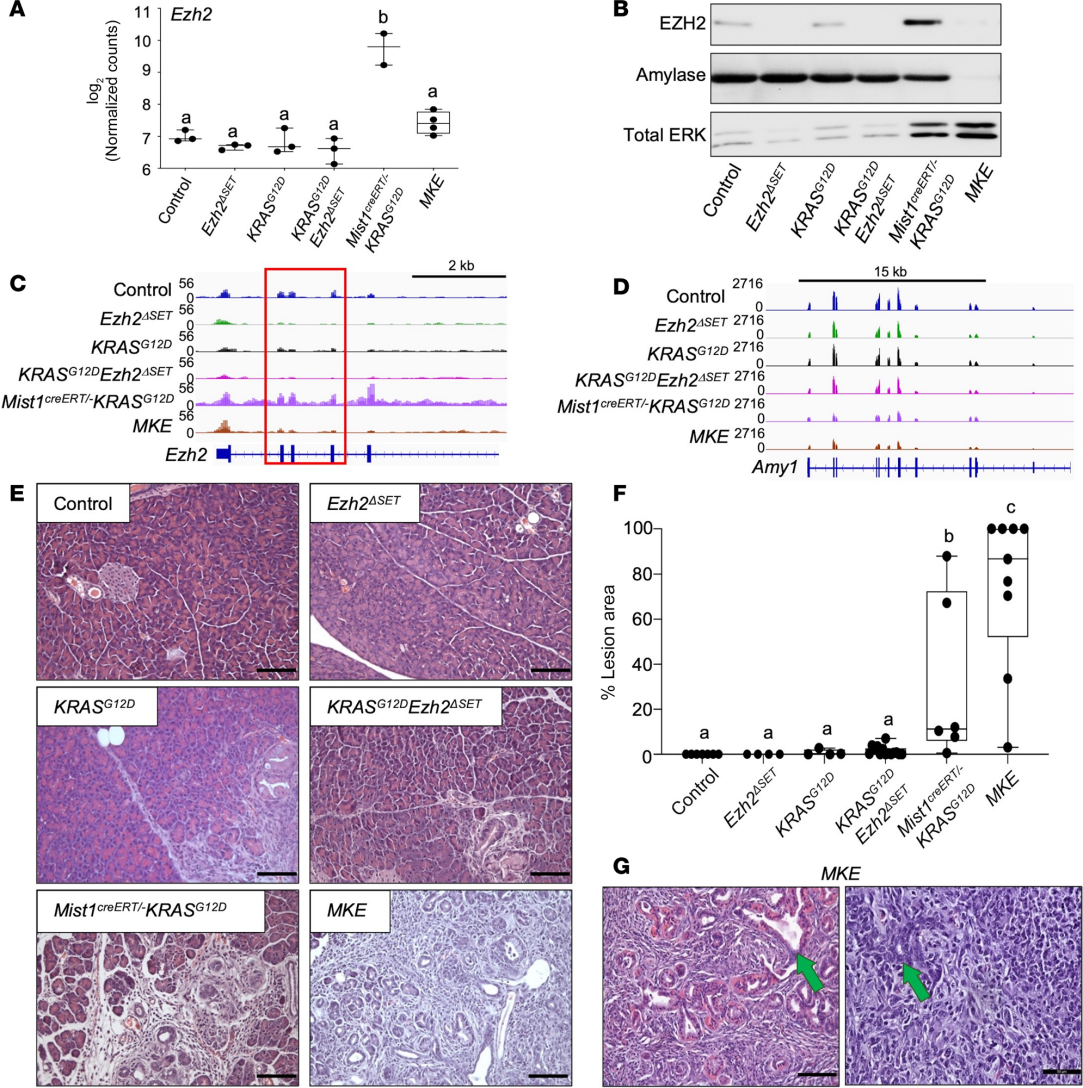
Combined loss of MIST1 and EZH2^ΔSET^ promotes rapid loss of acinar tissue in the presence of *KRAS^G12D^*. (**A**) RNA-Seq analysis revealed marked increases in *Ezh2* in *Mist1^creERT/–^ KRAS^G12D^* pancreatic tissue 22 days after KRAS^G12D^ induction relative to all other genotypes and RNA tracks for *Ezh2* confirm deletion of exon 16–19 (red box). Data represent mean ± minimum to maximum (*n* = 3 for control, *Ezh2*^ΔSET^*, KRAS^G12D^*, *KRAS^G12D^Ezh2*^ΔSET^, and *MKE* and *n* = 2 for *Mist1^creERT/–^ KRAS^G12D^*). Letters indicate statistically similar groups. ^b^*P* ≤ 0.001. (**B**) Representative Western blots for EZH2, amylase, or total ERK, 60 days after KRAS^G12D^ induction. (**C** and **D**) RNA tracks for *Ezh2* (**C**) and *Amy1* (**D**). Tracks are the overlay of *n* = 3 mice. (**E**) Representative H&E-stained pancreatic sections 60 days after KRAS^G12D^ induction. Genotypes are indicated. Scale bar: 100 μm. (**F**) Box plot quantifying the percentage of lesional area in all genotypes based on H&E staining. Data are shown as mean ± minimum to maximum (*n* = 4 for *Ezh2*^ΔSET^ and *KRAS^G12D^*, *n* = 6 for *Mist1^creERT/–^ KRAS^G12D^*, *n* = 7 for control, *n* = 9 for *MKE*, and *n* = 14 for *KRAS^G12D^Ezh2*^ΔSET^). Significance was measured by 1-way ANOVA followed by a Tukey’s post hoc test. Different letters indicate statistically different *P* values; ^b^*P* ≤ 0.01, ^c^*P* ≤ 0.001. (**G**) Higher-magnification images of H&E-stained pancreatic tissue from *MKE* mice. Green arrows indicate high-grade PanIN lesions and putative PDAC that is only found in these animals. Scale bar: 50 μm.

**Figure 6:**
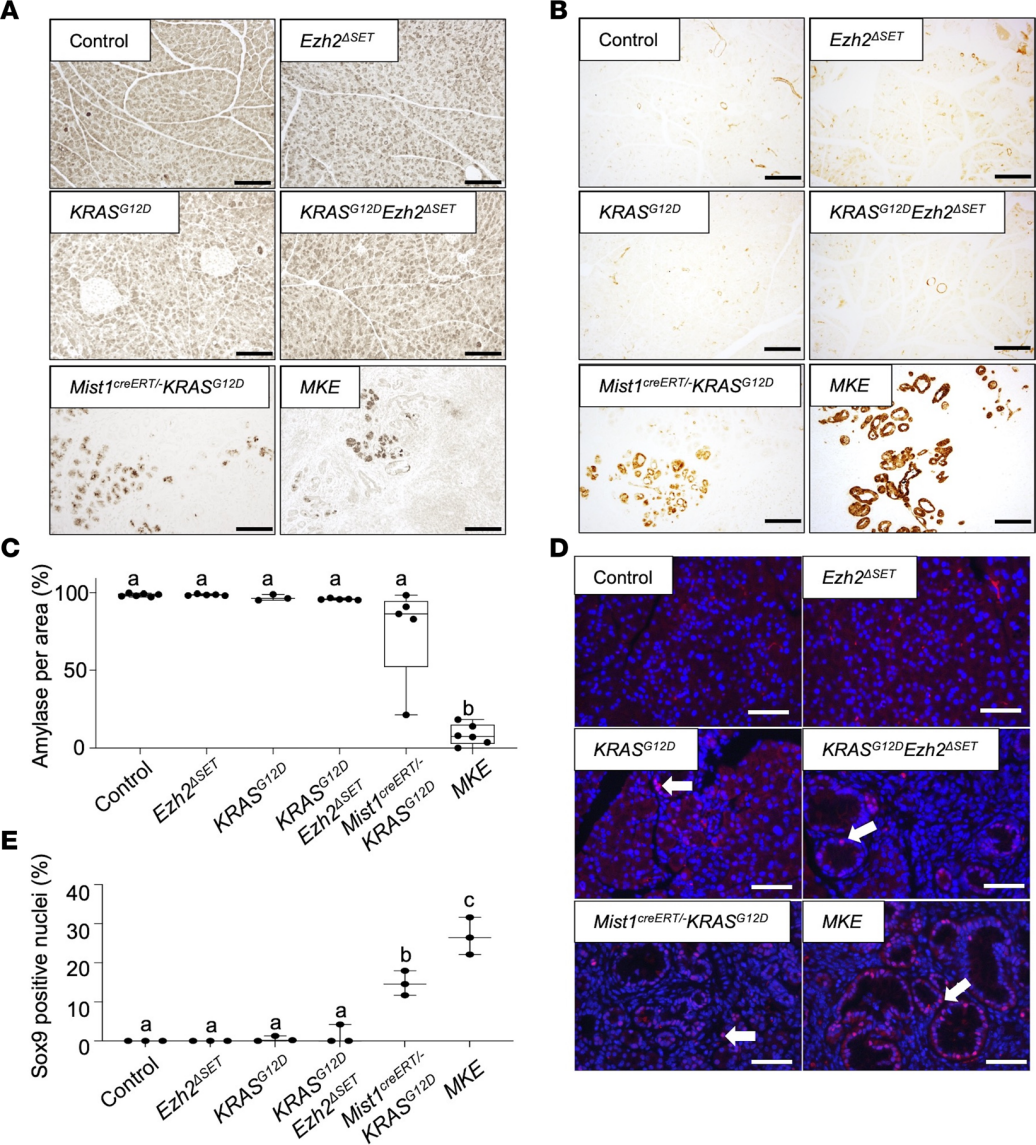
*MKE* mice exhibit extensive ductal and PanIN lesion progression. (**A** and **B**) Representative IHC for amylase (**A**) or CK-19 (**B**) on pancreatic tissue 60 days after KRAS^G12D^ induction. Genotypes are indicated. Scale bar: 100 μm. (**C**) Quantification of amylase staining in the various genotypes based on IHC staining. Data are shown as mean ± minimum to maximum (*n* = 3 mice for *KRAS^G12D^*; *n* = 5 mice for *Ezh2*^ΔSET^*, KRAS^G12D^Ezh2*^ΔSET^, and *Mist1^creERT/–^ KRAS^G12D^*; and *n* = 6 mice for control and *MKE*). Significance was measured by 1-way ANOVA followed by a Tukey’s post hoc test. ^b^*P* ≤ 0.001. (**D**) Representative immunofluorescence for SOX9 on pancreatic sections 60 days after KRAS^G12D^ induction. Genotypes are indicated. Nuclei are counterstained with DAPI. White arrows identity positive SOX9 cells. Scale bar: 50 μm. (**E**) Quantification of SOX9 staining in the different mouse lines based on IF staining. Data are shown as mean ± minimum to maximum (*n* = 3 mice per conditions). Significance was measured by 1-way ANOVA followed by a Tukey’s post hoc test. Different letters indicate statistically different *P* values. ^b^*P* ≤ 0.001, ^c^*P* ≤ 0.0001.

Increased EZH2 accumulation was confirmed in *Mist1^creERT/–^ KRAS^G12D^* pancreatic tissue at the mRNA (Figure 5A) and protein level (Figure 5B) and was lower in *MKE* mice. RNA-Seq showing *Ezh2* tracks confirmed deletion of exon 16–19 (Figure 5C), and amylase protein (Figure 5B) and mRNA (Figure 5D) were decreased in *MKE* tissue, suggesting negligible acinar tissue in these mice. Conversely, ERK levels were elevated in *Mist1^creERT/–^ KRAS^G12D^* and *MKE* extracts (Figure 5B). H&E staining (Figure 5E) and IHC for amylase (Figure 6, A–C) confirmed minimal acinar tissue and development of high-grade PanINs and PDAC in *MKE* mice (Figure 5, E–G). *MKE* pancreata also exhibited widespread fibrosis and invasive PDAC, while *Mist1^creERT/–^ KRAS^G12D^* mice showed some progression to more advanced PanINs (Figure 5E). *KRAS^G12D^* and *KRAS^G12D^Ezh2*^ΔSET^ mice showed few lesions, consisting of ADM and low-grade PanINs. Extensive PanIN lesions in *MKE* mice was confirmed by IHC for CK19 (Figure 6B), and IF (Figure 6, D and E) and RNA-Seq (Supplemental Figure 9A) for SOX9 — a marker of neoplastic lesions — supported markedly increased KRAS^G12D^-mediated PanIN progression to PDAC.

**Figure 7:**
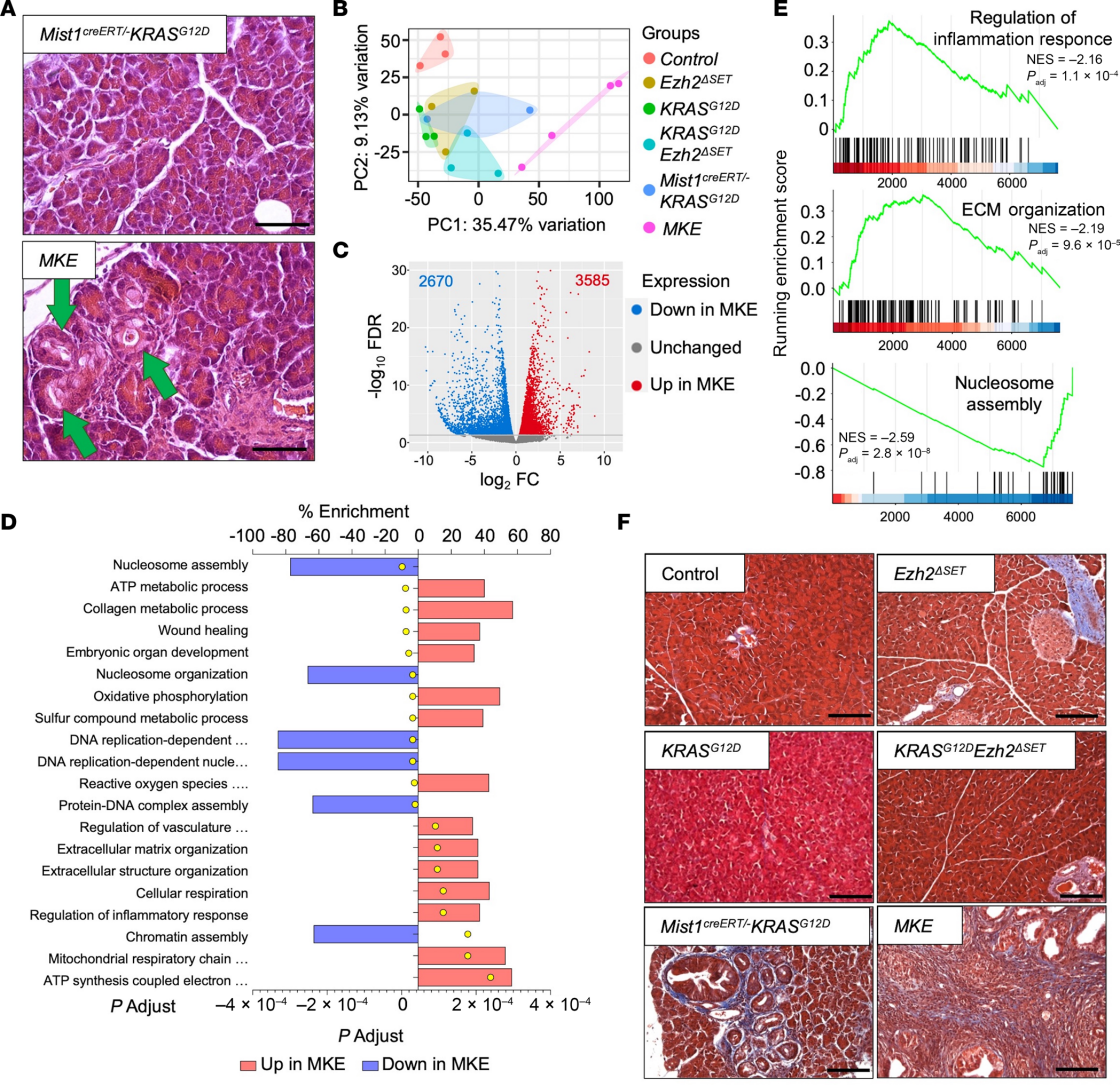
Acinar-specific deletion of *Ezh2*^ΔSET^ in *KRAS^G12D^*-mediated PDAC alters the tumor microenvironment. (**A**) Representative H&E staining of pancreatic tissue from *Mist1^creERT/–^ KRAS^G12D^* and *MKE* mice 22 days after KRAS^G12D^ induction. Green arrows indicate ADM. Scale bar: 50 μm. (**B**) Principal component analysis based on RNA-Seq data 22 days after KRAS^G12D^ induction. (**C**) Volcano plot showing differentially expressed genes between *Mist1^creERT/–^ KRAS^G12D^* and *MKE* mice 22 days after KRAS^G12D^ induction based on RNA-Seq. Genes with significantly lower or higher expression in *MKE* mice are indicated in blue and red, respectively. Significance was determined with a FDR ≤ 0.05. (**D**) Top 20 pathways identified by gene set enrichment analysis using GO terms based on RNA-Seq (*P*_adj._ ≤ 0.05). (**E**) Gene set enrichment analysis shows increased enrichment in KEGG pathways “Regulation of inflammation response” and “ECM organization” in *MKE* tissue compared with *Mist1^creERT/–^ KRAS^G12D^*. Similar analysis shows decreased enrichment of genes involved in “Nucleosome assembly” in *MKE* tissue. (**F**) Representative trichrome blue staining of pancreas section from control, *Ezh2*^ΔSET^, *KRAS^G12D^*, *KRAS^G12D^Ezh2*^ΔSET^, *Mist1^creERT/–^ KRAS^G12D^*, and *MKE* mice 60 days after KRAS^G12D^ induction. Scale bar: 100 μm.

We next integrated RNA-Seq data from *Mist1^creERT/–^ KRAS^G12D^* and *MKE* tissue with the earlier transcriptomic analysis (Figure 3). Twenty-two days after KRAS^G12D^ activation, no lesions were observed in any genotype except in *MKE* tissue, which showed focal ADM (Figure 7A and Supplemental Figure 9B). As expected, *MKE* mice clustered separately from all other genotypes based on transcriptomic analysis (Figure 7B). *Mist1^creERT/–^ KRAS^G12D^* and *MKE* tissue had 7,636 DEGs (Figure 7C and Supplemental Table 9), including many noncoding RNAs (ncRNAs). GSEA using the 6,255 protein-encoding DEGs identified > 150 significantly altered pathways (Supplemental Table 5) including nucleosome and chromatin remodeling, suggesting substantial effects on the acinar cell genome in *MKE* tissue (Figure 7, D and E). Highly enriched pathways in *MKE* mice were also related to TME remodeling and an increased inflammatory response (Figure 7E). *Ptgs2*, which encodes the proinflammatory protein COX2 (Supplemental Figure 9C), was markedly increased only in *MKE* tissue. While negligible fibrosis was evident at the time of transcriptomic analysis (Supplemental Figure 9B), trichrome blue histology showed extensive fibrosis 60 days after treatment in *MKE* pancreata (Figure 7F).

**Figure 8:**
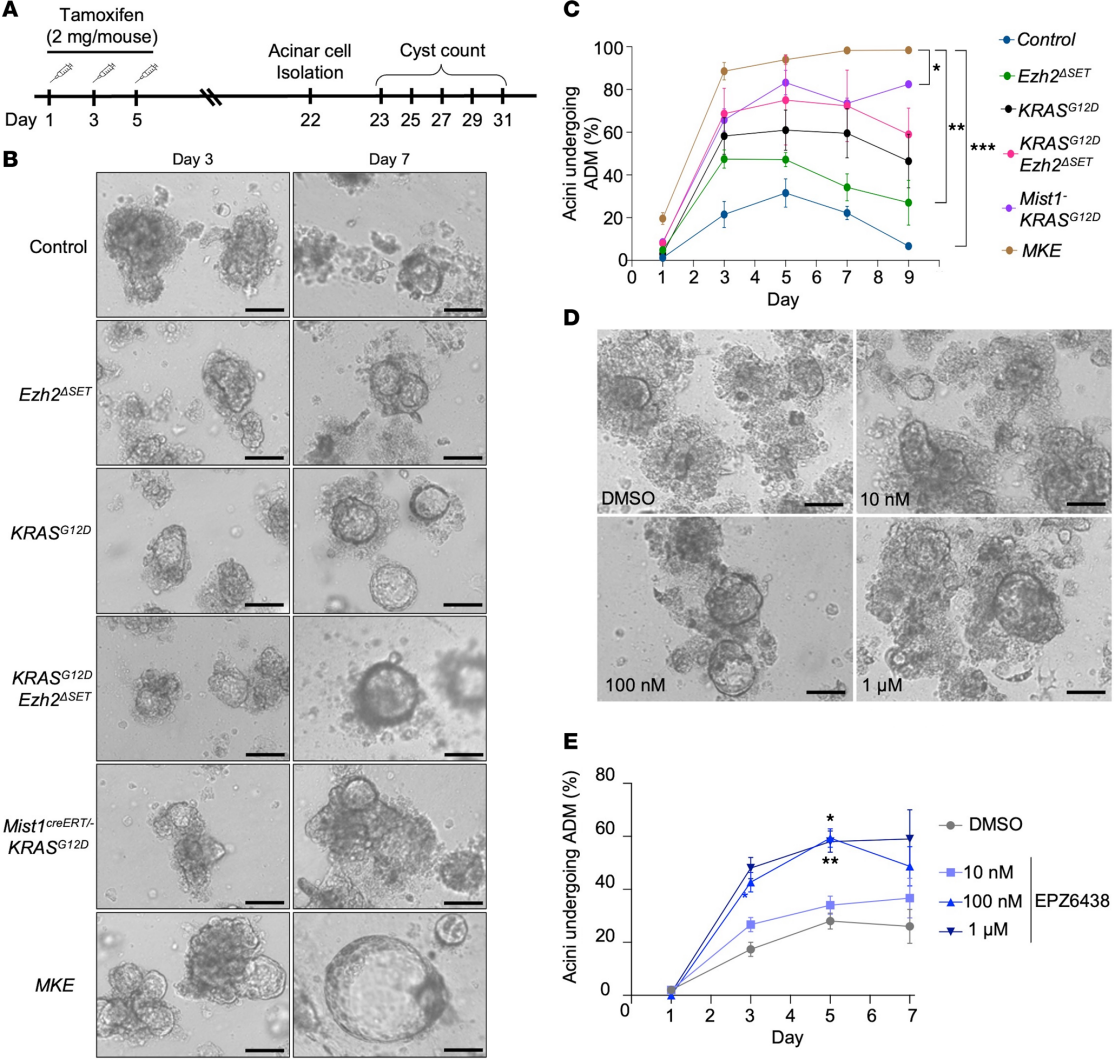
*EZH2*^ΔSET^ deletion increases ADM in the absence of the tissue microenvironment. (**A**) Experimental design for acinar cell isolation and embedding into collagen 22 days after KRAS^G12D^ induction. (**B**) Representative images of cell clusters 3 and 7 days after acinar cell isolation. Genotypes are indicated. Scale bar: 100 μm. (**C**) Quantification of the percentage of cell clusters with visible ADM, 1–9 days after acinar cell isolation. Fifty or more clusters were counted for each condition. Data are shown as mean ± SEM (*n* = 2 for *Mist1^creERT/–^ KRAS^G12D^*, *n* = 3 mice for *KRAS^G12D^Ezh2*^ΔSET^, *n* = 4 mice for *KRAS^G12D^*, *n* = 5 mice for *Ezh2*^ΔSET^, *n* = 6 mice for control, and *n* = 7 mice for *MKE*). (**D**) Representative images of control acinar after 7 days of treatment with increasing amounts of EZH2 inhibitor EPZ6438. Scale bar: 100 μm. (**E**) Quantification of 50+ acinar clusters for each condition. Data are shown as mean ± SEM. *n* = 3. In all cases, significance was measured by a repeated measures 1-way ANOVA followed by Dunnett’s correction. *P ≤ 0.05, **P ≤ 0.01, ***P < 0.001.

These findings reveal EZH2’s effect on acinar cell transformation, but contributions from the ECM and inflammatory responses may contribute to PanIN progression. Therefore, to examine ADM in the absence of the microenvironment, acinar cells were isolated 22 days after activation of KRAS^G12D^ and were cultured in a 3D collagen matrix (Figure 8A). EZH2 recombination was almost complete at this time point (Figure 5B), and transcriptome analysis confirmed that no compensation by *Ezh1* and *Kdm6a/b* occurs in *Ezh2*-deleted cultures (Supplemental Figure 10A). ADM was assessed for 9 days following isolation (Figure 8, B and C). All genotypes showed increased ADM relative to control cultures, with the number of viable ADM decreasing after day 5 except for *Mist1^creERT/–^ KRAS^G12D^* and *MKE* cultures (Figure 8C). *Mist1^creERT/–^ KRAS^G12D^* cultures showed little difference in size from controls, but ADMs were maintained until the end of culture. *MKE* acini developed more rapidly into ADM with ~100% conversion by day 3 and continued to increase in size throughout the culture, showing no obvious apoptosis or necrosis. Staining for Ki67 identified proliferating cells only in *MKE* and *KRAS^G12D^EZH2*^ΔSET^ ADM, consistent with previous reports of EZH2-mediated regulation of cell cycle genes (Supplemental Figure 10B) (12, 32, 33). *p16/CDKN2A*, which affects both senescent and cell cycle pathways, was elevated in *MKE* mice (Supplemental Figure 10C), consistent with EZH2’s role in repressing its expression. Interesting, *p16/CDKN2A* was not altered in *KRAS^G12D^EZH2*^ΔSET^ tissue. Additionally, acinar cultures derived from control mice treated with increasing concentrations of the EZH2 inhibitor EPZ6438 also showed an increase in ADM at 6 days (Figure 8, D and E), consistent with EZH2 restricting initial ADM.

**Figure 9:**
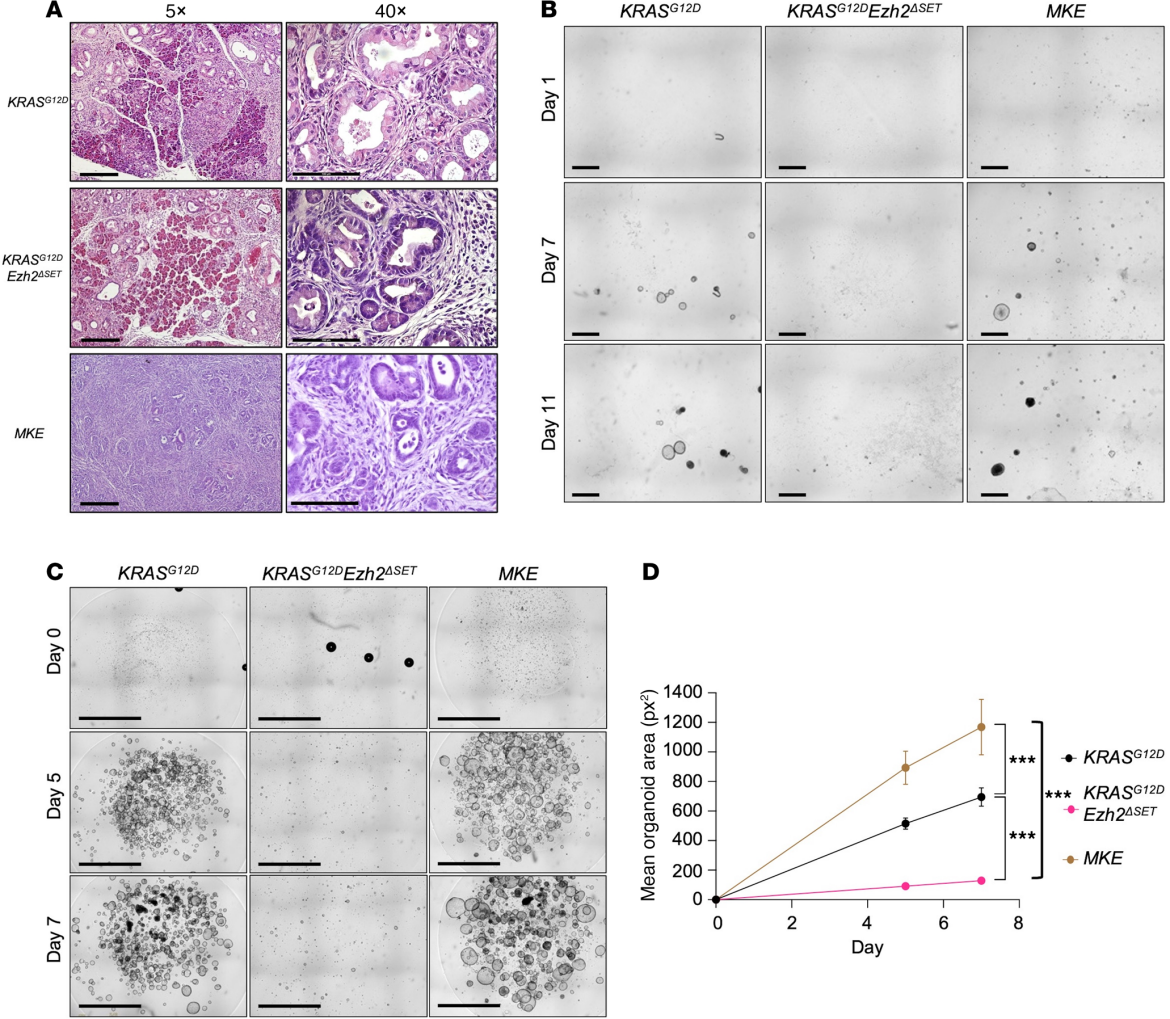
*EZH2*^ΔSET^ deletion has different cell autonomous roles depending on the context in which *KRAS^G12D^* is expressed. (**A**) Morphology of *KRAS^G12D^*, *KRAS^G12D^Ezh2*^ΔSET^, and *MKE* tissue 2 weeks after induction of CIP. Scale bar: 500 μm (left images), 100 μm (right images). (**B**) Representative images of organoids cultured in matrigel 1, 7, and 11 days after isolation. Cells were seeded at 5,000 cells. Genotypes are indicated. Scale bar: 2.4 mm. (**C**) Representative images of organoids 0, 5, and 7 days after first passage. Cells were seeded at 5,000 cells. Scale bar: 2.4 mm. (**D**) Quantification of organoid area 5 and 7 days after passage for *KRAS^G12D^*, *KRAS^G12D^Ezh2*^ΔSET^, and *MKE* cultures. Data represent mean ± 95% CI. Number of organoids assessed is indicated above each data point. Significance was measured by 2-way ANOVA followed by Tukey’s correction. ***P ≤ 0.001.

To determine if the absence of EZH2 affects the maintenance of epithelial neoplasias, we developed 3D organoid cultures from *KRAS^G12D^*, *KRAS^G12D^Ezh2*^ΔSET^, and *MKE* pancreatic tissue 2 weeks after cerulein induction (Supplemental Figure 11, A and B), when PanINs have developed (Figure 9A). Organoids were readily observed in *KRAS^G12D^* cultures, but *KRAS^G12D^Ezh2*^ΔSET^ cultures showed few, smaller organoids (Figure 9B), a difference maintained upon passaging (Figure 9C). While organoids from *MKE* tissue initially appeared similar to cultures developed from *KRAS^G12D^* tissue (Figure 9B), after only 1 passage, *MKE* organoids showed rapid growth and larger organoid structures compared with *KRAS^G12D^* or *KRAS^G12D^Ezh2*^ΔSET^ cultures (Figure 9, C and D). To show an ongoing requirement for EZH2 in neoplastic cells, *Mist1^creERT/–^ KRAS^G12D^* organoids were developed from CIP-treated mice and exposed to EPZ6438 for 7 days, and growth was compared with *MKE* organoids. At the time of dissection, *Mist1^creERT/–^ KRAS^G12D^* tissue showed extensive ADM but maintained the lobular nature of the pancreas and did not show the same extent of fibrosis as that observed in *MKE* tissue (Supplemental Figure 11C). EZH2 inhibition markedly increased the size of *Mist1^creERT/–^ KRAS^G12D^* organoids, becoming similar in size to *MKE* organoids (Supplemental Figure 11, D–F). These 3D cultures highlight that cell autonomous events are at least partially responsible for the *MKE* phenotype and increased progression to PDAC observed in *MKE* mice and support a contextual role for EZH2 in early PDAC progression.

## Discussion

In this study, the effect of KRAS^G12D^ and loss of EZH2 on epigenetic remodeling, neoplastic lesion development, and progression was examined. Using a model allowing inducible activation of KRAS^G12D^ in acinar cells of adult mice, we showed that KRAS^G12D^ promotes epigenetic reprogramming of the acinar cell genome, leading to widespread EZH2-dependent K27me3 enrichment. EZH2 is dispensable for acinar cell regeneration following pancreatic injury but restricts PanIN progression following acute injury combined with *KRAS^G12D^*. While this difference did not result in high-grade PanIN lesions, loss of EZH2^ΔSET^ activity greatly enhanced PDAC progression in mice *Mist1^creERT/–^ KRAS^G12D^*, leading to spontaneous loss of acinar tissue, substantial fibrosis, and PDAC within 60 days. This is the first study to our knowledge that examines changes in acinar cell K27me3 enrichment profiles directly related to KRAS^G12D^ expression, how these changes are affected by EZH2 function, and context-specific roles for EZH2 that promote or restrict early PanIN progression. This study also highlights the importance of epigenetic reprogramming in the context of PDAC and suggests that EZH2 restricts early PanIN progression to PDAC through priming of immune and inflammatory genes.

### KRAS^G12D^ promotes epigenetic repression of the acinar cell genome.

Our findings support a model in which KRAS^G12D^ promotes general epigenetic repression within the pancreas prior to overt morphological changes. Global enrichment of K27me3 was increased in *KRAS^G12D^* compared with control tissue, while global K4me3 enrichment was similar between KRAS^G12D^ and tissue. This is consistent with studies showing increased expression and activity of DNA methyltransferases, histone deacetylases, and PRC1 and PRC2 in PDAC (34–37), all of which promote epigenetic repression. Importantly, epigenetic reprogramming does not accompany widespread transcriptomic dysregulation, suggesting that changes in the epigenome predate transcriptional differences and may be masked until additional environmental stresses are present. We previously characterized similar epigenetic reprogramming of acinar cells in response to chronic stress, which suggested that reprogramming alters the molecular response to subsequent acute stimuli (38). One mechanism that underlies reprogramming involved changes to “primed” genes, which have bivalent epigenetic enrichment for active and repressive epigenetic marks. This epigenetic bivalency allows repressed genes to be rapidly activated and involves K27me3 enrichment. The widespread enrichment of K27me3 following KRAS^G12D^ activation suggests EZH2, in part, regulates reprogramming. In support of these findings, deletion of EZH2 in the presence of KRAS^G12D^ resulted in K27me3 enrichment levels comparable with control tissue.

### Loss of EZH2 leads to epigenetic reprogramming of pathways involved inflammation.

While EZH2 has been targeted in several other studies examining its role in PDAC (15, 39, 40), this is the first study to our knowledge that examines global K27me3 enrichment in the context of EZH2 loss of function. K27me3 ChIP-Seq combined with RNA-Seq revealed increased enrichment of immune-related pathways 22 days after KRAS^G12D^ induction that appears to prime the genome for a differential inflammatory response since no immune cell infiltration was observed in the pancreas until after induction of injury. Five weeks after pancreatic injury, *KRAS^G12D^* mice showed an increase immune cell infiltration such as CD3^+^, CD4^+^, CD8^+^ lymphocytes, and F4/80^+^ macrophages cells. The accumulation of each of these cell types was reduced in the absence of EZH2^ΔSET^ activity, suggesting loss of EZH2 activity in *KRAS^G12D^Ezh2*^ΔSET^ mice drives an immune cold environment. Decreased accumulation of CD4^+^ and CD8^+^ cells would promote PDAC progression, as their presence is associated with improved prognosis of patients with PDAC (41–43). These findings support recent studies that propose a direct role for EZH2 in immune cell recruitment and activation in cancer (44, 45) and suggest that EZH2 plays a protective role in early PanIN development by increasing specific CD45^+^ immune cell infiltration such as CD4^+^ and CD8^+^ cell recruitment. However, the absence of EZH2 activity also results in decreased accumulation of F4/80^+^ macrophages cells, which are generally associated with enhanced PDAC progression since these favor the immunosuppressive environment (46, 47). The contradiction supports a more complex involvement of EZH2 that is likely stage dependent. This is supported by analysis of organoids developed from *KRAS^G12D^Ezh2*^ΔSET^ and *KRAS^G12D^* pancreatic tissue, which showed no difference in vivo but exhibited marked differences ex vivo. Organoids developed from *KRAS^G12D^Ezh2*^ΔSET^ tissue had reduced size and number, suggesting Ezh2^–^ lesions have a reduced ability for long-term progression. The different outcomes between in vivo and ex vivo following altered *Ezh2* function could suggest a non–cell intrinsic role for EZH2 in affecting the tissue microenvironment, and this is supported by the differences in immune cell infiltrate. However, it is also possible that early advantages gained by the loss of EZH2 in promoting PanIN differentiation are lost as PanINs progress to a more advanced phenotype. This phenomenon is consistent with observations in Chen et al. (14), which showed the increased PanIN progression initially observed in the absence of EZH2 was not maintained at later stages. However, the disadvantage of not having EZH2 as PanINs progress appears to be bypassed by the loss of MIST1. Whether this is still due to external differences within the microenvironment will need to be assessed.

### Loss of EZH2^ΔSET^ activity enhances a susceptible environment for KRAS^G12D^-mediated PDAC.

As mentioned, while our findings suggest a protective role for EZH2 in limiting early PanIN progression, previous studies on EZH2 show a more critical role in early stages of PDAC. Using the same floxed *EZH2*^ΔSET^ allele, Mallen-St Clair et al. (12) showed that acinar cell regeneration is restricted following cerulein-induced injury and increases KRAS^G12D^-mediated PanIN initiation and progression, consistent with a restrictive role for EZH2 methyltransferase function (12, 14). As mentioned above, Chen et al. (14) supported these findings but that suggested EZH2 was necessary for maintaining preneoplastic lesions, with fewer PanIN lesions apparent in older mice. Our results reveal negligible effects on acinar cell regeneration as *KRAS^G12D^* and *KRAS^G12D^Ezh2*^ΔSET^ mice developed similar numbers of PanINs lesions following injury. We suggest the discrepancy in our results arises, in part, from the *Cre* driver used in the 2 studies having different effects on susceptibility to KRAS^G12D^. Previous studies achieved *Ezh2*^ΔSET^ deletion by targeting a noninducible *Cre* recombinase to the *Ptf1a* or *Pdx1* genes resulting in *KRAS^G12D^* activation in early pancreatic development, prior to differentiation of mature pancreatic cell types. EZH2 is important for early development and specification (8) of acinar and liver cells from a common endodermal origin. In the absence of EZH2, epigenetic programs that fix in the differentiation status of mature cell types are absent. *Mist1^creERT^* mice allow *Ezh2*^ΔSET^ deletion and *KRAS^G12D^* activation only in mature acinar cells when mature epigenetic programs are already in place. Therefore, epigenetic programs that establish an adult phenotype are not affected. In addition, haploinsufficiency for *Ptf1a* likely affects the response to KRAS^G12D^. Loss of a single *Ptf1a* allele alters the cell fate of acinar cells (48), which increases the potential for undergoing ADM. Conversely, loss of a single *Mist1* allele shows no differences in acinar cell function, response to injury, or gene expression when compared with WT litter mates. Only when MIST1 is completely absent do acinar cells show incomplete differentiation and increased sensitivity to injury and KRAS^G12D^ (31, 49). In support of the importance of the Cre driver for studying PanIN progression, comparison of *Ptf1a^creERT/+^ KRAS^G12D^* mice to *Mist1^creERT/+^ KRAS^G12D^* or *Elastase^creERT/+^ KRAS^G12D^* mice showed marked differences in sensitivity to cerulein-induced injury (50).

### Loss of EZH2 methyltransferase activity leads to both cell autonomous and non–cell autonomous effects on PDAC progression.

Despite the differences, both the current study and Chen et al. (14) confirm a protective role for EZH2 in restricting early PanIN progression and PDAC development. We suggest that this effect of EZH2 is through both cell autonomous and non–cell autonomous effects within the pancreas. RNA-Seq at 22 days revealed that loss of EZH2 activity in *Mist1^creERT/–^ KRAS^G12D^* mice (i.e., *MKE*) leads to activation of pathways involved in TME remodeling that favor aggressive PDAC progression (51–53), and the rapid progression to ADM and PanINs appears to be independent of the TME. RNA-Seq analysis also revealed that loss of EZH2 had a substantial effect on pathways affecting chromatin stability in *MKE* acini, suggesting a cell autonomous role for EZH2 in acinar cell reprogramming. This role was confirmed by culturing acinar cells of all genotypes in collagen 22 days after TX-induced recombination or culturing organoids from *KRAS^G12D^* and *MKE* genotypes following acute cerulein treatment. In collagen cultures, *MKE* acini showed rapid ADM compared with other genotypes, with increased proliferation, and maintained survival over the length of culture. In matrigel cultures, *MKE* organoids show rapid growth and formed and maintained larger cyst structures compared with the *KRAS^G12D^* cultures. Interestingly, inhibition of EZH2 both in control acinar cells and *Mist1^creERT/–^ KRAS^G12D^* organoids induces and increase of ADM formation and organoids size, respectively.

Targeting EZH2 function has been suggested as a possible therapy based on in vitro and xenograft data showing EZH2 inhibitors can enhance sensitivity to traditional chemotherapy (54). While our results support and extend findings of the importance of EZH2 in restricting progression to PDAC, they are not in agreement with studies on PDAC cell lines or tissue obtained from patients. Increased EZH2 expression in PDAC is correlated to worse prognosis and resistance to therapy (13, 15, 39). Crucially, these previous studies suggest that the effects of EZH2 are independent of its methyltransferase activity. Therefore, it is likely that EZH2 has additional, non-PRC2 functions relevant to late-stage PDAC, which our study does not address. However, our findings suggest targeting EZH2 in PDAC with pharmacological inhibitors must be approached with caution.

While this is the first study to our knowledge to identify specific EZH2 roles on epigenetic reprogramming following induction of KRAS^G12D^, there are limitations to the work. H3K4 and H3K27 are only 2 epigenetic modifications linked to gene expression, and other modifications are more consistent with gene expression. K36me3 and K9me3 enrichment are more closely correlated with gene expression and repression, respectively, and DNA methylation is highly correlated with gene repression. While we have focused on K4me3 and K27me3 due to their roles in epigenetic bivalency, a more comprehensive analysis is warranted. Similarly, while *Mist1^creERT^* driver mice provide a more relevant model of PDAC compared with previous studies using *Ptf1a-Cre* mice, which activates KRAS^G12D^ in development, an inducible *Ptf1a^creERT^* model is available that would allow longer-term analysis given its pancreas-specific expression. However, this model shows increased sensitivity to KRAS^G12D^ that may not be physiologically relevant (50).

To conclude, our study shows that EZH2 limits progression from acinar cells to late-stage PDAC through reprogramming of inflammatory and extracellular matrix genes. These effects are likely through both non–cell autonomous and cell autonomous mechanisms. Loss of EZH2 alters pathways that promote inflammation and fibrosis, thereby affecting the TME, but it also enhances ADM in the absence of the TME. This work highlights a complex role for EZH2 in initiation and progression of pancreatic cancer. While our findings support a tumor-suppressive role in restricting PanIN and PDAC formation, future studies are needed to determine if these effects are simply due to PRC2-related functions or additional modes of EZH2 activity.

## Methods

### Sex as a biological variable.

In this study, both male and female C57BL/6 mice were used for each genotype. Sex was not considered as a biological variable in this study.

### Mouse models.

Our study examined male and female animals, and similar findings are reported for both sexes. In all experiments, both male and female mice were used to reach significance. Mice were given normal chow and water ad libitum throughout the experiment. C57BL/6 mice containing *loxP* sites flanking exons 16–19 of the *Ezh2* gene (encompass the SET domain; *Ezh2*^Δ*SET/*ΔSET^), an oncogenic KRAS^G12D^ within the *Kras* locus and downstream of a *loxP-stop-loxP* (*LSL*) cassette (*Kras^LSL-G12D^*), or an inducible Cre recombinase (creERT) targeted to the *Mist1* coding region (*Mist1^creERT^*), have been used and described previously (12, 18, 25, 55, 56). Mating of these transgenic lines led to 8 distinct genotypes, which were confirmed before and after experimentation using the primers indicated in Supplemental Table 6. To induce *loxP* recombination, 2- to 4-month-old mice were gavaged 3 times over 5 days with 2 mg TX (MilliporeSigma, T5648) in corn oil (Sigma, C8267). This regime has been used previously to induce > 95% recombination in acinar cells of the *Mist1^creERT^* line (31, 57). Mice were sacrificed either 22 days or 60 days after the initial TX gavage or treated with cerulein to induce acute or recurrent pancreatic injury (see below). Pancreatic tissue was weighed and processed for paraffin sectioning, RNA, chromatin, or protein isolation.

### CIP.

To induce acute pancreatic injury, 2- to 4-month-old mice received 8 hourly i.p. injections of cerulein (50 mg/kg, MedChemExpress, FI-6934) 15 and 17 days after the first dose of TX. Control mice received 0.9% saline solution. Mice were weighed every day to monitor weight changes and health; they were then sacrificed 14 or 35 days after initiating acute CIP.

To induce recurrent pancreatic injury, mice received i.p. injections of cerulein (250 μg/kg body weight) or 0.9% saline solution (control) twice daily (9:00 hours and 15:00 hours) for 14 days. Mice were weighed daily to determine changes in body weight. Mice were sacrificed 7 days after the last cerulein injections.

### RNA isolation, RNA-Seq, and data analysis.

RNA was isolated from whole pancreatic tissue of mice 22 days after TX induction using Trizol (Invitrogen, 15596018) followed by the Pure link kit following manufacturer’s instructions (Invitrogen, 12183018A). RNA was prepared for RNA-Seq as previously described (33). Two (for *Mist1^creERT/–^ KRAS^G12D^*) or 3 (Control, *EZH2*^ΔSET^, *KRAS^G12D^*, *KRAS^G12D^EZH2*^ΔSET^, and *MKE*) biological replicates per group were sequenced using the Illumina NextSeq High Output 150 cycle (paired-end sequencing) sequencing kits. The complete RNA-Seq data can be found at GEO accession GSE (GSE262920 and GSE252884). RNA-Seq reads were aligned to mouse genome mm10 and sorted by coordinate using STAR v2.7.9a (58). Gene counts were generated using the featureCounts function of the Subread v2.0.3 aligner (59), and the subsequent differential expression analysis was performed using the edgeR v3.321 package (60, 61). The DEGs acquired from this analysis were used in subsequent functional analysis and later in the comparison with genes obtained from ChIP-Seq analysis. Functional and enrichment analysis, including KEGG and gene ontology (GO) pathway analyses and GSEA, were performed using clusterProfiler v3.18.1 R package (62). A threshold of *P*_adj._ ≤ 0.05 cut off was used for all differential expression and pathway analyses. PCA plots v2.2.0 (DOI: 10.18129/B9.bioc.PCAtools), Venn diagrams v1.7.3 (63), and dot plots v1.10.2 (64) were generated using the corresponding R package.

### ChIP-Seq and data analysis.

Chromatin was isolated from pancreatic tissue of mice 22 days after TX gavage. The ChIP-Seq protocol was followed as previously described (38). Antibodies against K27me3 (MilliporeSigma, 07-449) or K4me3 (MilliporeSigma, 04-745) were used for immunoprecipitation, and subsequent next-generation sequencing was performed using Illumina NextSeq High Output 150 cycle sequencing kit. The complete ChIP-Seq data can be found at GEO accession GSE (GSE262919). Raw data were first checked for read quality using FastQC and aligner against the mouse genome (mm10) using bowtie2 tool (65). Identification of the peaks for each sample was performed using Homer FindPeaks tool with the “histone” mode, which searches for broad regions of enrichment of variable width by comparing both local background and corresponding input samples. Genomic annotation and visualization of the peaks was performed using ChIPSeeker R package and *TxDb.Mmusculus.UCSC.mm10.knownGene* library. To define the target genes with marked ChIP enrichment, we defined the promoter region of ± 3 kb from the TSS. Genes overlapping at least 1 identified peak were considered target genes for a given sample. KEGG enrichment analysis was performed based on the resulting lists of target genes using ClusterProfiler R package. Heatmap visualization of the ChIP enrichment was performed using ngs.plot tool (66) with decreasing ranking of genes based on the ChIP enrichment level among the gene body. Browser Extensible Data (BED) files with K4me3- and K27me3-aligned reads and their corresponding input samples were used to assess chromatin states with ChromHMM (67). The resulting output generated 4 chromatin states. The enrichment of each state was calculated and visualized, and the states were annotated based on the enrichment patterns.

### qPCR analysis.

quantitative PCR (qPCR) was performed on cDNA samples prepared as described (38). Expression of *Ptgs2* was normalized to mitochondrial ribosomal protein L1 (*Mrpl1*). ViiA 7 RUO software (Applied Biosystems) was used to calculate the amount of RNA relative to WT animals for the equivalent time points. Primer sequences are shown in Supplemental Table 7.

### Tissue fixation and histology.

For histological analysis, pancreatic tissue was isolated from the head and tail of the pancreas and processed as described (38). To assess overall histology and identify differences in pancreatic tissue architecture, sections were stained with H&E. Lesions area were quantified using ImageJ (NIH) as a percentage of total tissue area. Mucin accumulation was visualized using an Alcian Blue stain kit (Abcam, ab150662), and staining was quantified as a percentage of the whole tissue area. PAS staining was also performed (Sigma-Aldrich, 3951 and 3952) and quantified by scoring PanIN lesions as PAS^+^ (>50%), partially PAS^+^ (<50%), or PAS^–^. To assess fibrosis, paraffin sections were stained using Trichrome Blue (Abcam, ab150686). Lesions and other staining were scored over at least 3 sections from both the duodenal and splenic regions of the pancreas.

### IHC and immunofluorescence.

IHC was performed on paraffin sections as described (38). Following antigen retrieval, sections were permeabilized with 0.2% Triton-X (BDH, R06433) in PBS, rinsed, and blocked in 5% sheep serum in PBS for 1 hour at room temperature. Primary antibodies were diluted in 5% sheep serum in PBS and incubated overnight at 4°C. Primary antibodies included rabbit amylase (Cell Signaling Technology, 4017, 1:400), rabbit CK19 (Abcam, 15463, 1:200), rabbit CD3 (BD Biosciences, 560591, 1:200), rabbit CD8 (Thermo Fisher Sciences, 98941, 1:200), rabbit F4/80 (Abcam, ab111101, 1:100), rabbit α-SMA (Cell Signaling Technology, 19245, 1:200), and rabbit Vimentin (Cell Signaling Technology, 5741, 1:400). Sections were washed and then incubated in biotinylated mouse α–rabbit IgG secondary antibody (in 5% sheep serum, Vector, PK-4001, 1:1000) for 30 minutes at room temperature. Finally, sections were incubated in AB reagent for 30 minutes at room temperature and visualized using ImmPACT DAB Peroxidase (HRP) substrate (Vector, PK-4001/SK-4105). Slides were counterstained with hematoxylin (Biocare Medical, CATHE-M) and imaged using Leica Microscope DM5500B (Leica Microsystems) and LAS V4.4 software.

IF analysis was performed on paraffin-embedded tissue sections for SOX9 and CD4; for Ki67, acinar cells were fixed in PFA 3% and then embedded in paraffin. Slides were prepared as for IHC except for quenching with hydrogen peroxidase (Thermo Fisher Scientific, H325) for SOX9. Primary antibody is rabbit SOX9 (MilliporeSigma, AB5535, 1:250), rat CD4 (Thermo Fisher Sciences, 14-0041-82, 1:250), and mouse Ki67 (BD Biosciences, 550609, 1:250). After washing, slides were incubated in α-rabbit or α-mouse IgG conjugated to TRITC (Jackson ImmunoResearch, 711-025-152 and 715-025-150, 1:300) or α–rat IgG conjugated to FITC (for CD4) (Jackson ImmunoResearch, 712-095-150) diluted in 5% sheep serum in PBS. Prior to mounting in Vectashield Permafluor mountant (Thermo Fisher Scientific, SP15), sections were incubated in DAPI (Thermo Fisher Scientific, 62248). Staining was visualized using Leica DFC365 FX camera on the Leica DM5500B microscope. Images were taken on Leica LASV4.4 software.

### Protein isolation and Western blotting.

Pancreatic protein was isolated as described (68) and quantified using a Bradford protein assay (Bio-Rad, 5000006). Isolated protein was resolved by SDS-PAGE and transferred to polyvinylidene fluoride membrane (Bio-Rad, 162-0177). Western blot analysis was carried out as described (69) using antibodies specific for rabbit EZH2 (Cell Signaling Technology, 5246, 1:1,000), rabbit Amylase (Abcam, ab21156, 1:8,000), and rabbit total ERK (Cell Signaling Technology, 9102, 1:1,000). After washing, blots were incubated in α–rabbit HRP antibody (Cell Signaling Technology, 7074, 1:3,000). Blots were visualized using the VersaDoc Imaging System with Quantity One 1-D Analysis software (Bio-Rad).

### Acinar cell isolation and 3D collagen culture.

Acinar cells were isolated and embedded in collagen as previously described (70). Cyst formation was assessed every day until day 9 in culture. At day 7, some cultures were processed for paraffin sectioning and IF analysis for Ki67. Representative images were taken with an upright Leica microscope.

### Organoid isolation and 3D matrigel culture.

The middle section of the pancreas was isolated and digested based on previously published protocols with some modifications (71). Pancreata was digested by incubation in 1 mg/mL of collagenase/dispase for 20 minutes at 37°C in a rotating incubator. Digested tissue was washed with DMEM/F12 (Wisent, 390-075CL) containing with 10 mM HEPES, 1% glutamax (Thermo Fisher Scientific, 35050061), 1% penicillin-streptomycin (PenStrep), and 100 μg/mL primocin (Invitrogen, anti–pm-1) and centrifuged at 300*g* for 5 minutes. Supernatant was aspirated and tissue resuspended in StemPro Accutase (Thermo Fisher Scientific, A11105-01) and incubated for 45 minutes at 37°C in a rotating incubator. The resulting slurry was filtered through a 70 μm nylon mesh filter and cells resuspended in feeding media (72) with 5% Matrigel. In total, 30,000 cells were seeded on a layer of 100% Matrigel (Corning, 356230). After first passage, organoids were reseeded into 100% Matrigel domes for experimental analysis according to ref. 73. For passaging, organoids were incubated in 1 mg/mL of collagenase/dispase for 2 hours at 37°C and were then rinsed with wash media and centrifuged at 300*g* for 5 minutes. Supernatants were aspirated, and cells were resuspended in StemPro Accutase and incubated for 45 minutes at 37°C in a rotating incubator. Cells were centrifuged at 300*g* for 5 minutes and supernatant aspirated. In total, 5,000 cells were reseeded at equal densities in 100% Matrigel and supplemented with feeding media.

### Statistics.

For ADM 3D culture quantification, we used 2-way repeated ANOVA followed by Dunnett’s correction. For organoid quantification, we used a 2-way ANOVA followed by Tukey’s correction. For in vivo experiment, when 2 conditions were compared, a 2-tailed unpaired Mann-Whitney *U* test was used. For more than 2 conditions comparison, 1-way ANOVA followed by Tukey’s correction were performed. *P* ≤ 0.05 or adjusted *P* (*P*_adj._) ≤ 0.05 were considered significant for all our analysis.

### Study approval.

All experiments on mice were approved by the Animal Care Committee at the University of Western Ontario (protocol nos. 2020-057 and 2020-058).

### Data availability.

We have uploaded data to NCBI. The complete RNA-Seq data can be found at GEO accession GSE (GSE262920 and GSE252884) and the complete ChIP-Seq data can be found at GEO accession GSE (GSE262919). Values for all data points in graphs are reported in the Supporting Data Values file.

## Author contributions

EJP contributed data acquisition and interpretation as well as manuscript writing and editing; XW contributed data acquisition and interpretation as well as manuscript writing; FM contributed data acquisition and interpretation as well as manuscript writing and editing; ZK contributed data acquisition; SE contributed data acquisition and interpretation; KB contributed data acquisition; CJ contributed data acquisition and interpretation; MBM contributed data acquisition; SA contributed data acquisition; SB contributed data acquisition; KM contributed data acquisition; JR contributed data acquisition; PS contributed data interpretation and mentorship; AJM contributed data acquisition and interpretation as well as manuscript editing; ND contributed data interpretation and mentorship; RU contributed data interpretation and mentorship; GL contributed data interpretation and mentorship; and CLP contributed study design, data interpretation, manuscript writing and editing, and mentorship.

## Supplementary Material

Supplemental data

Unedited blot and gel images

Supplemental table 8

Supplemental table 9

Supporting data values

## Figures and Tables

**Table 1:**
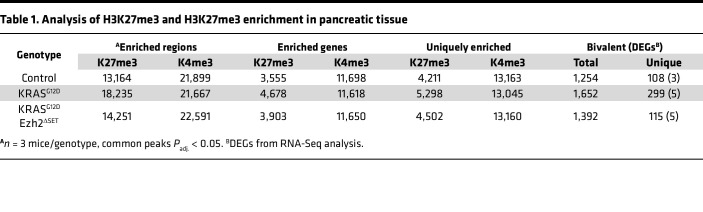
Analysis of H3K27me3 and H3K27me3 enrichment in pancreatic tissue

## Version 1 12/31/2024

In-Press Preview

## Version 2 02/10/2025

Electronic publication
