# Supplementary material for: EZH2 deletion does not affect acinar regeneration but restricts progression to pancreatic cancer in mice
Source: JCI Insight. 2024 Dec 31;10(3):e173746. doi: 10.1172/jci.insight.173746 (PMC11948588; doi:10.1172/jci.insight.173746)
Supplement: Supplemental data [file jciinsight-10-173746-s132.pdf]

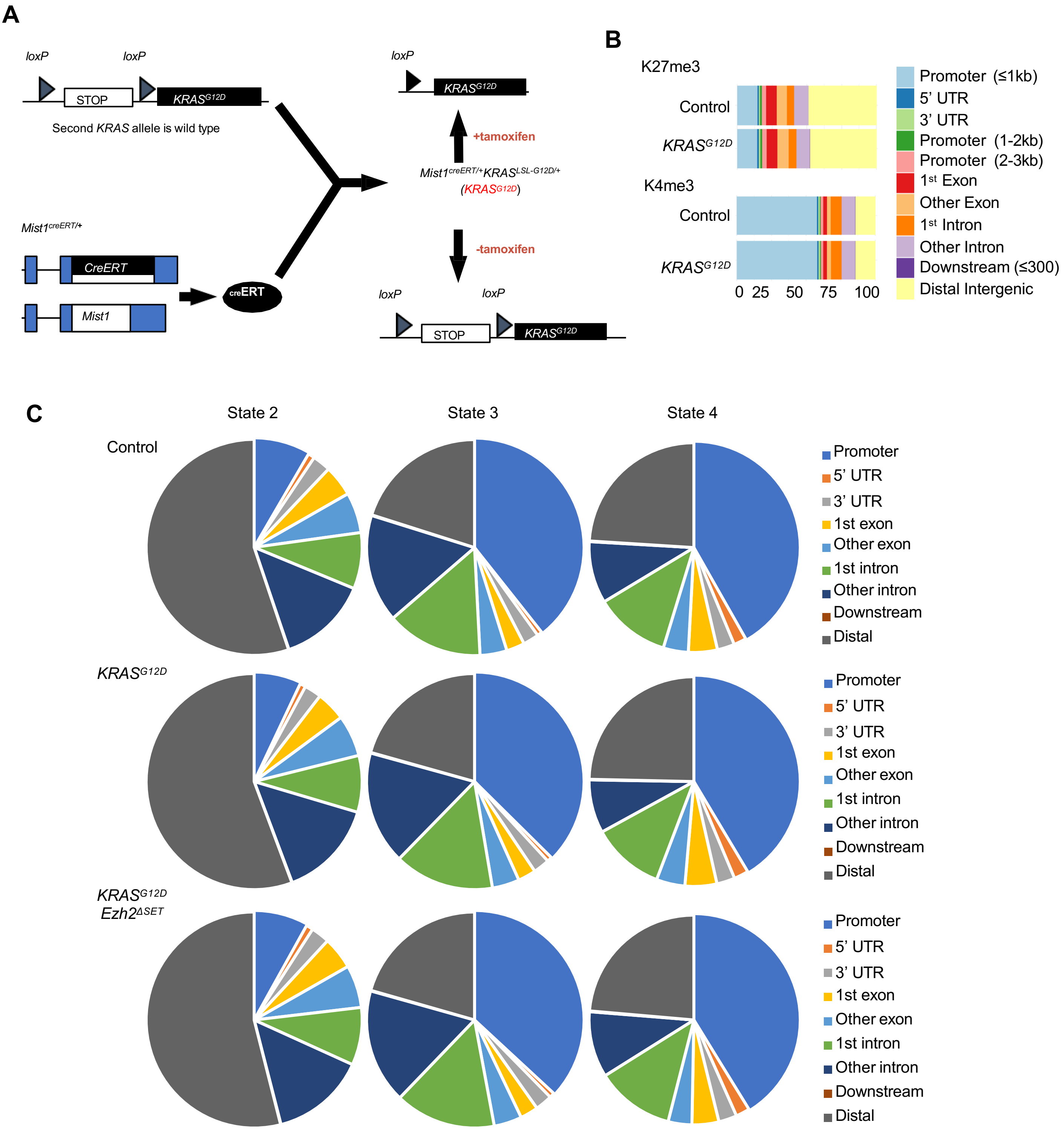

Figure S1. Analysis of transcriptomic changes in response to *KRAS^{G12D}* activation in pancreatic acinar cells. **(A)** Schematic representation of *KRAS^{G12D}* induction by tamoxifen (TX) gavage specifically in acinar cell of mice driven by the *Mist1$^{creERT/+}$* promoter. **(B)** Genome distribution of K4me3 and K27me3 enrichment in control and *KRAS^{G12D}* pancreatic tissue. **(C)** Genome enrichment for K27me3 and K4me3 shows similar epigenomic patterns between genotypes within each state.

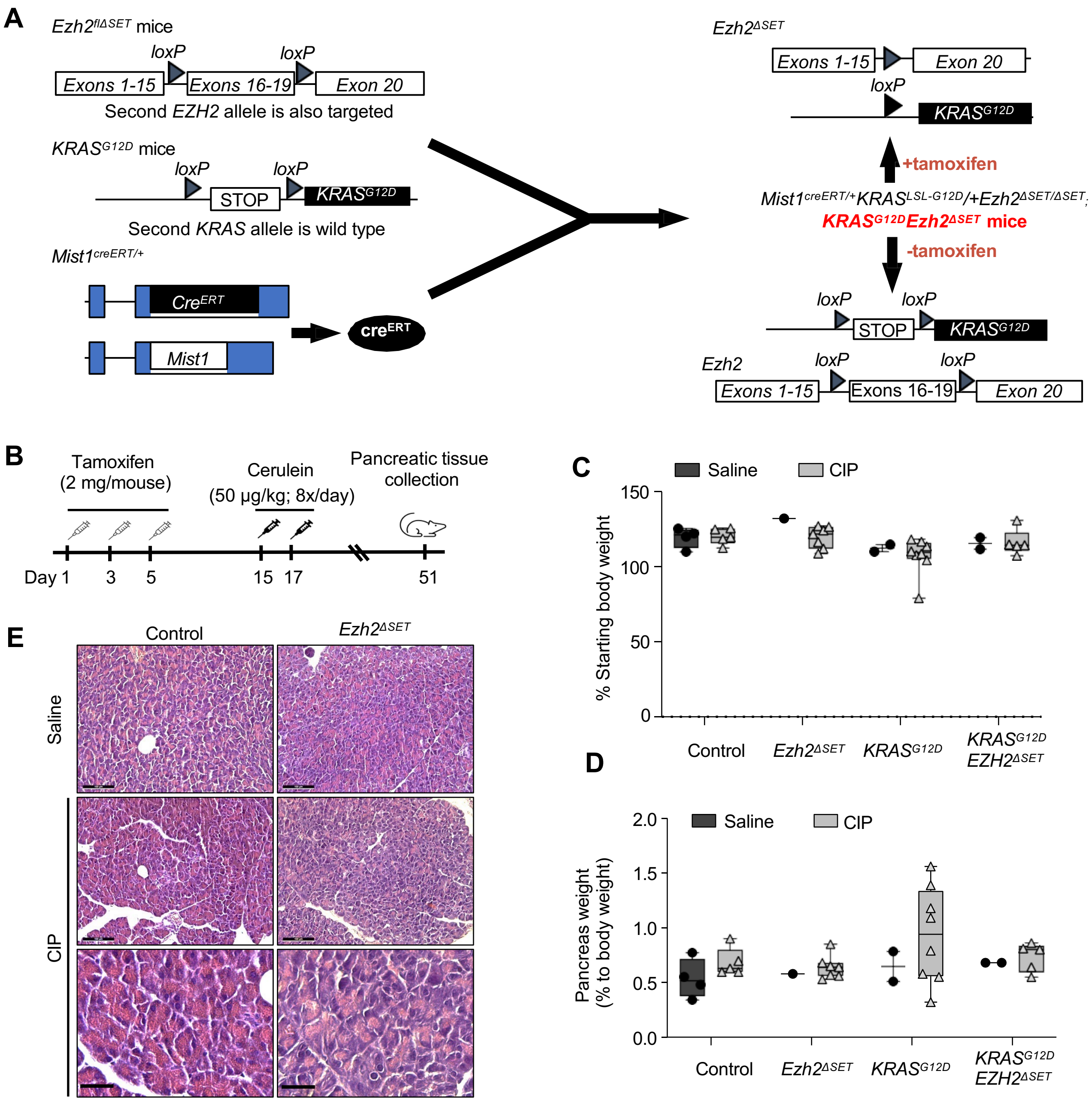

Figure S2. *EZH2*$^{\Delta SET}$ deletion does not prevent acinar cell regeneration during acute pancreatic injury. (A) Schematic representation of *KRAS*^{G12D} induction and *Ezh2*$^{\Delta SET}$ deletion (*KRAS*^{G12D}*Ezh2*$^{\Delta SET}$) by tamoxifen (TX) gavage specifically in acinar cells of mice driven by the *Mist1*$^{creERT/+}$ promoter. **(B)** Experimental timeline of TX gavage and acute cerulein-induced pancreatitis (CIP). Box plots show **(C)** changes in body weight between TX gavage and 51 days later, or **(D)** pancreatic weights at time of dissection in vehicle or cerulein-treated control, *Ezh2*$^{\Delta SET}$, *KRAS*^{G12D} and *KRAS*^{G12D}*Ezh2*$^{\Delta SET}$ mice. Data reflects the mean difference in weight ± min to max (n=1 mouse for *Ezh2*$^{\Delta SET}$ saline, n=2 mice for *KRAS*^{G12D} and *KRAS*^{G12D}*Ezh2*$^{\Delta SET}$ saline, n=4 mice for saline control, n=5 mice for control and *KRAS*^{G12D}*Ezh2*$^{\Delta SET}$ cerulein, n=7 mice for *Ezh2*$^{\Delta SET}$ cerulein and n=8 mice for *KRAS*^{G12D} cerulein). Significance was determined between conditions using a two-way ANOVA followed by a Tukey's post-hoc test. **(E)** Representative images of H&E-stained pancreatic sections from control and *Ezh2*$^{\Delta SET}$ mice 51 days after TX gavage and 25 days after initial cerulein treatment. Scale bar = 100 µm (top four images) or 50 µm (bottom two images).

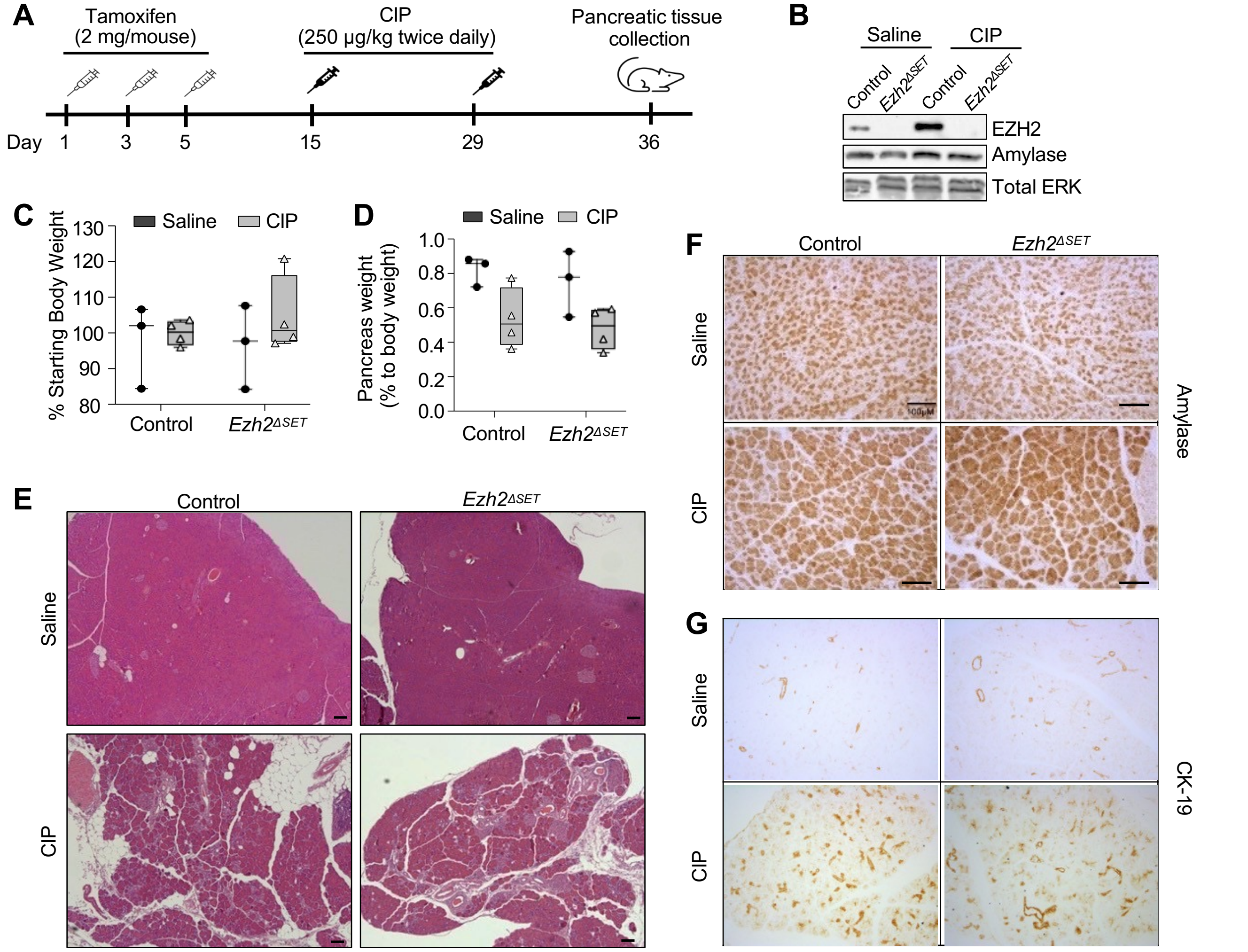

Figure S3. *EZH2$^{\Delta SET}$* deletion does not alter regeneration in a chronic model of pancreatic injury. (A) Experimental timeline for repetitive cerulein-induced pancreatic (CIP) injury in control and *Ezh2$^{\Delta SET}$* mice. 15 days after initial tamoxifen (TX) gavage, mice are injected twice daily for 14 days with 250 µg. Pancreatic tissue was collected 7 days after recurrent CIP treatment. **(B)** Representative western blot analysis for EZH2 or amylase in pancreatic tissue from control and *Ezh2$^{\Delta SET}$* mice treated with saline or CIP. Western blots for total ERK were used as a loading control. Box plots comparing **(C)** changes in body weight or **(D)** pancreatic weight at 36 days (experimental end point) in saline or CIP-treated control and *Ezh2$^{\Delta SET}$* mice. Data are shown as mean ± min to max (n=3 mice for control and *Ezh2$^{\Delta SET}$* saline and n=4 mice for control and *Ezh2$^{\Delta SET}$* CIP). Significance between conditions is measured using a two-way ANOVA followed by a Tukey's post-hoc test. Representative images of **(E)** H&E-staining or IHC for **(F)** amylase or **(G)** CK19 on pancreas sections from control or *Ezh2$^{\Delta SET}$* mice 36 days after TX gavage and 7 days after recurrent CIP treatment. Scale bar = 100 µm.

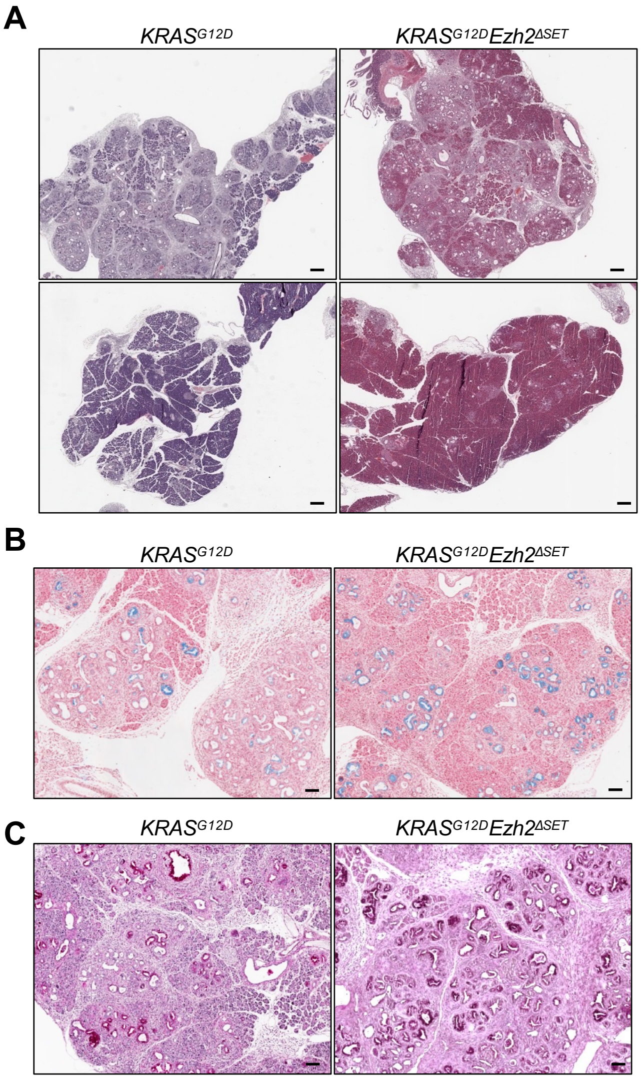

Figure S4. Low magnification images comparing morphology of *KRAS*^{G12D} and *KRAS*^{G12D}*Ezh2*$^{\Delta SET}$ pancreatic tissue. Representative low magnification images of **(A)** H&E histology, **(B)** Alcian blue histology, and **(C)** PAS histology of *KRAS*^{G12D} and *KRAS*^{G12D}*Ezh2*$^{\Delta SET}$ pancreatic tissue 51 days after tamoxifen gavage and 35 days after treatment with saline or cerulein. Images in **(A)** highlight the focal nature of lesions appearing in the pancreas as some regions (lower panels) show no overt lesions. Scale bar = 500 µm in A; 100 µm in B and C.

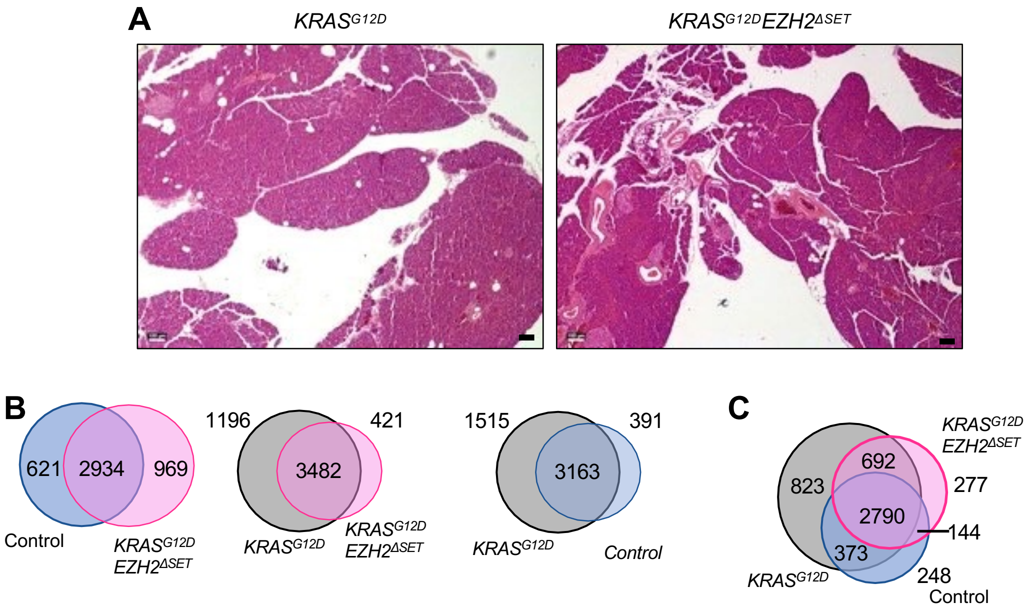

Figure S5. Effect of EZH2 methyltransferase deletion on mediated epigenetic reprogramming. (A) Representative images of H&E-stained pancreatic tissue from of *KRAS*^{G12D} and *KRAS*^{G12D}*Ezh2*$^{\Delta SET}$ mice 22 days after KRAS^{G12D} activation. Scale bar = 500 µm.

(B) Venn diagrams comparing the number of K27me3-enriched genes between control and *KRAS*^{G12D} or *KRAS*^{G12D}*Ezh2*$^{\Delta SET}$ tissue, or *KRAS*^{G12D} and *KRAS*^{G12D}*Ezh2*$^{\Delta SET}$ tissue. Genes were called when enrichment sites were within -100 kb to +3 kb of a gene transcriptional start site (TSS) based on ChIP-seq of pancreatic 22 days after tamoxifen gavage with no induction of injury. **(C)** Three-way comparison of K27me3-enriched genes showing the number of genes uniquely enriched for K27me3.

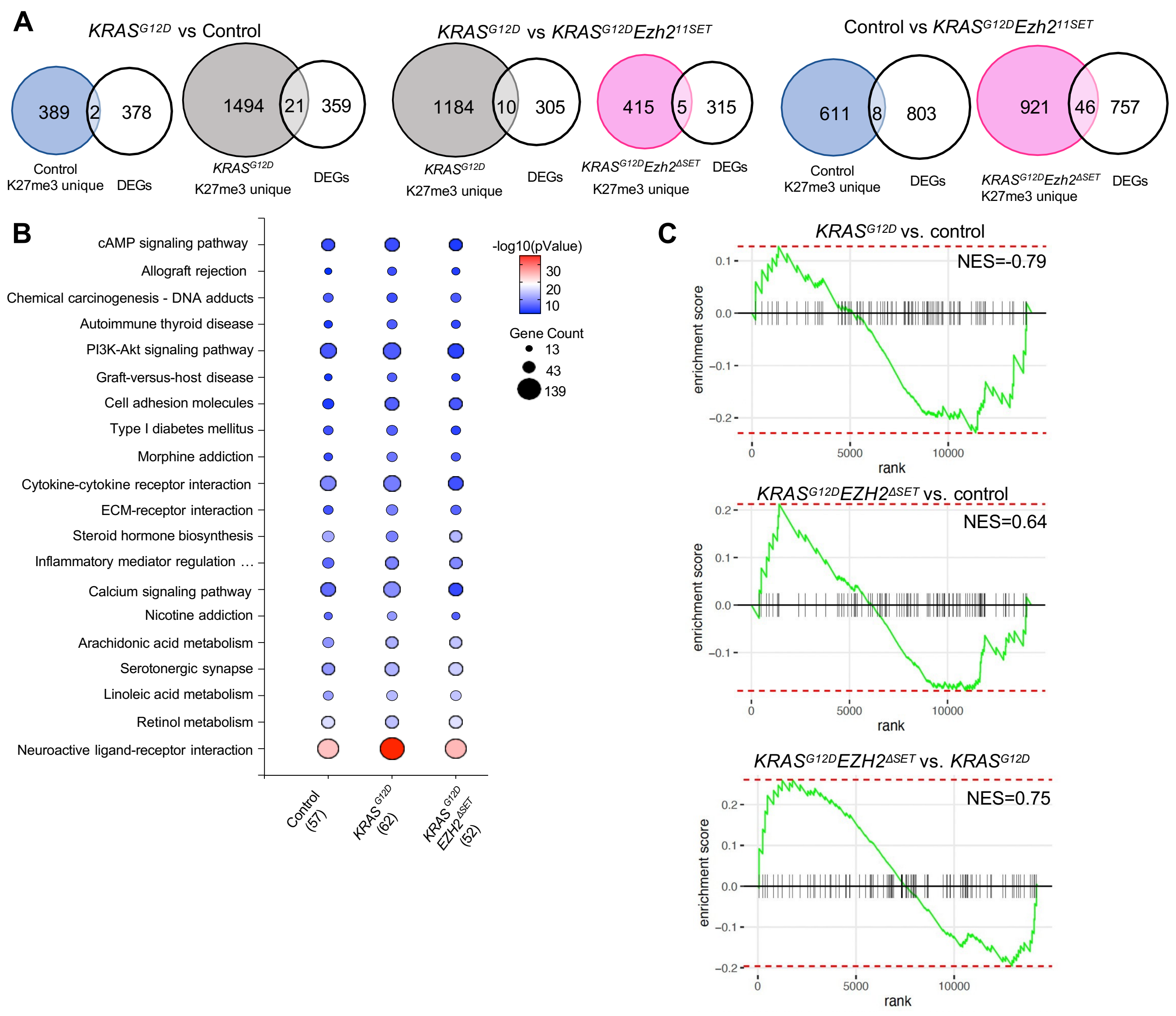

Figure S6. Loss of EZH2 alters *KRAS*^{G12D}-mediated epigenetic changes in the absence of transcriptional changes.
(A) Venn diagrams show integration of uniquely enriched genes for K27me3 based on ChIP-seq of pancreatic tissue vs. differentially expressed genes (DEGs) from control, *KRAS*^{G12D} and *KRAS*^{G12D}*Ezh2*$^{\Delta SET}$ mice 22 days after KRAS^{G12D} induction. **(B)** KEGG pathway analysis on state 2 genes, which are enriched for only K27me3. Number of pathways listed in brackets. Top 20 pathways are shown. **(C)** Gene Set Enrichment Analysis comparing enrichment of HALLMARK_PI3K-AKT-MTOR SIGNALING using DEGs between control, *KRAS*^{G12D} and *KRAS*^{G12D}*Ezh2*$^{\Delta SET}$ tissue 22 days following tamoxifen treatment. While no significance is identified between genotypes, normalized enrichment scores (NES) trend in different directions when control expression is compared to *KRAS*^{G12D}*Ezh2*$^{\Delta SET}$ or *KRAS*^{G12D} expression (n=3 mice).

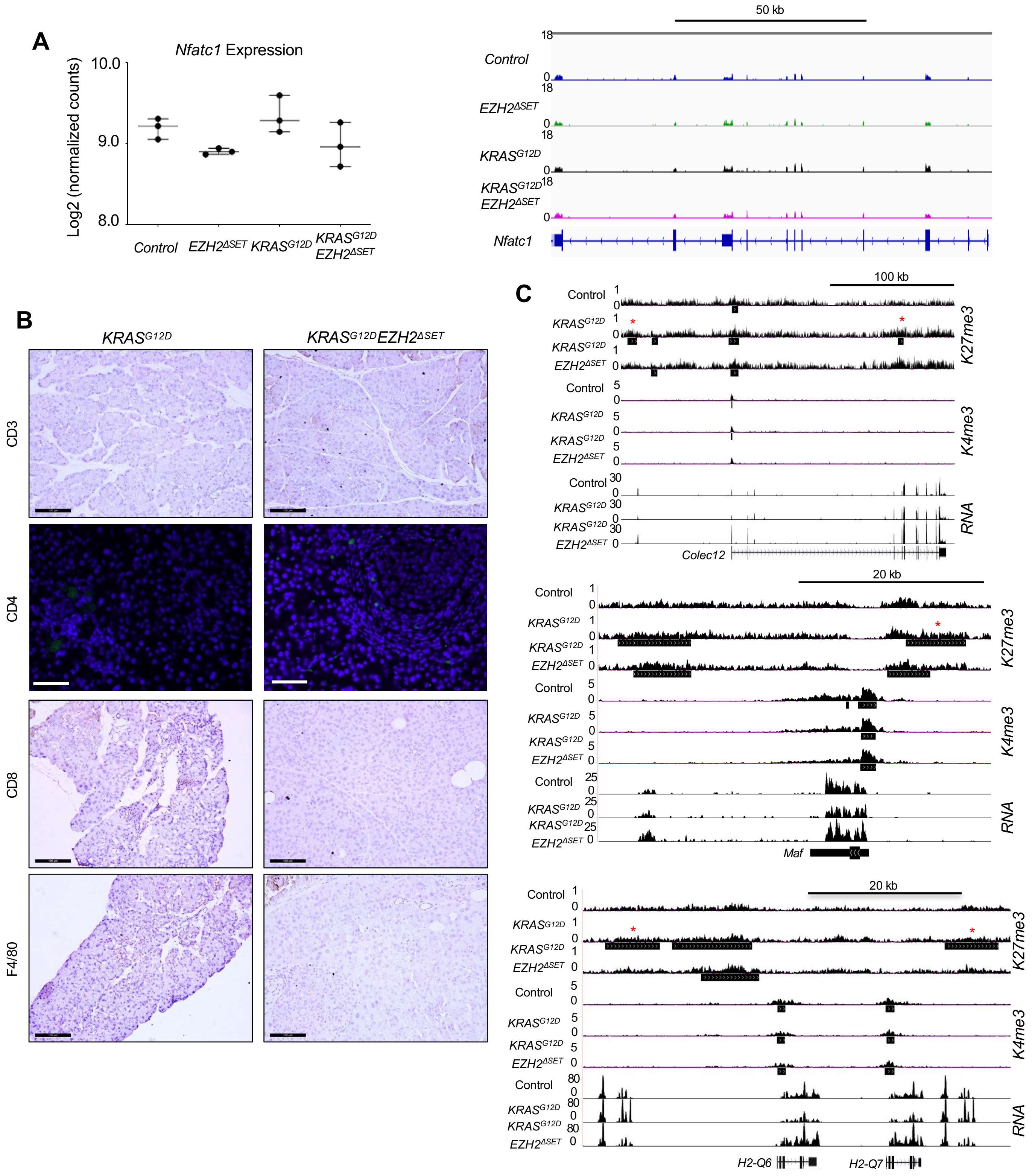

Figure S7. (A) *Nfatc1* gene expression and RNA-tracks based on RNA-Seq data. Significance measured by a one-way ANOVA followed by Tukey's post hoc test. Data are shown as mean ± min to max (n=3 mice/group). Tracks are an overlay of n=3 mice. **(B)** Representative images of IHC for CD3, CD8 and F4/80 or IF for CD4 on pancreatic section from $KRAS^{G12D}$ or $KRAS^{G12D}Ezh2^{\Delta SET}$ mice 22 days after tamoxifen. Sections are counterstained with hematoxylin or DAPI, respectively. Scale bar = 100 μm. **(C)** K27me3, K4me3 and RNA tracks for differentially expressed genes between $KRAS^{G12D}$ and $KRAS^{G12D}Ezh2^{\Delta SET}$ mice and linked to immune-related pathways including *Colec12*, *Maf*, *H2-Q6*, and *H2-Q7*. Red asterisks indicate K27me3 enrichment specific to $KRAS^{G12D}$ mice. Tracks are an overlay of n=3 mice.

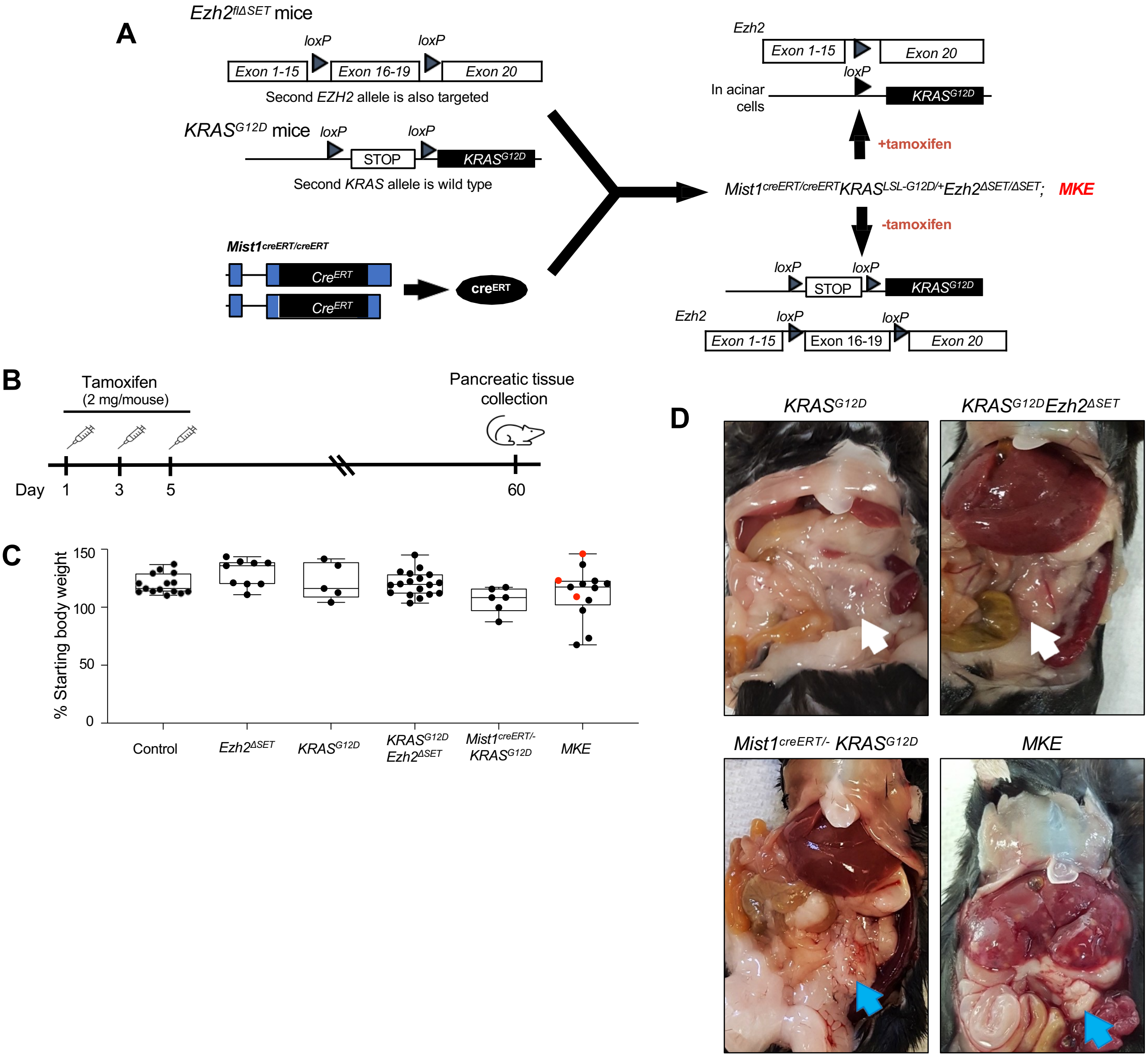

Figure S8. Combined absence of MIST1 and EZH2 methyltransferase activity increases *KRAS^{G12D}*-mediated PDAC progression. **(A)** Schematic diagram showing cre-mediated *KRAS^{G12D}* deletion of *EZH2$^{\Delta SET}$* induced by tamoxifen (TX) gavage specifically in acinar cells driven by *Mist1$^{creERT/-}$* promoter. **(B)** Timeline of experiment. Mice were treated with 2 mg of TX 3 times over five days and sacrificed 60 days later. **(C)** Final mouse body weight at 60 days relative to starting weights. Data shown as mean ± min to max (n=5 mice for *KRAS^{G12D}*, n=6 mice for *Mist1$^{creERT/-}$KRAS^{G12D}*, n=9 mice for *Ezh2$^{\Delta SET}$*, n=13 mice for *MKE*, n=15 mice for control and n=17 mice for *KRAS^{G12D}Ezh2$^{\Delta SET}$*). Red dots in *MKE* data represent mice sacrificed before the 60 day end point. Significance is measured compared to control mice by one-way ANOVA followed by Dunnett's correction. **(D)** Representative images of abdomen and pancreatic tissue in *KRAS^{G12D}*, *KRAS^{G12D}Ezh2$^{\Delta SET}$*, *Mist1$^{creERT/-}$KRAS^{G12D}* and *MKE* mice 60 days after TX gavage. Blue arrows indicate fibrotic nodules within the pancreas.

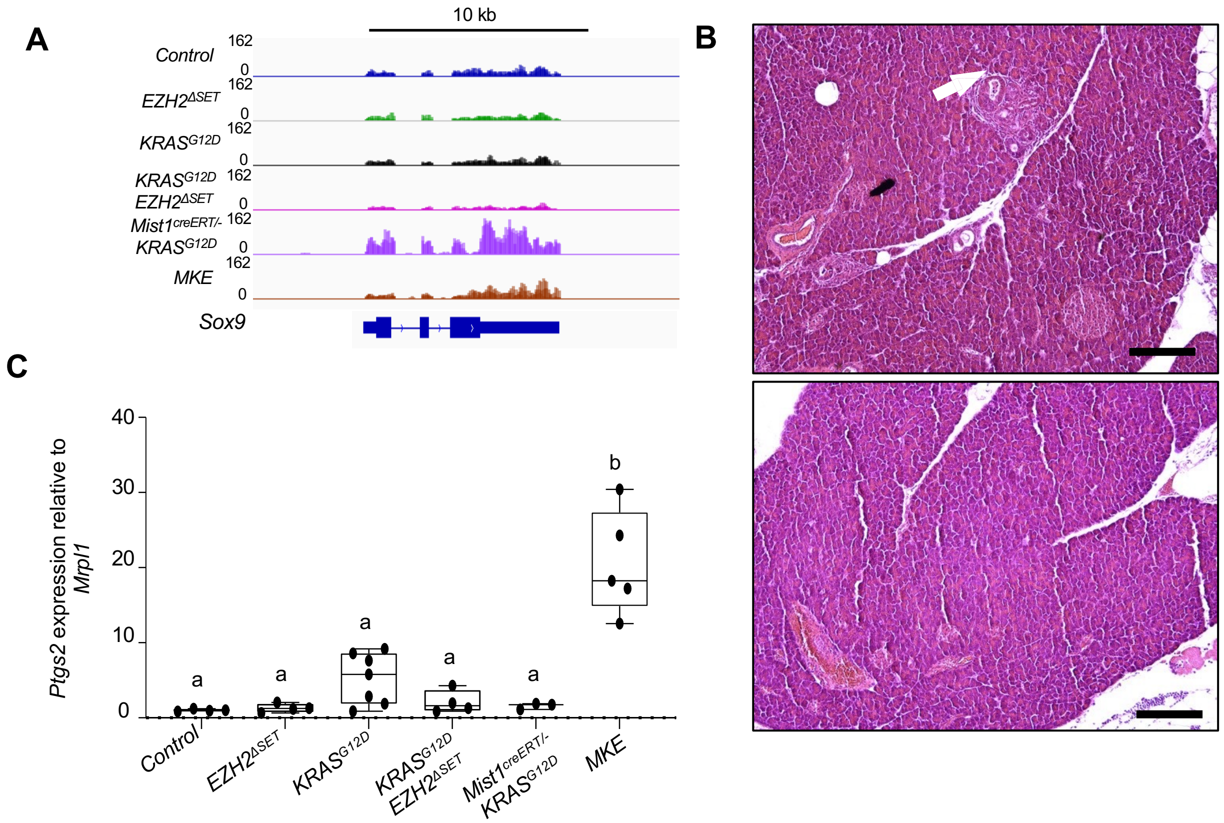

Supplemental Figure S9. (A) RNA tracks of *Sox9* for all genotypes. Tracks are the overlay of n=3 mice for control, *Ezh2*$^{\Delta SET}$, *KRAS*^{G12D}, *KRAS*^{G12D}*Ezh2*$^{\Delta SET}$ and *MKE* and n=2 mice for *Mist1*$^{creERT/-}$*KRAS*^{G12D}. **(B)** Two representative low magnification images of *MKE* pancreatic issue taken from two different mice 22 days after initial tamoxifen treatment. **(C)** qRT-PCR for *Ptgs2* expression in control, *Ezh2*$^{\Delta SET}$, *KRAS*^{G12D}, *KRAS*^{G12D}*Ezh2*$^{\Delta SET}$, *Mist1*$^{creERT/-}$*KRAS*^{G12D}, and *MKE* mice pancreatic tissue 22 days after KRAS^{G12D} induction. Data are shown as mean ± min to max (n=3 mice for *Mist1*$^{creERT/-}$*KRAS*^{G12D}, n=4 mice for control, *Ezh2*$^{\Delta SET}$ and *KRAS*^{G12D}*Ezh2*$^{\Delta SET}$, n=5 mice for *MKE* and n=7 mice for *KRAS*^{G12D}). Significance was determined with a one-way ANOVA followed by Tukey's correction post hoc analysis. Letters indicate statistically similar groups $p^{b} \leq 0.0001$.

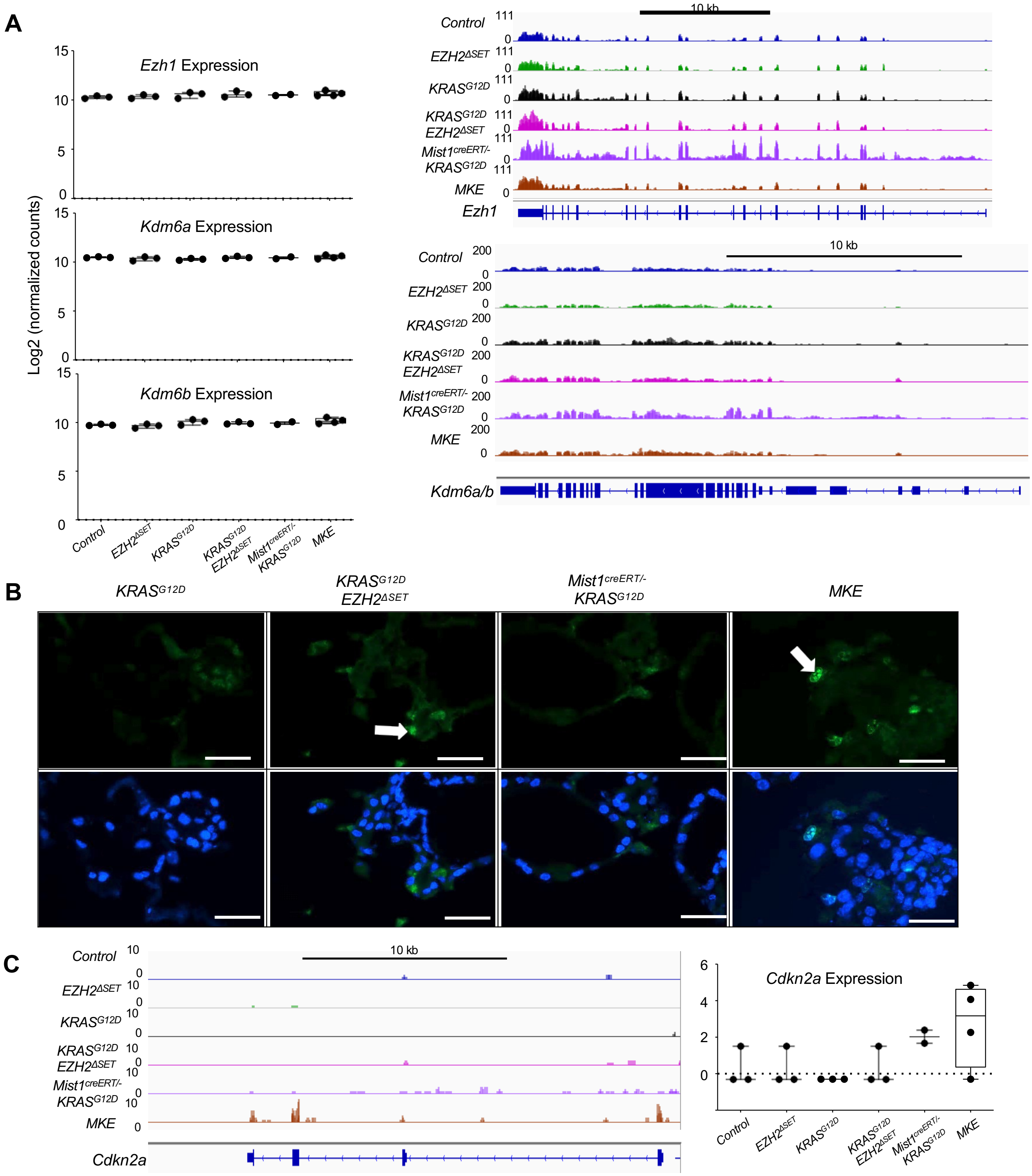

Figure S10. $EZH2^{\Delta SET}$ deletion increases proliferation in $Mist1^{creERT/-}$-$KRAS^{G12D}$- expressing cells.
(A) Genes encoding mediators of K27me3 show no difference in expression based on RNA-seq. RNA-tracks and quantification of *Ezh1* and *Kdm6a/b,* 22 days after initial tamoxifen treatment. **(B)** Representative immunofluorescence for Ki67 (green) in acinar cells seven days after isolation. Nuclei counterstained with DAPI (blue). White arrow represents KI67 positive staining. Scale bar = 50 µm. **(C)** RNA-tracks and quantification for *Cdnk2a* pancreatic tissue 22 days after initial tamoxifen treatment. For all graphs, data is shown as mean ± min to max (n=3 mice/group), significance measured using a one-way ANOVA followed by Tukey's post hoc test, and tracks are the overlay of n=3 mice.

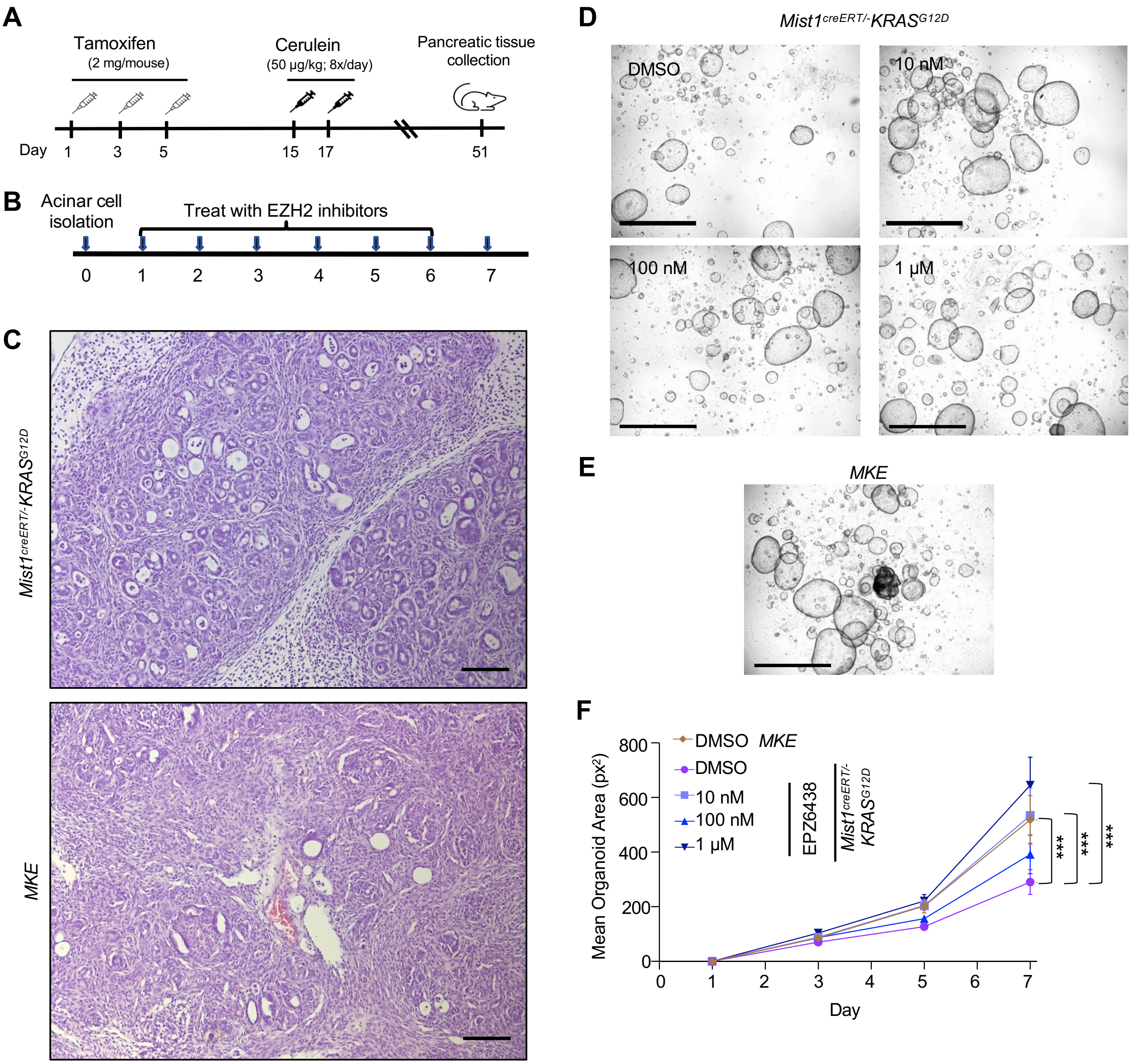

Figure S11. *EZH2$^{\Delta SET}$* deletion reduces organoid formation in *KRAS^{G12D}*-expressing cells.
(A) Experimental timeline of TX gavage and acute cerulein-induced pancreatitis. **(B)** Experimental design for *Mist1$^{creERT/-}$-KRAS^{G12D}* acinar cell isolation and treatment with the EZH2 inhibitor EPZ6438. **(C)** Representative images of *Mist1$^{creERT/-}$-KRAS^{G12D}* and *MKE* tissue at time of establishing organoid cultures. **(D, E)** Representative images of organoids from **(D)** *Mist1$^{creERT/-}$-KRAS^{G12D}* and **(E)** *MKE* mice 7 days with increasing concentrations of EPZ6438. Scale bar = 1.7 mm. **(F)** Quantification of the mean area of organoids one to seven days after treatment. Data are shown as mean ± SEM (n>400 organoids). Significance is measured at day 7 by two-way ANOVA followed by Tukey's correction. ***$p \leq 0.001$.

Supplementary Table 1. State 4 Top 20 KEGG pathways

State 4	Control			$KRAS^{G12D}$			$KRAS^{G12D}$ $EZH2^{\Delta SET}$		
KEGG Pathways	Count	pValue	Genes	Count	pValue	Genes	Count	pValue	Genes
mmu05168:Herpes simplex virus 1 infection	41	1.12E-06	ZFP964, ZFP788, ZFP128, GM14322, AU041133, ZFP268, GM14326, ZFP869, ZFP868, AW146154, H2-Q1, ZFP709, ZFP11, AKT3, ZFP560, ZFP120, ZFP141, ZFP641, MAP3K7, GM3055, ZFP386, ZFP661, ZFP975, ZFP951, ZFP114, ZFP433, GM10033, ZFP873, ZFP317, GM14391, ZFP938, ZFP958, POU2F2, AI987944, TRAF6, GM10778, ZFP157, SRSF6, ZFP26, ZFP551, H2-Q10	50	1.07E-05	ZFP964, ZFP128, GM14322, GM14326, AW146154, ZFP808, IKBKB, AKT3, ZFP120, ZFP560, MAP3K7, ZFP975, ZFP974, ZFP457, ZFP418, ZFP939, ZFP938, ZFP94, ZFP93, TRAF6, SRSF6, ZFP773, ZFP788, RSL1, AU041133, ZFP268, ZFP869, ZFP825, ZFP748, ZFP109, ZFP868, ZFP626, ZFP709, ZFP141, ZFP267, ZFP266, ZFP386, ZFP661, ZFP951, ZFP114, ZFP433, POU2F1, GM10033, ZFP873, ZFP715, GM14391, ZFP958, AI987944, ZFP157, ZFP551	46	5.93E-07	ZFP964, ZFP788, RSL1, ZFP128, GM14322, AU041133, GM14326, ZFP869, ZFP825, ZFP109, ZFP868, AW146154, ZFP729B, ZFP709, ZFP808, AKT3, ZFP560, ZFP120, ZFP141, MAP3K7, ZFP442, GM3055, ZFP386, ZFP661, ZFP975, ZFP457, ZFP951, ZFP114, ZFP433, GM10033, ZFP873, ZFP715, ZFP317, GM14391, ZFP938, ZFP94, ZFP958, ZFP93, POU2F2, AI987944, TRAF6, GM10778, ZFP157, SRSF6, ZFP551, NECTIN1
mmu04010:MAPK signaling pathway	20	0.021939113	PDGFRA, PTPRR, IL1R1, CACNA2D1, FLT4, INSR, VEGFC, CACNA1E, FGF2, AREG, FGF5, CACNG7, PPP3R1, CACNB4, DUSP10, ERBB4, TRAF6, AKT3, MAP3K7, PRKACB	34	1.42E-04	RET, PTPRR, FLT4, RASGRF1, CACNA1C, CACNA1E, AREG, IGF1R, IKBKB, FGF5, CACNG7, PPP3R1, DUSP10, ERBB4, PDGFC, AKT3, KDR, MAP3K7, PRKACB, PRKCG, PDGFRA, IL1R1, CACNA2D1, INSR, VEGFC, CACNA2D2, IGF1, MAPK10, PPM1A, FGF15, CACNB4, TRAF6, RASA1, MAPT	25	0.00397035	PTPRR, FLT4, RASGRF1, CACNA1C, RASGRP1, AREG, FGF5, CACNG7, PPP3R1, DUSP10, ERBB4, PDGFC, AKT3, KDR, MAP3K7, PRKACB, IL1R1, CACNA2D1, VEGFC, CACNA2D2, MAPK10, PPM1A, CACNB4, TRAF6, RASA1
mmu04310:Wnt signaling pathway	13	0.037658408	CAMK2D, LEF1, PPP3R1, SFRP1, SFRP2, DAAM1, PEG12, SOX17, WIF1, RSPO3, MAP3K7, LGR5, PRKACB	22	7.75E-04	PRKCG, FZD1, TLE3, CAMK2D, LEF1, WNT9B, NKD1, MAPK10, PPP3R1, SFRP1, VANGL2, SFRP2, DAAM1, PEG12, SOX17, WIF1, DAAM2, RSPO3, PLCB1, MAP3K7, LGR5, PRKACB			
mmu05412:Arrhythmogenic right ventricular cardiomyopathy	10	0.005659987	SLC8A3, CACNG7, GJA1, DTNA, CACNB4, SGCB, CACNA2D1, LEF1, ITGA8, ITGB8	14	9.40E-04	CACNA2D1, LEF1, CACNA2D2, CACNA1C, SLC8A3, CACNG7, GJA1, CACNB4, SGCD, CDH2, ITGA8, SSPN, ITGB8, CTNNA3	12	0.00150296	SLC8A3, CACNG7, GJA1, CACNB4, LAMA1, CACNA2D1, LEF1, ITGA8, CACNA2D2, ITGB8, CTNNA3, CACNA1C
mmu04360:Axon guidance	13	0.04672465	EPHA5, NTNG1, NTNG2, ROBO3, CAMK2D, SEMA3D, SEMA6D, UNC5C, UNC5D, PPP3R1, SLIT2, EPHB1, PLXNA4	22	0.00120061	ROBO2, EPHA5, NTNG1, EPHB6, NTNG2, ROBO3, EPHA4, CAMK2D, UNC5A, SEMA3D, UNC5B, SEMA3A, SEMA6D, UNC5C, UNC5D, PPP3R1, CXCL12, RASA1, SLIT2, PLXNC1, EPHB1, PLXNA4	19	0.00123188	ROBO2, EPHA5, NTNG1, ROBO3, EPHA4, CAMK2D, TRPC3, UNC5A, SEMA3D, UNC5B, SEMA6D, UNC5C, UNC5D, PPP3R1, CXCL12, RASA1, SLIT2, EPHB1, PLXNA4
mmu05414:Dilated cardiomyopathy	10	0.017523784	SLC8A3, CACNG7, DTNA, CACNB4, SGCB, CACNA2D1, ITGA8, ITGB8, ADCY8, PRKACB	14	0.00485095	CACNA2D1, CACNA2D2, ADCY2, CACNA1C, IGF1, TTN, SLC8A3, CACNG7, CACNB4, SGCD, ITGA8, SSPN, ITGB8, PRKACB	13	0.00210966	LAMA1, CACNA2D1, CACNA2D2, ADCY2, CACNA1C, ADCY8, TTN, SLC8A3, CACNG7, CACNB4, ITGA8, ITGB8, PRKACB
mmu04213:Longevity regulating pathway - multiple species				10	0.00654428	HDAC2, CLPB, INSR, AKT3, ADCY2, IGF1, PRKACB, FOXO1, FOXA2, IGF1R			
mmu00062:Fatty acid elongation				6	0.02113267	PRKCG, CHRM2, CAMK2D, CHRNA7, ADCY2, CACNA1C, GNG11, GNAO1, GNG2, CREB1, AKT3, PLCB1, PRKACB, CREB5			
mmu00230:Purine metabolism				15	0.01790623	PRKCG, SLC1A2, ADCY2, CACNA1C, SLC1A6, GNG11, GRM3, GNAO1, PPP3R1, GNG2, GRM6, GRM8, PLCB1, PRKACB	13	0.01684481	GUCY1A2, GUCY1A1, PDE1C, NPR2, ADCY2, AK5, ADCY8, PDE10A, ATIC, PDE4B, PDE7B, NUDT16, PDE7A
mmu04014:Ras signaling pathway				23	0.01311339	PRKCG, PDGFRA, SHC3, FLT4, INSR, RASGRF1, VEGFC, IGF1, GNG11, ETS1, IGF1R, FGF5, MAPK10, IKBKB, FGF15, GNG2, RASA1, PDGFC, AKT3, KDR, RGL1, PRKACB, EXOC2			
mmu04151:PI3K-Akt signaling pathway				31	0.02394061	NEUROD1, HHEX, RFX6, HNF4G, NKX6-1, FOXA2			
mmu04510:Focal adhesion				20	0.01857578	GUCY1A2, GUCY1A1, PDE1C, NPR2, PDE4D, ADCY2, AK5, FHIT, DCK, ATIC, PDE4B, NUDT16, PDE7A, XDH, ADA			
mmu04540:Gap junction				11	0.02166948	PRKCG, GNAO1, GUCY1A2, GNAZ, GRID2, GUCY1A1, IGF1, PLCB1, IGF1R			
mmu04713:Circadian entrainment	9	0.036645411	GNAO1, GUCY1A2, GUCY1A1, CAMK2D, GNB4, ADCY8, GNG11, PRKACB, PRKG1	12	0.02244912	PRKCG, PDGFRA, SHC3, LAMA4, FLT4, RASGRF1, VEGFC, LAMC2, IGF1, IGF1R, COL1A1, MAPK10, PDGFC, AKT3, RAPGEF1, ITGA8, KDR, ITGB8, FLNB, PIP5K1B			
mmu04724:Glutamatergic synapse	10	0.033009801	GNAO1, PPP3R1, GRIN3A, GRM8, GNB4, GRIK4, ADCY8, GNG11, SHANK3, PRKACB	14	0.01204396	ELOVL4, PPT1, THEM4, HSD17B12, ACOT3, HACD4			
mmu04725:Cholinergic synapse	11	0.011614849	CHRM2, GNAO1, CAMK2D, CHRNA7, AKT3, GNB4, ADCY8, GNG11, PRKACB, KCNJ2, CREB5	14	0.01047269	PRKCG, TJP1, PDGFRA, GUCY1A2, GJA1, GUCY1A1, TUBB2B, PDGFC, ADCY2, PLCB1, PRKACB	12	0.01219492	CHRM2, GNAO1, CAMK2D, GNG2, CHRNA7, AKT3, ADCY2, CACNA1C, ADCY8, PRKACB, KCNJ2, CREB5
mmu04730:Long-term depression				9	0.01813905	PRKCG, GNAO1, GUCY1A2, GUCY1A1, CAMK2D, CREB1, GNG2, ADCY2, CACNA1C, PLCB1, GNG11, PRKACB			
mmu04950:Maturity onset diabetes of the young				6	0.01574552	RET, HDAC2, CAMK2D, LAMA4, MGST3, FLT4, LEF1, IL4RA, LAMC2, LPAR3, ADCY2, ETS1, FOXO1, IGF1R, DLL3, GLI2, IKBKB, FGF5, GNG2, PEG12, HEY2, AKT3, CTNNA3, RXRG, PRKACB, PRKCG, FZD1, PDGFRA, CDKN2A, IL15, F2R, WNT9B, VEGFC, MITF, IGF1, GNG11, ESR1, MMP9, MAPK10, FGF15, CXCL12, TRAF6, PLCB1	5	0.0307191	NEUROD1, RFX6, HNF4G, NKX6-1, FOXA2
mmu05200:Pathways in cancer				43	0.02337555	RET, CHRM2, LAMA4, FLT4, IL4RA, LAMC2, LPAR3, AREG, IGF1R, IKBKB, FGF5, GNG2, ERBB4, PDGFC, AKT3, KDR, THEM4, ITGB8, PDGFRA, INSR, MAGI2, F2R, VEGFC, IGF1, GNG11, COL1A1, FGF15, CREB1, ITGA8, SGK1, CREB5			
mmu05410:Hypertrophic cardiomyopathy				12	0.02400005	SLC8A3, CACNG7, SGCD, CACNB4, CACNA2D1, ITGA8, SSPN, CACNA2D2, ITGB8, CACNA1C, IGF1, TTN	10	0.03476237	SLC8A3, CACNG7, CACNB4, LAMA1, CACNA2D1, ITGA8, CACNA2D2, ITGB8, CACNA1C, TTN

Supplementary Table 2. State 2 Top 20 KEGG pathways

State 2	Control			$KRAS^{G12D}$			$KRAS^{G12D}$ $EZH2^{\Delta SET}$		
KEGG Pathways	Count	pValue	Genes	Count	pValue	Genes	Count	pValue	Genes
mmu04080:Neuroactive ligand-receptor interaction	104	3.43E-24	CHRM3, NPY2R, HTR4, RXFP1, RXFP3, EDNRA, HTR7, PTGDR, PAQR9, GHSR, LHCGR, PDYN, GABRG3, PRLR, GABRG2, TRHR, GABRG1, ADCYAP1, MTNR1B, IAPP, GRIA1, PTGFR, GRIA2, CHRNA3, CALCB, FPR3, FPR2, NTS, GRIN2A, CALCR, NPBWR1, HRH4, DRD1, TAC1, DRD2, DRD3, DRD5, GRIA4, HTR1F, HTR1A, GRIN2B, NMS, NMUR2, GABRB3, GABRB2, NPY6R, GRIK3, GRIK1, PRL, HTR2A, GRIK2, ADRA1B, ADRA1A, GRM1, GRM3, GABRR3, GRM5, GRM7, UCN3, QRFPR, GRID2, EDN3, NPY5R, AVPR1B, GRID1, FSHB, GLP2R, TACR3, PTH2R, TACR1, AVPR1A, OPRM1, ADRA2A, SSTR4, MC3R, VGF, FSHR, NPSR1, GRP, PATE1, GLRA1, PATE6, PATE7, GLRA3, PATE4, PATE5, NPVF, PATE2, PATE3, GABRA2, GABRA6, GABRA5, GZMA, OPRK1, HTR5A, TSHR, HCRTR2, GRIN1, GLRB, SST, LEP, QRFPRL, VIP, PATE10	139	9.11E-36	CHRM3, OXTR, NPY2R, HTR4, RXFP1, RXFP2, RXFP3, EDNRA, EDNRB, CYSLTR2, HTR7, LEPR, PTGDR, PAQR9, GHSR, LHCGR, PDYN, GABRG3, PRLR, GABRG2, TRHR, GABRG1, UTS2B, ADCYAP1, MTNR1B, IAPP, GRIA1, PTGFR, GRIA2, CHRNA3, ADCYAP1R1, CALCRL, CALCB, CHRNA4, CHRNA6, FPR1, GPR83, FPR3, FPR2, NTS, CRHR1, MC2R, HRH1, APELA, GRIN2A, CALCR, HRH2, NPBWR1, GALR1, HRH4, DRD1, TAC1, DRD2, DRD3, NTSR1, DRD4, DRD5, GRIA4, HTR1F, HTR1A, TRPV1, GRIN2B, GRIN3A, NMS, NMUR2, GABRB3, GABRB2, UCN, THRB, NPY6R, GRIK3, ADRA1D, GRIK1, HTR2A, GRIK2, ADRA1B, ADRA1A, GRM1, MC4R, GABRR3, GRM5, GRM7, UCN3, GNRHR, PRLHR, CTSG, QRFPR, TSHB, EDN3, NPY5R, GRID1, FSHB, GLP2R, PTH2R, TACR1, AVPR1A, OPRM1, ADRA2C, ADRA2A, AGTR1A, SSTR4, MC3R, ADRB3, VGF, FSHR, NPSR1, GRP, PATE1, NPS, GLRA1, PATE7, GLRA3, PATE4, NPVF, P2RY1, PATE3, GABRD, GABRA2, GABRA1, GABRP, GABBR2, GABRA6, GABRA5, GZMA, OPRK1, CCK, GCG, HTR5A, HTR5B, HCRTR2, GRIN1, GLRB, SST, LEP, QRFPRL, HC, VIP, PATE10, F2RL3	110	5.84E-25	CHRM3, NPY2R, HTR4, RXFP1, RXFP2, RXFP3, EDNRA, EDNRB, CYSLTR2, HTR7, PTGDR, PAQR9, GHSR, LHCGR, CHRNB3, PDYN, GABRG3, PRLR, GABRG2, TRHR, ADCYAP1, MTNR1B, IAPP, GRIA1, PTGFR, GRIA2, ADCYAP1R1, CALCB, CHRNA6, GPR83, FPR2, NTS, CRHR1, GRIN2A, CALCR, HRH2, NPBWR1, GALR1, HRH4, DRD1, TAC1, DRD2, DRD3, DRD5, HTR1F, HTR1A, TRPV1, GRIN2B, NMS, NMUR2, GABRB3, GABRB2, UCN, NPY6R, GRIK3, GRIK1, TRH, HTR2A, GRIK2, ADRA1B, GRM1, GRM3, MC4R, GABRR3, GRM5, GRM7, UCN3, GNRHR, QRFPR, TSHB, EDN3, NPY5R, GRID1, FSHB, GLP2R, PTH2R, TACR1, AVPR1A, OPRM1, ADRA2A, AGTR1A, SSTR4, MC3R, VGF, NPSR1, GRP, PATE1, NPS, GLRA1, PATE6, PATE7, NPVF, P2RY1, GABRA1, GABRP, GABBR2, GABRA6, GABRA5, GZMA, OPRK1, GCG, HTR5A, HCRTR2, GRIN1, GLRB, SST, QRFPRL, HC, VIP, PATE10
mmu00830:Retinol metabolism	41	2.13E-16	CYP4A12A, CYP4A12B, CYP4A30B, CYP3A11, CYP3A57, CYP3A59, CYP2B19, CYP3A16, CYP2C39, CYP4A29, CYP2C38, CYP2B9, CYP2C37, CYP2B13, AOX4, CYP2A12, CYP2C54, RDH10, CYP2B10, AOX2, AOX3, CYP2C50, CYP4A31, CYP3A44, CYP4A14, CYP3A25, CYP2C29, RPE65, DHRS4, CYP2C69, CYP26A1, CYP2C68, CYP2C67, CYP2A5, CYP2C66, CYP2B23, CYP2C65, CYP3A41A, CYP2A22, CYP3A41B, CYP2C40	43	3.65E-14	CYP4A12A, CYP4A12B, CYP4A30B, CYP3A11, CYP3A57, CYP3A59, CYP2B19, CYP3A16, CYP2C39, CYP4A29, CYP2C38, CYP2B9, CYP2C37, CYP26B1, AOX4, CYP2C55, CYP2A12, CYP2C54, RDH10, CYP2B10, AOX3, CYP2C50, CYP4A31, CYP4A32, CYP3A44, CYP4A14, CYP3A25, CYP2C29, RPE65, DHRS4, ALDH1A3, CYP2C69, CYP26A1, CYP2C68, CYP2C67, CYP2A5, CYP2C66, CYP2B23, CYP2C65, CYP3A41A, CYP2A22, CYP3A41B, CYP2C40	43	7.53E-17	CYP4A12A, CYP4A12B, CYP4A30B, CYP3A11, CYP3A57, CYP3A59, CYP2B19, CYP3A16, CYP2C39, CYP4A29, CYP2C38, CYP2B9, CYP2C37, CYP2B13, AOX4, CYP2C55, CYP2A12, CYP2C54, RDH10, CYP2B10, AOX2, AOX3, CYP2C50, AOX1, CYP4A31, CYP4A10, CYP4A32, CYP3A44, CYP4A14, CYP3A25, CYP2C29, RPE65, CYP2C69, CYP26A1, CYP2C68, CYP2C67, CYP2A5, CYP2C66, CYP2B23, CYP2C65, CYP3A41A, CYP3A41B, CYP2C40
mmu00591:Linoleic acid metabolism	25	1.67E-11	CYP3A11, CYP3A57, CYP3A59, CYP3A16, CYP2C39, CYP2C38, CYP2C37, CYP2C54, CYP2C50, CYP2J8, CYP3A44, PLA2G4A, CYP3A25, CYP2C29, CYP2C69, CYP2C68, CYP2J11, CYP2C67, CYP2J12, CYP2C66, CYP2C65, CYP3A41A, CYP3A41B, CYP2C40, CYP2E1	30	1.47E-13	ALOX15, CYP3A11, CYP3A57, CYP3A59, CYP3A16, CYP2C39, CYP2C38, CYP2C37, CYP2C55, CYP2C54, CYP2C50, CYP2J5, PLA2G2C, CYP2J8, CYP3A44, PLA2G4A, CYP3A25, CYP2C29, CYP2C69, CYP2C68, CYP2J11, CYP2C67, CYP2J12, CYP2C66, CYP2J13, CYP2C65, CYP3A41A, CYP3A41B, CYP2C40, CYP2E1	29	1.74E-14	ALOX15, CYP3A11, CYP3A57, CYP3A59, CYP3A16, CYP2C39, CYP2C38, CYP2C37, CYP2C55, CYP2C54, CYP2C50, CYP2J5, CYP2J8, CYP3A44, PLA2G4A, CYP3A25, CYP2C29, CYP2C69, CYP2C68, CYP2J11, CYP2C67, CYP2J12, CYP2C66, CYP2J13, CYP2C65, CYP3A41A, CYP3A41B, CYP2C40, CYP2E1
mmu04726:Serotonergic synapse	40	4.59E-11	GABRB3, GABRB2, TPH2, APP, CACNA1B, CACNA1C, HTR2A, HTR4, CYP2C39, CYP2C38, CYP2C37, CYP2D12, CYP2D34, CYP2D9, HTR7, GNG2, CYP4X1, CYP2C54, GNG4, CYP2C50, KCNN2, KCNJ3, KCNJ5, KCNJ6, HTR1F, CYP2J8, HTR1A, PLA2G4A, HTR3B, HTR5A, CYP2C29, CYP2C69, CYP2C68, CYP2J11, CYP2C67, CYP2J12, CYP2C66, CYP2C65, CYP2D40, CYP2C40	49	4.14E-13	GABRB3, APP, GABRB2, HTR2A, HTR4, CYP2C39, CYP2C38, CYP2C37, CYP2D9, CYP2D12, HTR7, KCND2, PLA2G4A, HTR3A, HTR3B, CYP2J11, CYP2J12, CYP2J13, CYP2C40, TPH2, ALOX15, CACNA1B, ITPR2, ADCY5, CYP2D34, CYP4X1, CYP2C55, CYP2C54, GNG4, ALOX12E, CYP2C50, KCNN2, KCNJ3, KCNJ5, KCNJ6, CYP2J5, HTR1F, CYP2J8, HTR1A, HTR5A, CYP2C29, HTR5B, CYP2C69, CYP2C68, CYP2C67, CYP2C66, CYP2C65, CYP2D40, GNB4	48	3.06E-15	GABRB3, GABRB2, TPH2, APP, ALOX15, CACNA1B, ITPR2, HTR2A, HTR4, CYP2C39, ADCY5, CYP2C38, CYP2C37, CYP2D12, CYP2D34, CYP2D9, HTR7, CYP4X1, CYP2C55, CYP2C54, GNG4, ALOX12E, CYP2C50, KCNN2, KCNJ3, KCNJ5, KCNJ6, CYP2J5, KCND2, HTR1F, CYP2J8, HTR1A, PLA2G4A, HTR3A, HTR3B, HTR5A, CYP2C29, CYP2C69, CYP2C68, CYP2J11, CYP2C67, CYP2J12, CYP2C66, CYP2J13, CYP2C65, CYP2D40, GNB4, CYP2C40
mmu00590:Arachidonic acid metabolism	31	9.02E-11	CYP4A12A, CYP4A12B, CYP4A30B, CYP2B19, CYP2C39, CYP4A29, CYP2C38, CYP2B9, CYP2C37, HPGDS, CYP2B13, CYP2C54, CYP2B10, CYP2C50, CBR3, PTGIS, CYP4A31, CYP2J8, PLA2G4A, CYP4A14, CYP2C29, CYP2C69, CYP2C68, CYP2J11, CYP2C67, CYP2J12, CYP2C66, CYP2B23, CYP2C65, CYP2C40, CYP2E1	38	5.69E-13	CYP4A12A, CYP4A12B, CYP4A30B, ALOX15, CYP2B19, CYP2C39, CYP4A29, CYP2C38, CYP2B9, CYP2C37, HPGDS, CYP2C55, CYP2C54, CYP2B10, ALOX12E, CYP2C50, CBR3, CBR1, PTGIS, CYP2J5, PLA2G2C, CYP4A31, CYP2J8, CYP4A32, PLA2G4A, CYP4A14, CYP2C29, CYP2C69, CYP2C68, CYP2J11, CYP2C67, CYP2J12, CYP2C66, CYP2B23, CYP2J13, CYP2C65, CYP2C40, CYP2E1	37	1.84E-14	CYP4A12A, CYP4A12B, CYP4A30B, ALOX15, CYP2B19, CYP2C39, CYP4A29, CYP2C38, CYP2B9, CYP2C37, HPGDS, CYP2B13, CYP2C55, CYP2C54, CYP2B10, ALOX12E, CYP2C50, CBR3, CYP2J5, CYP4A31, CYP2J8, CYP4A10, CYP4A32, PLA2G4A, CYP4A14, CYP2C29, CYP2C69, CYP2C68, CYP2J11, CYP2C67, CYP2J12, CYP2C66, CYP2B23, CYP2J13, CYP2C65, CYP2C40, CYP2E1
mmu05033:Nicotine addiction	17	1.82E-07	GABRB3, GRIA1, GABRA2, GABRB2, GRIA2, GABRA6, GABRA5, CACNA1B, GABRG3, GRIN2B, GABRG2, GABRG1, GRIN1, GABRR3, GRIN2A, SLC17A6, GRIA4	23	4.39E-11	GABRB3, GRIA1, GABRA2, GABRP, GABRB2, GRIA2, GABRA1, GABRA6, GABRA5, CHRNA4, CHRNA6, CACNA1B, GABRG3, GRIN2B, GABRG2, GABRG1, GRIN1, GABRR3, GRIN2A, GRIN3A, SLC17A6, GABRD, GRIA4	17	5.27E-07	GABRB3, GRIA1, GABRP, GABRB2, GRIA2, GABRA1, GABRA6, GABRA5, CHRNA6, CACNA1B, GABRG3, GRIN2B, GABRG2, GRIN1, GABRR3, GRIN2A, SLC17A6
mmu04020:Calcium signaling pathway	52	6.47E-08	RYR2, CHRM3, FLT1, HTR2A, ADRA1B, HTR4, ADRA1A, SLC8A1, GRM1, RYR3, SLC8A2, MYLK4, FGF6, PPP3CA, GRM5, EDNRA, HTR7, SLN, NOS1, LHCGR, AVPR1B, TACR3, TACR1, AVPR1A, TRHR, TRDN, PTGFR, PDE1A, CACNA1B, CXCR4, ADCY2, CACNA1C, ADCY1, GRIN2A, FGF20, DRD1, FGF23, DRD5, NTRK1, NTRK2, MCOLN3, NTRK3, PHKB, ATP2B1, HTR5A, GRIN2B, GRIN1, GNAL, GDNF, FGF18, FGFR2, FGF10	67	2.31E-10	RYR1, RYR2, CHRM3, OXTR, FLT1, ADRA1D, HTR2A, ADRA1B, HTR4, ADRA1A, SLC8A1, FGF3, RYR3, GRM1, MYLK, FGF4, MYLK4, FGF6, PPP3CA, GRM5, EDNRA, EDNRB, CYSLTR2, HTR7, FGF8, SLN, FGF9, NOS1, LHCGR, HGF, TACR1, AVPR1A, AGTR1A, TRHR, ADRB3, PTGFR, PDE1B, PDE1A, CACNA1B, ITPR2, CXCR4, ADCY1, ADCY8, GRIN2A, HRH1, HRH2, PDGFD, FGF20, DRD1, FGF23, NTSR1, DRD5, NTRK2, MCOLN3, NTRK3, PHKB, HTR5A, HTR5B, GRIN2B, GRIN1, GRIN3A, GNAL, GDNF, CAMK4, FGF18, FGFR2, FGF10	48	1.97E-05	PTGFR, RYR2, CHRM3, FLT1, PDE1A, CACNA1B, CXCR4, ITPR2, HTR2A, HTR4, ADRA1B, CACNA1E, GRM1, RYR3, FGF4, MYLK4, FGF6, GRIN2A, GRM5, EDNRA, CYSLTR2, HTR7, EDNRB, SLN, FGF9, HRH2, FGF20, NOS1, DRD1, FGF23, DRD5, NTRK2, LHCGR, NTRK3, PHKB, ATP2B2, TACR1, AVPR1A, HTR5A, GRIN2B, AGTR1A, GRIN1, TRHR, GNAL, GDNF, FGF18, FGFR2, FGF10
mmu04750:Inflammatory mediator regulation of TRP channels	33	1.69E-07	CYP4A12A, CYP4A12B, CYP4A30B, ADCY2, HTR2A, ADCY1, CYP2C39, CYP4A29, CYP2C38, CYP2C37, ASIC5, CYP2C54, CYP2C50, ASIC2, MAP2K6, NTRK1, TRPA1, CYP4A31, CYP2J8, PLA2G4A, CYP4A14, CYP2C29, MAPK10, CYP2C69, CYP2C68, CYP2J11, CYP2C67, CYP2J12, CYP2C66, CYP2C65, IL1B, CYP2C40, PRKCQ	42	1.47E-09	CYP4A12A, CYP4A12B, CYP4A30B, ITPR2, HTR2A, ADCY1, ADCY8, CYP2C39, CYP4A29, ADCY5, CYP2C38, CYP2C37, HRH1, MAPK8, ASIC5, CYP2C55, CYP2C54, ALOX12E, CYP2C50, TRPM8, ASIC2, MAP2K6, CYP2J5, TRPA1, CYP4A31, CYP2J8, CYP4A32, PLA2G4A, CYP4A14, TRPV1, CYP2C29, CYP2C69, CYP2C68, CYP2J11, CYP2C67, CYP2J12, CYP2C66, CYP2J13, CYP2C65, IL1B, CYP2C40, PRKCQ	39	5.33E-10	CYP4A12A, CYP4A12B, CYP4A30B, ITPR2, HTR2A, CYP2C39, CYP4A29, ADCY5, CYP2C38, CYP2C37, MAPK8, ASIC5, CYP2C55, CYP2C54, ALOX12E, CYP2C50, ASIC2, MAP2K6, CYP2J5, TRPA1, CYP4A31, CYP2J8, CYP4A10, CYP4A32, PLA2G4A, CYP4A14, TRPV1, CYP2C29, CYP2C69, CYP2C68, CYP2J11, CYP2C67, CYP2J12, CYP2C66, CYP2J13, CYP2C65, IL1B, CYP2C40, PRKCQ
mmu00140:Steroid hormone biosynthesis	35	2.82E-12	CYP3A11, CYP3A57, CYP3A59, CYP2B19, CYP3A16, CYP2C39, CYP2C38, CYP2B9, CYP2C37, CYP2D12, CYP2D34, CYP2D9, CYP2B13, CYP2C54, CYP2B10, CYP2C50, CYP1B1, SRD5A2, HSD3B3, HSD3B1, CYP3A44, CYP3A25, CYP2C29, CYP2C69, CYP2C68, CYP2C67, CYP2C66, CYP2B23, CYP2C65, CYP3A41A, CYP21A1, CYP2D40, CYP3A41B, CYP2C40, CYP2E1	35	2.35E-09	CYP3A11, CYP3A57, CYP3A59, CYP19A1, CYP2B19, CYP3A16, CYP2C39, CYP2C38, CYP2B9, CYP2C37, CYP2D12, CYP2D34, CYP2D9, CYP2C55, CYP2C54, CYP2B10, CYP2C50, SRD5A2, HSD3B3, CYP3A44, CYP3A25, CYP2C29, CYP2C69, CYP2C68, CYP2C67, CYP2C66, CYP2B23, CYP2C65, CYP3A41A, CYP21A1, CYP2D40, CYP3A41B, CYP2C40, CYP2E1, HSD3B8	38	1.59E-13	CYP3A11, CYP3A57, CYP3A59, CYP19A1, CYP2B19, CYP3A16, CYP2C39, CYP2C38, CYP2B9, CYP2C37, CYP2D12, CYP2D34, CYP2D9, CYP2B13, CYP2C55, CYP2C54, CYP2B10, CYP2C50, CYP1B1, HSD3B6, SRD5A2, HSD3B3, HSD3B1, CYP3A44, CYP3A25, CYP2C29, CYP2C69, CYP2C68, CYP2C67, CYP2C66, CYP2B23, CYP2C65, CYP3A41A, CYP21A1, CYP2D40, CYP3A41B, CYP2C40, CYP2E1
mmu04512:ECM-receptor interaction	24	3.76E-06	LAMA2, ITGA4, ITGA1, TNC, THBS2, THBS4, DSPP, SV2C, COL1A2, RELN, COL2A1, SV2B, IBSP, TNN, COL6A2, COL4A4, COL6A1, COL4A3, COL9A1, COL6A3, TNR, COL6A6, COL6A5, FREM1	33	2.51E-09	TNXB, LAMA2, LAMA1, ITGB3, LAMA3, TNC, DMP1, THBS2, THBS4, SV2C, RELN, IBSP, SV2B, TNN, TNR, ITGA4, VWF, ITGA1, MEPE, GP6, DSPP, COL2A1, COL1A2, COL4A4, COL6A2, COL4A3, COL6A1, COL6A4, COL9A1, COL6A3, COL6A6, COL6A5, FREM1	27	2.91E-07	ITGB3, LAMA3, TNC, DMP1, THBS2, THBS4, SV2C, RELN, IBSP, SV2B, TNN, TNR, ITGA4, ITGA1, MEPE, DSPP, COL2A1, COL1A2, COL4A4, COL6A2, COL6A1, COL6A4, COL9A1, COL6A3, COL6A6, COL6A5, FREM1

mmu04060:Cytokine-cytokine receptor interaction	62	8.20E-10	IL21, IFNA4, IL20, IFNA1, IL23R, IL5RA, PRL, CXCL2, CXCL5, IL18RAP, IL36A, IL36B, IFNAB, CCR9, IL12B, TNFRSF8, CCR5, CCR3, CCR2, IFNAR2, IFNA12, IFNA14, IL19, IL16, PRLR, IFNG, IL1B, IL3RA, CCL12, IL20RB, CXCR4, CSF2RA, IL1RL1, CCL8, CCL7, CCL4, IL22B, CCL2, CCL1, IL12RB2, CCR1, TNFSF18, IL33, TSLP, CD70, BMP7, IL2, ACVR2A, BMP5, BMP4, IL3, IL6, BMP2, CXCL12, FASL, IL7, TNFSF4, IL2RA, LEP, TNFSF8, IL7R, IL18R1	71	3.95E-09	IL21, IL22, BMP10, IL20, IFNA1, IL23R, IL5RA, CXCL13, CXCL14, CXCL2, CXCL5, IL18RAP, IL36A, IL36B, LEPR, IL12B, TNFRSF8, TNFSF11, CCR6, CCR5, CCR3, CCR2, IFNAR2, IFNA12, IFNA14, IFNA15, TNFRSF19, IL16, PRLR, IFNG, IL1B, IL3RA, CCL12, CCL11, IL20RB, CXCR4, CSF2RA, IL1RL1, CCL3, CCL2, CCL1, IL12RB2, CCR1, GDF10, TNFSF18, NGFR, IL33, TSLP, CD70, IFNB1, IL36G, BMP7, BMP6, IL2, ACVR2A, BMP5, GDF7, BMP4, IL3, BMP2, FASL, IL7, TNFSF4, IL2RA, LEP, IL2RB, IL9, FAS, TNFSF8, IL7R, IL18R1	55	6.00E-06	IL21, IFNA1, IL23R, IL5RA, CXCL14, CXCL2, CXCL5, IL18RAP, IL36A, IL36B, IL12B, TNFRSF8, CCR3, CCR2, IFNA11, IFNAR2, IFNA14, TNFRSF19, PRLR, IL1B, IL3RA, CCL12, CCL11, IL20RB, CXCR4, CSF2RA, IL1RL1, CCL6, CCL4, IL22B, CCL2, CCL1, IL12RB2, CCR1, GDF10, TNFSF18, IL33, TSLP, CD70, IFNB1, BMP7, BMP6, IL2, ACVR2A, BMP5, GDF7, BMP4, IL3, FASL, TNFSF4, IL2RA, FAS, TNFSF8, IL7R, IL18R1
mmu04940:Type I diabetes mellitus	19	2.38E-05	H2-EB2, INS1, H2-M9, H2-M10,4, H2-M5, H2-M10,1, PTPRN, INS2, GAD1, H2-Q6, GAD2, GZMB, H2-T3, IL2, IFNG, FASL, IL1B, IL12B, H2-M11	27	1.25E-08	CD86, INS1, H2-M9, H2-BL, H2-M5, PTPRN, INS2, H2-Q6, H2-Q7, H2-Q2, H2-Q1, IL12B, H2-EB2, H2-M10,4, H2-M10,1, GAD1, GAD2, GZMB, H2-T3, IL2, FASL, IFNG, IL1B, FAS, H2-M11, H2-Q10, H2-AB1	22	1.12E-06	H2-EB2, INS1, H2-M9, H2-M10,4, H2-M5, H2-M10,1, PTPRN, INS2, GAD1, H2-Q6, GZMB, H2-T3, H2-Q1, IL2, FASL, IL1B, IL12B, H2-M11, FAS, H2-M10,6, H2-Q10, H2-AB1
mmu05032:Morphine addiction	24	6.90E-06	GABRB3, GABRA2, GABRB2, KCNJ5, KCNJ6, GABRA6, GABRA5, PDE1A, PDE4D, CACNA1B, ADCY2, ADCY1, OPRM1, GABRG3, GABRG2, GABRG1, GABRR3, PDE11A, GNG2, PDE10A, GNG4, PDE3A, DRD1, KCNJ3	30	3.74E-07	GABRB3, GABRB2, PDE1B, PDE1A, CACNA1B, ADCY1, ADCY8, ADCY5, GABRR3, PDE11A, GNG4, DRD1, GABRD, KCNJ3, GABRA2, GABRP, KCNJ5, GABBR2, GABRA1, KCNJ6, GABRA6, GABRA5, OPRM1, GABRG3, GABRG2, GABRG1, PDE10A, PDE3A, GNB4, PDE7B	23	7.55E-05	GABRB3, GABRP, GABRB2, KCNJ5, GABBR2, GABRA1, KCNJ6, GABRA6, GABRA5, PDE1A, PDE4D, CACNA1B, OPRM1, GABRG3, GABRG2, ADCY5, GABRR3, PDE11A, GNG4, GNB4, PDE3A, DRD1, KCNJ3
mmu04514:Cell adhesion molecules	32	4.27E-04	H2-M9, CNTNAP2, H2-M5, NRXN1, H2-Q6, SDC2, CDH5, CDH4, CDH2, SLITRK1, LRRC4C, CD34, JAM2, H2-EB2, VCAM1, NEGR1, ITGA4, H2-M10,4, GLYCAM1, H2-M10,1, H2-T3, SELE, PTPRD, SELP, PTPRC, CNTN1, H2-M11, CD226, PDCD1, CLDN16, NECTIN3, NECTIN1	46	4.86E-07	CD86, H2-M9, CNTNAP2, H2-BL, H2-M5, NRXN1, H2-Q6, SDC2, H2-Q7, NRXN3, NRXN2, H2-Q2, H2-Q1, CDH5, SLITRK1, SLITRK6, SLITRK5, LRRC4C, CD34, JAM2, LRRC4B, H2-EB2, VCAM1, NEGR1, ITGA4, H2-M10,4, GLYCAM1, H2-M10,1, H2-T3, SELE, SELP, CLDN5, PTPRC, SELL, CLDN14, CNTN1, CLDN18, H2-M11, CNTN2, CD226, PDCD1, CLDN16, NECTIN3, H2-Q10, H2-AB1, NECTIN1	40	1.86E-06	H2-M9, CNTNAP2, H2-M5, NRXN1, H2-Q6, SDC2, NRXN3, H2-Q1, CDH5, CDH2, SLITRK1, SLITRK5, ICOS, LRRC4C, CD34, JAM2, LRRC4B, JAM3, H2-EB2, VCAM1, NEGR1, ITGA4, H2-M10,4, GLYCAM1, H2-M10,1, H2-T3, SELE, CD2, SELP, PTPRC, SELL, CLDN14, CNTN1, CLDN18, H2-M11, PDCD1, CLDN16, H2-M10,6, H2-Q10, H2-AB1
mmu05332:Graft-versus-host disease	15	9.02E-04	H2-EB2, H2-M9, H2-M10,4, H2-M5, H2-M10,1, H2-Q6, GZMB, H2-T3, IL2, IL6, IFNG, FASL, IL1B, H2-M11, KLRD1	23	5.27E-07	CD86, H2-EB2, H2-M9, H2-BL, H2-M10,4, H2-M5, H2-M10,1, H2-Q6, H2-Q7, GZMB, H2-Q2, H2-T3, H2-Q1, IL2, IFNG, FASL, IL1B, H2-M11, FAS, KLRD1, KLRC1, H2-Q10, H2-AB1	18	5.08E-05	H2-EB2, H2-M9, H2-M10,4, H2-M5, H2-M10,1, H2-Q6, GZMB, H2-T3, H2-Q1, IL2, FASL, IL1B, H2-M11, FAS, KLRD1, H2-M10,6, H2-Q10, H2-AB1
mmu04151:PI3K-Akt signaling pathway	63	1.64E-06	INS1, IFNA4, FLT1, IFNA1, FLT3, INS2, TNC, PRL, FGF6, IBSP, YWHAQ, TNN, IFNAB, TNR, MAGI1, IFNAR2, IFNA12, IFNA14, ITGA4, ITGA1, PRLR, EREG, COL2A1, TCL1, CREB1, COL4A4, COL6A2, COL4A3, COL6A1, IL3RA, COL6A3, COL6A6, COL6A5, LAMA2, THBS2, EFNA5, THBS4, GNG2, RELN, PPP2R3D, GNG4, NTF3, FGF20, FGF23, NTRK1, NTRK2, ANGPT4, ANGPT1, BDNF, IL2, IL3, IL6, CDK6, COL1A2, FASL, GDNF, IL7, IL2RA, FGF18, COL9A1, IL7R, FGFR2, FGF10	76	9.43E-07	INS1, FLT1, IFNA1, FLT3, ITGB3, INS2, TNC, FGF3, FGF4, GYS2, FGF6, FGF8, IBSP, FGF9, YWHAQ, TNN, TNR, MAGI1, IFNAR2, IFNA12, IFNA14, ITGA4, IFNA15, VWF, HGF, ITGA1, PRLR, EREG, COL2A1, TCL1, COL4A4, COL6A2, COL4A3, COL6A1, IL3RA, COL6A4, COL6A3, COL6A6, COL6A5, TNXB, LAMA2, LAMA1, LAMA3, THBS2, EFNA5, THBS4, RELN, PPP2R3D, GNG4, PDGFD, NTF3, FGF20, FGF23, NGFR, NTRK2, ANGPT1, BDNF, IFNB1, TCL1B4, IL2, IL3, CDK6, COL1A2, FASL, PPP2R2C, GDNF, IL7, PPP2R2B, IL2RA, IL2RB, FGF18, COL9A1, GNB4, IL7R, FGFR2, FGF10	61	7.17E-05	INS1, FLT1, IFNA1, FLT3, ITGB3, INS2, TNC, FGF4, FGF6, IBSP, FGF9, YWHAQ, TNN, PPP2R1A, TNR, IFNA11, IFNAR2, IFNA14, ITGA4, ITGA1, PRLR, EREG, COL2A1, TCL1, CREB1, COL4A4, COL6A2, COL6A1, IL3RA, COL6A4, COL6A3, COL6A6, COL6A5, LAMA3, THBS2, EFNA5, THBS4, RELN, PPP2R3D, GNG4, NTF3, FGF20, FGF23, NTRK2, ANGPT1, BDNF, IFNB1, INSR, IL2, IL3, CDK6, COL1A2, FASL, GDNF, IL2RA, FGF18, COL9A1, GNB4, IL7R, FGFR2, FGF10
mmu05320:Autoimmune thyroid disease	18	4.50E-04	H2-EB2, IFNA12, H2-M9, IFNA4, IFNA14, IFNA1, H2-M10,4, H2-M5, H2-M10,1, H2-Q6, GZMB, H2-T3, TSHR, IL2, TPO, FASL, IFNAB, H2-M11	26	9.72E-07	CD86, H2-M9, H2-BL, IFNA1, H2-M5, H2-Q6, H2-Q7, H2-Q2, H2-Q1, TPO, TSHB, IFNA12, H2-EB2, IFNA14, IFNA15, H2-M10,4, H2-M10,1, GZMB, H2-T3, IL2, TG, FASL, FAS, H2-M11, H2-Q10, H2-AB1	21	3.70E-05	IFNA11, H2-EB2, H2-M9, IFNA14, IFNA1, H2-M10,4, H2-M5, H2-M10,1, H2-Q6, GZMB, H2-T3, H2-Q1, IL2, TPO, FASL, H2-M11, FAS, H2-M10,6, TSHB, H2-Q10, H2-AB1
mmu05204:Chemical carcinogenesis - DNA adducts	25	1.26E-06	CYP3A11, CYP3A57, CYP3A59, CYP3A16, CYP2C39, CYP2C38, CYP2C37, HPGDS, CYP2C54, CYP2C50, CYP1B1, CYP3A44, CYP3A25, CYP2C29, CYP2C69, CYP2C68, CYP2C67, CYP2C66, NAT1, CYP2C65, CYP3A41A, NAT3, CYP3A41B, CYP2C40, CYP2E1	27	9.02E-06	CYP3A11, CYP3A57, CYP3A59, CYP3A16, CYP2C39, CYP2C38, CYP2C37, HPGDS, CYP2C55, CYP2C54, CYP2C50, CBR1, GSTO2, CYP3A44, CYP3A25, CYP2C29, CYP2C69, CYP2C68, CYP2C67, CYP2C66, NAT1, CYP2C65, CYP3A41A, NAT3, CYP3A41B, CYP2C40, CYP2E1	26	1.39E-06	CYP3A11, CYP3A57, CYP3A59, CYP3A16, CYP2C39, CYP2C38, CYP2C37, HPGDS, CYP2C55, CYP2C54, CYP2C50, CYP1B1, CYP3A44, CYP3A25, CYP2C29, CYP2C69, CYP2C68, CYP2C67, CYP2C66, NAT1, CYP2C65, CYP3A41A, NAT3, CYP3A41B, CYP2C40, CYP2E1
mmu05330:Allograft rejection	13	0.0078	H2-EB2, H2-M9, H2-M10,4, H2-M5, H2-M10,1, H2-Q6, GZMB, H2-T3, IL2, IFNG, FASL, IL12B, H2-M11	21	9.34E-06	CD86, H2-EB2, H2-M9, H2-BL, H2-M10,4, H2-M5, H2-M10,1, H2-Q6, H2-Q7, GZMB, H2-Q2, H2-T3, H2-Q1, IL2, IFNG, FASL, IL12B, H2-M11, FAS, H2-Q10, H2-AB1	17	1.84E-04	H2-EB2, H2-M9, H2-M10,4, H2-M5, H2-M10,1, H2-Q6, GZMB, H2-T3, H2-Q1, IL2, FASL, IL12B, H2-M11, FAS, H2-M10,6, H2-Q10, H2-AB1
mmu04024:cAMP signaling pathway	42	1.42E-05	GRIA1, POPDC3, GRIA2, RYR2, ADCY2, CACNA1C, ADCY1, HTR4, PLD1, GLI3, GRIN2A, EDNRA, CNGA3, DRD1, DRD2, BVES, 4930544G11RIK, GRIA4, DRD5, ABCC4, GHSR, LHCGR, EDN3, HTR1F, BDNF, PDE4D, FSHB, HTR1A, ATP2B1, GRIN2B, TSHR, GRIN1, MAPK10, TIAM1, ADCYAP1, CREB1, PDE10A, SST, FSHR, PDE3A, CNGB3, VIP	50	1.12E-05	RYR2, OXTR, HTR4, GLI3, EDNRA, CNGA3, BVES, TSHB, VAV3, GHSR, LHCGR, EDN3, FSHB, ARAP3, TIAM1, ADCYAP1, FSHR, CNGB3, MYL9, GRIA1, POPDC3, GRIA2, ADCYAP1R1, ADCY1, PLD1, ADCY8, CRHR1, ADCY5, MC2R, MAPK8, GRIN2A, DRD1, DRD2, 4930544G11RIK, DRD5, GRIA4, GABBR2, BDNF, HTR1F, HTR1A, GCG, GRIN2B, GRIN1, GRIN3A, PDE10A, SST, CAMK4, PDE3A, VIP, KCNK2	40	3.66E-04	GRIA1, POPDC3, GRIA2, RYR2, ADCYAP1R1, HTR4, PLD1, GLI3, CRHR1, ADCY5, GRIN2A, EDNRA, MAPK8, DRD1, DRD2, BVES, 4930544G11RIK, TSHB, DRD5, GABBR2, GHSR, LHCGR, EDN3, HTR1F, BDNF, PDE4D, FSHB, HTR1A, ATP2B2, GCG, GRIN2B, GRIN1, TIAM1, ADCYAP1, CREB1, SST, PDE3A, CNGB3, VIP, KCNK2

Supplementary Table 3. RNA-Seq. Top 20 KEGG pathways in $KRAS^{G12D}$ and $KRAS^{G12D}EZH2^{\Delta SET}$ vs Control

$KRAS^{G12D}$ vs control	Count	pValue	Genes
Maturity onset diabetes of the young	7	5.17E-06	NEUROD1, INS1, RFX6, INS2, IAPP, HNF1B, HES1
Renin-angiotensin system	6	0.0004	KLK1B8, KLK1B11, KLK1B3, CMA1, KLK1B24, THOP1
Endocrine and other factor-regulated calcium reabsorption	7	0.0006	KLK1B8, KLK1B11, PLCB4, VDR, KLK1B3, KLK1B24, ATP2B2
Insulin secretion	7	0.0036	SNAP25, INS1, ADCYAP1R1, PLCB4, ABCC8, INS2, KCNN3
Type I diabetes mellitus	6	0.0058	INS1, H2-EB1, PTPRN, INS2, GAD1, ICA1
Pancreatic secretion	7	0.0142	PNLIPRP2, PLCB4, 1810009J06RIK, ATP2A3, ATP2B2, AMY1, GM2663
Neuroactive ligand-receptor interaction	14	0.0168	GRIA2, LYNX1, VIPR1, ADCYAP1R1, GIPR, 1810009J06RIK, GABRA3, F2, SSTR2, PYY, GAL, UCN3, IAPP, GM2663
African trypanosomiasis	4	0.0290	PLCB4, HBA-A2, HBA-A1, THOP1
MAPK signaling pathway	11	0.0334	DUSP4, DUSP5, INS1, DUSP3, KLK1B4, MECOM, TGFB3, DUSP1, INS2, FOS, GADD45G
Thyroid hormone synthesis	5	0.0383	TTR, PLCB4, IYD, ALB, DUOX2
Parathyroid hormone synthesis. secretion and action	6	0.0494	EGR1, PLCB4, VDR, MMP15, WNK4, FOS

$KRAS^{G12D}EZH2^{\Delta SET}$ vs control	Count	pValue	Genes
Circadian rhythm	8	0.0002	PER2, DBP, PER3, NFIL3, CRY2, BHLHE41, NR1D2, CLOCK
Cell adhesion molecules	17	0.0011	NLGN2, NRXN1, H2-Q2, SPN, PTPRD, CDH5, PTPRC, CDH2, SELL, CLDN9, H2-DMB1, CNTN1, SLITRK6, PECAM1, ITGA6, CD22, JAM3
Insulin secretion	11	0.0014	SNAP25, INS1, ADCYAP1R1, PLCB4, PRKCB, ABCC8, INS2, ATP1A3, GCG, FFAR1, KCNN3
Cytoskeleton in muscle cells	19	0.0028	FBN2, SPTBN4, SYNPO2, ITGA1, TPM1, FN1, ATP1A3, FMN2, HSPG2, ENO3, ACTG1, CSRP1, COL4A2, COL4A1, COL5A3, ANKRD1, TCAP, ITGA6, TLN2
Maturity onset diabetes of the young	6	0.0030	NEUROD1, INS1, RFX6, INS2, HNF1B, IAPP
Metabolic pathways	83	0.0031	PNMT, PI4K2B, GALNT11, PYGB, GALNT14, GALNT16, PIGO, GALNT15, GDA, OGDHL, GPT, ENO3, DHTKD1, GMPPB, SMPD3, FADS2, SMPD2, GUK1, CYP2B10, NAT8F1, FPGS, SCD2, TK2, LARGE2, GLUL, ARG2, ALG6, ELOVL2, ENTPD5, SPHK1, AMT, TALDO1, PIPOX, CMBL, POMGNT2, GBGT1, INMT, PLCB4, IVD, CHDH, PDE5A, AMY1, ALDOB, L2HGDH, ST6GALNAC4, ISYNA1, OAT, PDE1C, GSTP1, ALOX15, GCNT1, ODC1, MRI1, GMPR, PAPSS2, DGLUCY, MAN1C1, INPP5J, HYI, ATP6V0A4, GPAT3, B4GALNT1, PRODH, RIMKLB, GGT5, HSD3B7, UGT2B34, GSTM1, GAD1, GFPT2, PYCR1, FMO1, MTHFR, DHCR24, FMO2, B3GALT5, FMO5, PAFAH2, G6PC2, GGCX, SARDH, KYAT1, DHCR7
Calcium signaling pathway	20	0.0033	PDE1C, PTGER1, PRKCB, SPHK1, F2R, ATP2A3, VEGFB, NFATC2, CACNA1A, ATP2B2, CACNA1E, PPP3CA, PLCB4, KLK1B4, P2RX3, KDR, FGFR3, SLC25A4, FGFR2, FGF21
PI3K-Akt signaling pathway	25	0.0058	INS1, LAMC3, INS2, VTN, KLK1B4, YWHAQ, KDR, TNR, NRTN, FGF21, SYK, ITGA1, F2R, VEGFB, FN1, PRLR, G6PC2, COL4A2, COL4A1, KITL, ITGA6, PKN1, PIK3AP1, FGFR3, FGFR2
ECM-receptor interaction	10	0.0061	VTN, COL4A2, SV2A, LAMC3, COL4A1, ITGA1, FN1, TNR, ITGA6, HSPG2
Mucin type O-glycan biosynthesis	6	0.0063	GALNT11, GALNT14, GALNT16, GALNT15, GCNT1, ST6GALNAC4
Rap1 signaling pathway	17	0.0068	INS1, DOCK4, PRKCB, INS2, F2R, VEGFB, ACTG1, GNAO1, PLCB4, KLK1B4, KITL, RAC2, KDR, TLN2, FGFR3, FGFR2, FGF21
Taurine and hypotaurine metabolism	5	0.0084	GGT5, GAD1, FMO1, FMO2, FMO5
MAPK signaling pathway	21	0.0089	INS1, GADD45B, PRKCB, CACNA2D1, INS2, VEGFB, CACNA1A, ARRB1, MAPK8IP2, CACNA1E, RELB, PPP3CA, KLK1B4, KITL, RAC2, KDR, MAP3K10, NRTN, FGFR3, FGFR2, FGF21
Focal adhesion	16	0.0091	LAMC3, PRKCB, ITGA1, VEGFB, FN1, EMP1, ACTG1, VTN, COL4A2, COL4A1, RAC2, KDR, TNR, ITGA6, TLN2, DOCK1
Neuroactive ligand-receptor interaction	24	0.0215	GRIA2, LYNX1, GABBR1, ADCYAP1R1, PTGER1, GIPR, 1810009J06RIK, F2R, GCG, PRLR, SSTR3, ADRA2A, APLN, PYY, GAL, UCN3, P2RX3, UCN2, NPY, APLNR, PPY, IAPP, S1PR3, GM2663
Endocrine and other factor-regulated calcium reabsorption	7	0.0263	KLK1B8, PLCB4, KLK1B3, PRKCB, KLK1B5, ATP1A3, ATP2B2
cGMP-PKG signaling pathway	13	0.0286	INS1, INS2, ATP2A3, NFATC2, ATP1A3, ATP2B2, IRS2, ADRA2A, PPP3CA, RGS2, PLCB4, PDE5A, SLC25A4
Pancreatic secretion	10	0.0289	SLC12A2, PLCB4, PRKCB, 1810009J06RIK, ATP2A3, ATP1A3, ATP2B2, AMY1, GM2663, SLC4A4
Type II diabetes mellitus	6	0.0332	INS1, ABCC8, INS2, CACNA1A, IRS2, CACNA1E
Carbohydrate digestion and absorption	6	0.0359	G6PC2, PLCB4, PRKCB, ATP1A3, AMY1, SLC2A5

Supplementary Table 4. RNA-Seq Top 20 KEGG pathways

$KRAS^{G12D}$ vs $KRAS^{G12D}EZH2^{\Delta SET}$	Count	pValue	Genes
Phagosome	17	2.61672352526268E-08	COLEC12, H2-EB1, C1RA, NCF2, H2-DMA, H2-Q6, H2-Q7, CYBB, H2- AA, CORO1A, CTSS, ACTG1, C3, FCGR3, H2-DMB1, FCGR2B, H2- AB1
Staphylococcus aureus infection	13	7.42826882949764E-07	C1QB, CFD, C1QA, H2-EB1, C1RA, H2-DMA, H2-AA, C3, FCGR3, H2-DMB1, FCGR2B, C1QC, H2-AB1
Cell adhesion molecules	15	8.00862628447581E-07	H2-EB1, CADM3, PTPRS, SDC2, H2-DMA, H2-Q6, H2-Q7, H2-AA, VSIR, SPN, PTPRC, SELL, H2-DMB1, CD34, H2-AB1
Antigen processing and presentation	11	9.87292550762858E-07	CD74, H2-EB1, H2-DMA, H2-DMB1, H2-Q6, H2-Q7, IFI30, H2-AA, B2M, CTSS, H2-AB1
Tuberculosis	14	4.90052922069888E-06	CD74, H2-EB1, SYK, H2-DMA, LSP1, H2-AA, CORO1A, CTSS, TLR1, C3, FCGR3, H2-DMB1, FCGR2B, H2-AB1
Leishmaniasis	9	0.000012985899874562	C3, FCGR3, H2-EB1, NCF2, H2-DMA, H2-DMB1, CYBB, H2-AA, H2- AB1
Asthma	6	0.000037324831741165	H2-EB1, CCL11, H2-DMA, H2-DMB1, H2-AA, H2-AB1
Epstein-Barr virus infection	14	0.000060873988757194	H2-EB1, SYK, GADD45B, H2-DMA, H2-Q6, H2-Q7, H2-AA, GADD45G, MYC, H2-DMB1, CD19, VIM, B2M, H2-AB1
Viral myocarditis	9	0.000111354947409876	H2-EB1, H2-DMA, H2-DMB1, H2-Q6, H2-Q7, RAC2, H2-AA, H2-AB1, ACTG1
Hematopoietic cell lineage	9	0.000111354947409876	H2-EB1, H2-DMA, H2-DMB1, CD19, CD37, H2-AA, CSF2RA, CD34, H2- AB1
Systemic lupus erythematosus	11	0.000123835152052183	C1QB, C3, C1QA, FCGR3, H2-EB1, C1RA, H2-DMA, H2-DMB1, H2- AA, H2-AB1, C1QC
Allograft rejection	7	0.000342889498065715	H2-EB1, H2-DMA, H2-DMB1, H2-Q6, H2-Q7, H2-AA, H2-AB1
Graft-versus-host disease	7	0.000342889498065715	H2-EB1, H2-DMA, H2-DMB1, H2-Q6, H2-Q7, H2-AA, H2-AB1
Type I diabetes mellitus	7	0.000624473914625959	H2-EB1, H2-DMA, H2-DMB1, H2-Q6, H2-Q7, H2-AA, H2-AB1
Complement and coagulation cascades	8	0.000680801919823974	CFD, C1QB, C3, C1QA, C1RA, SERPING1, PLAT, C1QC
Autoimmune thyroid disease	7	0.00121829321544103	H2-EB1, H2-DMA, H2-DMB1, H2-Q6, H2-Q7, H2-AA, H2-AB1
Influenza A	10	0.00162210489934329	IL33, H2-EB1, H2-DMA, H2-DMB1, 1810009J06RIK, TNFSF10, H2-AA, SLC25A4, H2-AB1, ACTG1
Fluid shear stress and atherosclerosis	9	0.00230892358937451	NCF2, DUSP1, GSTO1, SDC2, MMP2, RAC2, PLAT, KLF2, ACTG1
Th1 and Th2 cell differentiation	7	0.00258860511691217	JAG2, H2-EB1, MAF, H2-DMA, H2-DMB1, H2-AA, H2-AB1
Inflammatory bowel disease	6	0.00284876715093882	H2-EB1, MAF, H2-DMA, H2-DMB1, H2-AA, H2-AB1

Supplementary Table 5. RNA-Seq Top 20 GseaGO pathways

MKE vs *Mist1*$^{creERT/-}$ *KRAS*^{G12D}	Count	padj Value	Genes
GO:0006334 - nucleosome assembly	33	1.32E-06	H4c14/H4c12/H4c8/H1f4/H3c4/H4f16/H3c6/H1f3/H1f5/H2bc3/H4c3/H3c1/H3c7/H4c1/H2bc7/H3c3/H3c2/H4c4/H4c18/H4c6/H1f1
GO:0046034 - ATP metabolic process	117	1.05E-05	Atp5k/ATP8/Cox6a1/Atp5j2/Ddit4/Atp5e/Trem2/Cox5b/COX2/Cox4i2/Ier3/Cox7a1/Antkmt/Atp5g3/Chchd10/Gck/ND4L/Tspo/Ins2/ATP6/Ndufb6/Uqcrq/Pgam1/Cyc1/Mif/Eif6/Galk1/COX3/Ndufc2/Gpd1/Uqcr10/Eno3/Ndufv3/Uqcc2/Atp5d/Nupr1/Ldhd/Sdhc/Ndufa8/Khk/Ndufb9/Ndufb8/Ndufa12/Slc25a33/Pink1/Ak1/CYTB/Atp1a2/Aldoc/Eno2/Cox5a/Guk1/Uqcrfs1/Gadd45gip1/Sdhd/Coq7/Atp5g1/Atp5j/Uqcc3/Ndufa7/Ndufs8/Coq9/Park7/Uqcrh/Uqcrc1/Cox4i1/Cox7a2/Flcn/Bad/Slc25a23/Pgk1/Ak2/Aldoa/Ndufv1/Bcl2l1/Taz/Iscu
GO:0032963 - collagen metabolic process	51	1.20E-05	Retn/Mmp23/Scx/Mmp2/Adam15/Ccn2/Rgcc/Mmp9/Mfap4/Serpinh1/Rcn3/Id1/Eng/Prdx5/Cygb/P3h4/Mmp28/Mmp11/Col1a2/Mrc2/Col1a1/Adamts2/P3h3/Ctsk/Vim/Wnt4/Pdgfb/Mmp19/Rapgef3/Mmp14/Col5a1/Tnxb/Bmp4/Tgfb3/Smpd3/Mmp15/Ppard
GO:0042060 - wound healing	132	1.20E-05	Treml1/Vkorc1/Timp1/Insl3/F2rl3/Anxa8/Cldn4/Bnc1/Wfdc1/Alox15/Gpx1/Aqp1/Tmeff2/Clec10a/Ccm2l/Tspan8/Pf4/Serping1/Fgf1/Entpd2/Ins2/Thbd/Anxa2/BC024139/Tnfrsf12a/Cd34/Cadm4/Apoe/Cav1/Smoc2/Ajuba/Eng/Bloc1s4/Selp/Fbln1/C1qtnf1/Cdkn1a/Anxa1/Proc/Cd151/Fkbp10/Cldn3/Col1a1/Cela2a/F3/Adrb1/Bloc1s3/Plau/Gata2/Wnt4/Pdgfb/Col3a1/Gas6/Erbb2/Acvrl1/Col5a1/Srf/Hmox1/Gnas/Axl/Evpl/Wnt5a/Serpine2/Vegfb/Actg1/Plet1/Ppard
GO:0048568 - embryonic organ development	163	1.92E-05	Xist/Tbx4/Rnf112/Tcf21/Gjb5/Snai1/Nkx3-2/Ccdc103/Cebpb/Id3/Twist1/Sox18/Crb2/Gli1/Hand2/Mdfi/Wnt7b/Wnt11/Fzd2/Mfap2/Wnt2/Pbx4/Lif/Gas1/Adm/Col13a1/Vash1/Rarres2/Junb/Pkdcc/Wnt9a/Hoxb6/Cebpa/Erf/Mfap5/Eng/Rbp4/Osr1/Tbx2/Hoxb7/Cdkn1c/Epha2/Epn1/Slc39a3/Cdk20/Smo/Naglu/Plcd3/Sox17/Gja5/Gata2/Edn1/Socs3/Pdgfb/Aldh1a2/Mmp14/Krt8/Sod1/Chst11/Srf/Gnas/Bmp4
GO:0034728 - nucleosome organization	50	2.98E-05	H4c14/H4c12/H4c8/H1f4/H3c4/H4f16/H3c6/H1f3/H1f5/H2bc3/H4c3/H3c1/H3c7/H4c1/H2bc7/H3c3/H3c2/H4c4/H4c18/H4c6/H1f1
GO:0006119 - oxidative phosphorylation	63	2.98E-05	Cox6a1/Atp5j2/Cox5b/COX2/Cox4i2/Cox7a1/Antkmt/Chchd10/ND4L/ATP6/Ndufb6/Uqcrq/Cyc1/COX3/Ndufc2/Uqcr10/Ndufv3/Uqcc2/Atp5d/Nupr1/Sdhc/Ndufa8/Ndufb9/Ndufb8/Ndufa12/Slc25a33/Pink1/CYTB/Cox5a/Uqcrfs1/Gadd45gip1/Sdhd/Coq7/Atp5j/Uqcc3/Ndufa7/Ndufs8/Coq9/Park7/Uqcrh/Uqcrc1/Cox4i1/Cox7a2/Slc25a23/Pgk1/Ndufv1/Taz/Iscu/Ppif/Ndufs6/Shmt2/Atp5pb/Dnajc15
GO:0006790 - sulfur compound metabolic process	103	2.98E-05	Gstp2/Pdk4/Gstm3/Gpx1/Dgat2/Bola2/Slc27a3/Ggt6/Chpf/Chst14/Chac1/Ciapin1/Chst7/Nat8/Gpx3/Gpx4/Cdo1/Mpst/Gamt/Tst/Dpep1/Hmgcl/Gstm2/Iba57/Dcn/Sod1/Chst11/Gstm6/Bgn/Fasn/Gnmt/Acaa2/Mvd/Ppcs/Pemt/Spock2/Dgat1/Ciao2b/Mlycd/Ggt5/Sulf1/Phgdh/Hscb/Mpc2/Gsta4/Apip/Comt/Gstt1/Pmvk/Mat1a/Acot7/Chst12/Gstk1/Glrx3/Park7/Isca2/Gstp1/Gstm1/Mgst1/Idua/Oplah/Hmgn5/Csad/Tpst2/Mthfr/Iscu/Acot8/Xylt2/Suox/Tpst1/Nubp2/Pcyox1l
GO:0006335 - DNA replication-dependent nucleosome assembly	14	2.98E-05	H4c14/H4c12/H4c8/H3c4/H4f16/H3c6/H4c3/H3c1/H4c1/H3c2/H4c4/H4c18/H4c6
GO:0034723 - DNA replication-dependent nucleosome organization	14	2.98E-05	H4c14/H4c12/H4c8/H3c4/H4f16/H3c6/H4c3/H3c1/H4c1/H3c2/H4c4/H4c18/H4c6
GO:0072593 - reactive oxygen species metabolic process	89	3.44E-05	Hbb-bt/Cryab/Ptgis/Pdk4/Hba-a1/Ddit4/Sod3/COX2/Gpx1/Ndufaf2/Spr/Ier3/Ngfr/Romo1/Hbb-b1/Tspo/Nos3/Ccn2/Ins2/Ndufa13/Ddah2/Cd34/Klf2/Cav1/Sesn1/Fbln5/Cyba/Prdx5/Gpx3/Agtr1a/Eif6/Cdkn1a
GO:0065004 - protein-DNA complex assembly	59	3.64E-05	H4c14/Mcmdc2/H4c12/H4c8/H1f4/H3c4/H4f16/H3c6/H1f3/H1f5/H2bc3/H4c3/H3c1/H3c7/H4c1/H2bc7/H3c3/H3c2/H4c4/H4c18/H4c6/H1f1
GO:1901342 - regulation of vasculature development	147	9.09E-05	Sfrp2/Ptgis/Pgf/Rhob/Ccl24/Aqp1/Glul/Ngfr/Angpt4/Ecm1/Lif/Cma1/Hspb1/Adm/Wt1/Aplnr/Pf4/Vash1/Tmem100/Fgf1/Emc10/Nos3/Tnfrsf12a/Cd34/Rgcc/Serpinf1/Klf2/Mmp9/Smoc2/Sphk1/Adm2/Id1/Eng/Fbln5/Rnh1/Ramp2/Agtr1a/Anxa1/Sparc/Tie1/Anxa3/Ptn/Epha2/Epn1/F3/Card10/Bmper/Thbs2/Cela1/Grn/Ccl11/Gata2/Wnt4/Hdac7/Lrg1/Pdgfb/Rapgef3/Acvrl1/Cib1/Dcn/Reck/Hmox1/Mapk7/Bmp4/Naxe/Srpx2/Wnt5a/Klf4/Vegfb
GO:0030198 - extracellular matrix organization	126	9.62E-05	Eln/Sfrp2/Loxl1/Adamtsl5/Col16a1/Mmp23/Cma1/Vit/Papln/Wt1/Scx/Col13a1/Fbln2/Mmp2/Ccn2/Anxa2/Dpt/Rgcc/Mmp9/Pmp22/Col18a1/Mfap4/Aebp1/Serpinh1/Cav1/Smoc2/Eng/Fbln5/Ramp2/Fbln1/P3h4/Mmp28/Mmp11/Col5a3/Kazald1/Fkbp10/Col1a2/Col1a1/Adamts2/Olfml2b/Loxl2/Adamtsl4/Apbb1/Col3a1/Mmp19/Mmp14/Ccdc80/Col5a1/Reck/Vtn/Efemp2/Col5a2/Tnxb/Fgfr4/Phldb1/Spock2/Hpn/Smpd3/Lamb2/Mmp15/Loxl3/Sulf1/Cav2/Emilin1/Spint2/Crtap/Ddr1/Ltbp3/Adamtsl1/Lum/Adamts10/Nid1/Scara3/Col4a2/Lamb3/Lamb1/Idua/Antxr1/Ercc2/Col4a1/Qsox1/Flot1/Washc1/Crispld2/Prdx4/Aplp1/Gfod2/Col14a1/Colgalt1/Col15a1/Tnfrsf1a
GO:0043062 - extracellular structure organization	126	9.62E-05	Eln/Sfrp2/Loxl1/Adamtsl5/Col16a1/Mmp23/Cma1/Vit/Papln/Wt1/Scx/Col13a1/Fbln2/Mmp2/Ccn2/Anxa2/Dpt/Rgcc/Mmp9/Pmp22/Col18a1/Mfap4/Aebp1/Serpinh1/Cav1/Smoc2/Eng/Fbln5/Ramp2/Fbln1/P3h4/Mmp28/Mmp11/Col5a3/Kazald1/Fkbp10/Col1a2/Col1a1/Adamts2/Olfml2b/Loxl2/Adamtsl4/Apbb1/Col3a1/Mmp19/Mmp14/Ccdc80/Col5a1/Reck/Vtn/Efemp2/Col5a2/Tnxb/Fgfr4/Phldb1/Spock2/Hpn/Smpd3/Lamb2/Mmp15/Loxl3/Sulf1/Cav2/Emilin1/Spint2/Crtap/Ddr1/Ltbp3/Adamtsl1/Lum/Adamts10/Nid1/Scara3/Col4a2/Lamb3/Lamb1/Idua/Antxr1/Ercc2/Col4a1/Qsox1/Flot1/Washc1/Crispld2/Prdx4/Aplp1/Gfod2/Col14a1/Colgalt1/Col15a1/Tnfrsf1a
GO:0045333 - cellular respiration	79	0.000111799	Cox6a1/Cox5b/COX2/Cox4i2/Ndufaf2/Sdhaf4/Bax/ND4L/Nfatc4/Ndufb6/Uqcrq/Cyc1/Ndufs7/Cisd1/COX3/Bloc1s1/Ndufc2/Ndufa5/Gpd1/Uqcr10/Ndufv3/Atp5d/Chchd4/Sdhc/Ndufa8/Ndufb9/Slc25a22/Ndufb8/Ndufa12/Pink1/ND1/CYTB/Nop53/Sirt3/Cox5a/Uqcrfs1/Sdhd/Coq7/Uqcc3/Ndufa7/Ndufs8/Sdhb/Bnip3/Coq9/Park7/Uqcrh/Uqcrc1/Coq10a/Cox4i1/Flcn/Slc25a23/Ndufv1/Taz/Iscu/Ndufs6/Oxa1l/Mdh2/Shmt2/Dnajc15/Mdh1/Ndufs4/Dguok/ND2
GO:0050727 - regulation of inflammatory response	109	0.000111799	Hamp/Ptgis/Cebpb/C2cd4b/Trem2/Wfdc1/Ccn3/Gpx2/Ccl24/Alox15/Gpx1/Ier3/Trpv4/Il17d/Per1/Cma1/Ins2/Ctla2a/Serpinf1/Pmp22/Cebpa/Metrnl/Apoe/Sphk1/Mif/Anxa1/Proc/Gpx4/Rps19/Rhbdd3/Gprc5b/Ndufc2/Tff2/Aoc3/Fabp4/C1qtnf12/Grn/Ddt/Gps2/Zp3/Il17rc/Socs3/Alox5ap/Zfp36/Nupr1/Tgm2/Sod1/Psmb4/Ace/Fndc4/Tradd/Pycard/Wnt5a
GO:0031497 - chromatin assembly	56	0.000177823	H4c14/H4c12/H4c8/H1f4/H3c4/H4f16/H3c6/H1f3/H1f5/H2bc3/H4c3/H3c1/H3c7/H4c1/H2bc7/H3c3/H3c2/H4c4/H4c18/H4c6/H1f1

GO:0033108 - mitochondrial respiratory chain complex assembly	46	0.000177823	Ndufa2/Sdhaf4/Ndufa11/Ndufa6/Cox14/Ndufaf8/Ndufb10/Ndufb7/Ndufa13/Ndufb11/Ndufb6/Ndufa1/Ndufa3/Ndufs7/Sdhaf1/Ndufc1/Ndufb3/Ndufc2/1190007I07Rik/Ndufa5/Uqcr10/Uqcc2/Ndufaf3/Chchd4/Ndufa8/Ndufb9/Ndufb8/Slc25a33
GO:0042773 - ATP synthesis coupled electron transport	38	0.000239037	Cox6a1/Cox5b/COX2/Cox4i2/ND4L/Ndufb6/Uqcrq/Cyc1/COX3/Ndufc2/Uqcr10/Ndufv3/Sdhc/Ndufa8/Ndufb9/Ndufb8/Ndufa12/Pink1/CYTB/Cox5a/Uqcrfs1/Sdhd/Coq7/Uqcc3/Ndufa7/Ndufs8/Coq9/Park7/Uqcrh/Uqcrc1/Cox4i1/Ndufv1/Taz/Iscu/Ndufs6/Dnajc15/Dguok/ND2

Supplemental Table 6A. Mouse nomenclature

Genotype	Name
C57Bl6	Control
$\mathit{Mist1}^{creERT/+}$	Control
$\mathit{Mist1}^{creERT/+}\mathit{Ezh2}^{\Delta SET/\Delta SET}$	$\mathit{EZH2}^{\Delta SET}$
$\mathit{Mist1}^{creERT/+}\mathit{KRAS}^{LSL\text{-}G12D}$	KRAS^{G12D}
$\mathit{Mist1}^{creERT/+}\mathit{KRAS}^{LSL\text{-}G12D}\mathit{Ezh2}^{\Delta SET/\Delta SET}$	$\mathit{KRASG}^{12D}\mathit{EZH2}^{\Delta SET}$
$\mathit{Mist1}^{creERT/creERT}\mathit{KRAS}^{LSL\text{-}G12D}$	$\mathit{Mist1}^{creERT/\text{-}}\mathit{KRAS}^{G12D}$
$\mathit{Mist1}^{creERT/creERT}\mathit{KRAS}^{LSL\text{-}G12D}\mathit{Ezh2}^{\Delta SET/\Delta SET}$	*MKE*

Supplemental Table 6B. Primers used for genotyping

Gene	Mutation	Forward	Reverse	Amplicon length
Mist1creERT	WT	5'-GGTTTAAGCAAATTGTCAAGTACGG 3'	5'-GAAGCATTTTCCAGGTATGCTCAG 3'	720 bp
	Cre-ERT	5'-ATAGTAAGTATGGTGGCGGTCAGCG 3'	5'-GAAGCATTTTCCAGGTATGCTCAG 3'	520 bp
KRAS	WT	5'-GTCTTTCCCCAGCACAGTGC 3'	5'-AGCTAGCCACCATGGCTTGAGTAAGTCTGCA 3'	650 bp
	G12D	5'-CTCTTGCCTACGCCACCAGCTC 3'	5'-AGCTAGCCACCATGGCTTGAGTAAGTCTGCA 3'	550 bp
EZH2	WT	5'-AGACCCCTGGGGCTTAATCT 3'	5'-CCAAGACAGGCTCTTGAGGG 3'	523 bp
	*Δ*SET			563 bp

Supplemental Table S7. RT-qPCR primers

Gene	Forward	Reverse
Mrpl1	5'-TTGGATATGCCAAGTGACCA2-3'	5'-GCTTCTGCCGTTTGAGTTTC-3'
Ptgs2	5'-AGGACTCTGCTCACGAAGGA-3'	5'-TCATACATTCCCCACGGTTT-3'
