# Supplementary material for: EZH2 deletion does not affect acinar regeneration but restricts progression to pancreatic cancer in mice
Source: JCI Insight. 2024 Dec 31;10(3):e173746. doi: 10.1172/jci.insight.173746 (PMC11948588; doi:10.1172/jci.insight.173746)

Full unedited gel for Figure 5B.

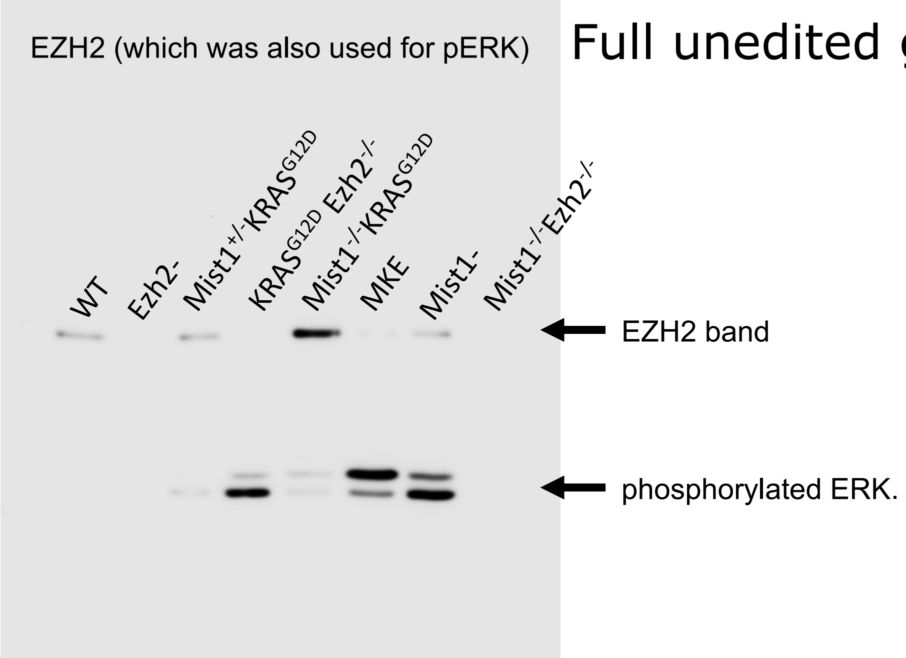

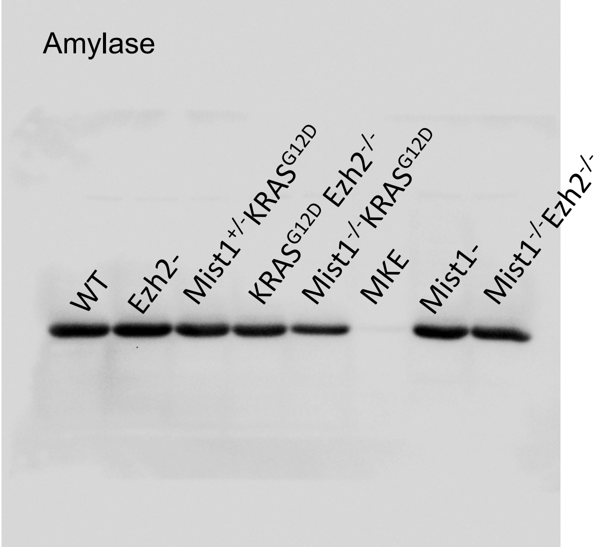

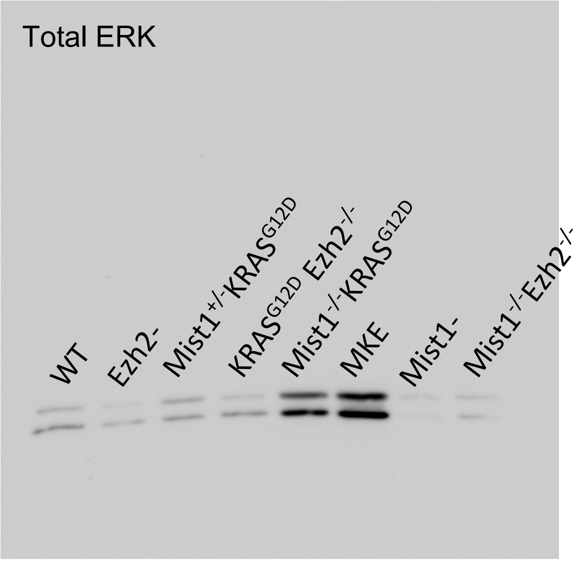

Full unedited gel for EZH2 blots in FigureS3B.

EZH2

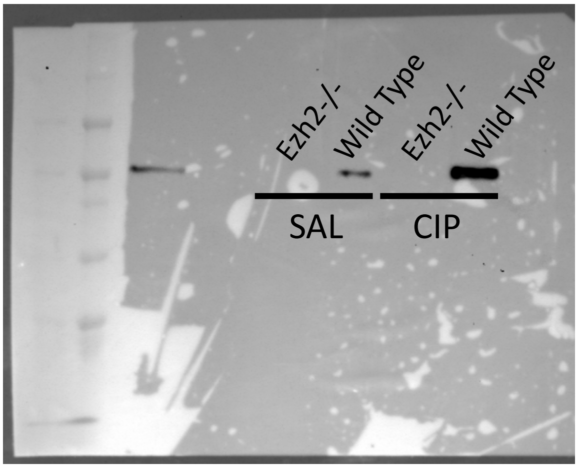

Supplement: Unedited blot and gel images [file jciinsight-10-173746-s133.pdf]
